# Preliminary Characterization and *In Vivo* Studies of Structurally Identical ^18^F- and ^125^I-Labeled Benzyloxybenzenes for PET/SPECT Imaging of *β*-Amyloid Plaques

**DOI:** 10.1038/srep12084

**Published:** 2015-07-14

**Authors:** Yanping Yang, Xiaoyang Zhang, Mengchao Cui, Jinming Zhang, Zhide Guo, Yesen Li, Xianzhong Zhang, Jiapei Dai, Boli Liu

**Affiliations:** 1 Key Laboratory of Radiopharmaceuticals, Ministry of Education, College of Chemistry, Beijing Normal University, Beijing 100875, P. R. China; 2 Department of Nuclear Medicine, Chinese PLA General Hospital, Beijing 100853, P. R. China; 3 Center for Molecular Imaging and Translational Medicine, State Key Laboratory of Molecular Vaccinology and Molecular Diagnostics, School of Public Health, Xiamen University, Xiamen 361102, China; 4 Wuhan Institute for Neuroscience and Neuroengineering, South-Central University for Nationalities, Wuhan 430074, P. R. China

## Abstract

With the assistance of molecular docking and 3D-QSAR models established previously, structurally identical ^18^F- and ^125^I-labeled benzyloxybenzene derivatives were designed to achieve the early detection of A*β* plaques by PET/SPECT imaging. In competition binding assay, ligands 7a and 12a displayed high binding affinities to A*β*_42_ aggregates with *K*_i_ values of 19.5 nM and 23.9 nM, respectively. Specific plaque labeling was observed on the *in vitro* autoradiography of brain sections from AD patients and Tg mice. In biodistribution, [^125^I]7a, [^18^F]7a, [^125^I]12a and [^18^F]12a all exhibited high initial brain uptakes (>5% ID/g at 2 min). [^125^I]7a and [^125^I]12a cleared fast from the normal brain regions, while corresponding [^18^F]7a and [^18^F]12a showed slow washout rates. Dynamic microPET/CT and microSPECT/CT imaging data in normal ICR mice were in accordance with *in vivo* biodistribution results. *In vivo* metabolism results indicated that the different clearance profiles between the structurally identical ^18^F- and ^125^I-labeled tracers could be attributed to different biochemical characteristics of the radiometabolites. Radioiodinated benzyloxybenzene derivatives exhibited good *in vivo* biostability in brain. Ex vivo autoradiography further confirmed the strong *in vivo* A*β* labeling ability of [^125^I]7a. These new fluorinated and iodinated benzyloxybenzenes can develop into PET/SPECT dual imaging agents targeting A*β* plaques.

Alzheimer’s disease (AD) is a neurodegenerative dementia condition in which the irreversible loss of neurons leads to progressive deterioration in cognitive function. AD is widely thought to be caused by protein abnormalities, the build-up of *β*-amyloid (A*β*) in extracellular plaques and the disintegration of tau protein in intracellular neurofibrillary tangles (NFTs)^1^,^2^. Substantial evidence indicates that the excessive production of A*β* peptides and the following aggregation to yield A*β* plaques have definite correlates with the initiation of AD pathology. Thus, imaging of A*β* plaques with non-invasive techniques including positron emission tomography (PET) and single-photon emission computed tomography (SPECT) now may aid in tracking amyloid pathology and achieving presymptomatic identification of AD patients^3^.

In recent decades, researchers have manipulated the Congo red (CR) and Thioflavin T (ThT) pharmacophores to make suitable imaging agents targeted to A*β* plaques. The result of this work is that several A*β*-selective PET imaging probes have been characterized clinically and demonstrated potential utility for the early diagnosis of AD. Most notably, [^18^F]**1**: AV-45 (florbetapir)^4^, [^18^F]**3**: GE-067 (flutemetamol)^5^, and [^18^F]**2**: BAY94-9172 (florbetaben)^6^ were approved by the U.S. FDA over the last two years (Fig. 1). However, as more SPECT scanners have been installed in hospitals, it would be more practical to design SPECT imaging agents. [^123^I]IMPY, an improved ThT derivative, has been well-characterized and tested in preclinical trials. The signal-to-noise ratio of this compound for A*β* labeling was found to be less than satisfactory, possibly due to its high lipophilicity and *in vivo* instability^7^,^8^.

Recently, we have transitioned away from these well-known A*β-*selective ligands that bear extended π-conjugated systems in rigid flat molecules. A class of ^125^I-labeled flexible benzyloxybenzene derivatives without highly rigid planarity have been synthesized and screened as radiotracers for SPECT imaging of A*β* plaques^9^. The most interesting compound ([^125^I]**4**) displayed strong labeling of A*β* plaques on brain sections from AD patients and transgenic (Tg) mice, good blood–brain barrier (BBB) penetration, rapid washout from the normal brain and satisfactory *in vitro* and *in vivo* biostability. In addition, two statistically reliable three-dimensional quantitative structure−activity relationship (3D-QSAR) models, comparative molecular field analysis (CoMFA) and comparative molecular similarity indices analysis (CoMSIA), were constructed to aid in the design of new potent A*β* imaging agents. Based on the benzyloxybenzene scaffold, we designed two ligands (**7a** and **12a**) with fluoroethoxy groups and iodides *para*-substituted at both benzene rings, which could be labeled with either ^18^F or ^125^I radioisotopes to give radioactive products with identical structures. These new ligands are expected to be both PET (^18^F) and SPECT (^123^I/^125^I) active, enabling both imaging modes using a single ligand (Fig. 1). These two ligands were separately ^18^F and ^125^I labeled and subjected to different *in vitro* and *in vivo* assays to assess their usefulness as PET/SPECT imaging agents targeted to A*β* plaques.

## Results

### Computational studies

**Figure 2:**
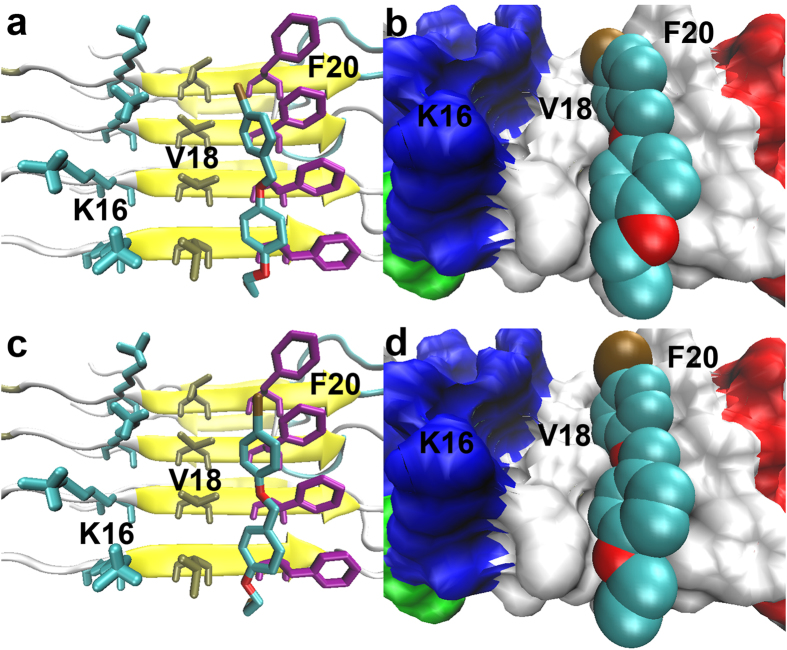
Lowest energy docked conformations of 7a (a,b) and 12a (c,d) packed against the hydrophobic Val18_Phe20 channel located on the flat surface of A*β* fibers (PDB ID: 2LMO). In the right-hand panels, the molecular surface of the A*β* fiber is represented with an opaque surface and the atomic radii of ligands with solid spheres to more vividly illustrate the tight binding of the ligands to the hydrophobic groove.

**Figure 3:**
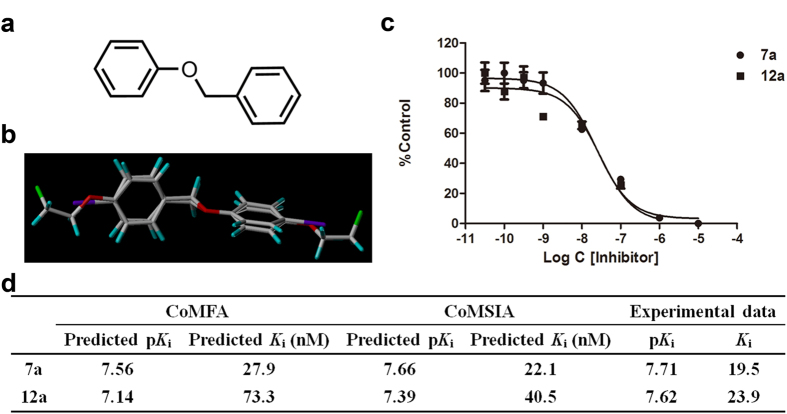
Prediction and experimental determination of the binding affinities of benzyloxybenzene derivatives 7a and 12a to A*β*_42_ aggregates. (**a**) Common core used for database alignment. (**b**) Superposition of molecules **7a** and **12a**. (**c**) Inhibition curves for the binding of [^125^I]**4** to A*β*_42_ aggregates. (**d**) Inhibition constants (*K*_i_) predicted by the QSAR models and actual *K*_i_ values obtained by the competition binding assay.

To evaluate the binding affinities of the newly designed dual imaging ligands to A*β* fibers before conducting synthetic and biological experiments, molecular docking and *K*_i_ predictions were performed^9^. Geometry optimizations were executed at the B3LYP/6–31G and 3-21G levels in the water phase. The optimized geometries of **7a** and **12a** were non-planar, resembling other recently reported benzyloxybenzene ligands (Figure S1a,b in Supplementary information). Similar results were obtained in the docking simulations. As shown in Fig. 2, ligands **7a** and **12a** were both predicted to insert into the hydrophobic Val18_Phe20 channel of A*β* fibers, the same as compound **4** and the well-studied IMPY molecule. To fit within the binding pocket, both ligands rotated into nearly flat conformations. **7a** and **12a** were predicted to bind tightly to A*β* fibers and compete well against compound **4** and IMPY. Previously constructed 3D-QSAR models successfully predicted the *K*_i_ values of **7a** and **12a**. As shown in Fig. 3d, the CoMFA and CoMSIA models predicted *K*_i_ values of 27.9 and 22.1 nM for **7a**, respectively, and 73.3 and 40.5 nM for **12a**, comparable or slightly inferior to the *K*_i_ values of IMPY and PIB (*K*_i_ = 32.2 nM and 38.8 nM)^9^ tested using [^125^I]**4** as the radio-ligand. These results imply that **7a** and **12a** may have sufficient binding to A*β* aggregates. Thus, we chemically synthesized and biologically assessed dual isotope labeled **7a** and **12a** for PET/SPECT imaging of A*β* plaques.

### Chemistry

**Figure 4:**
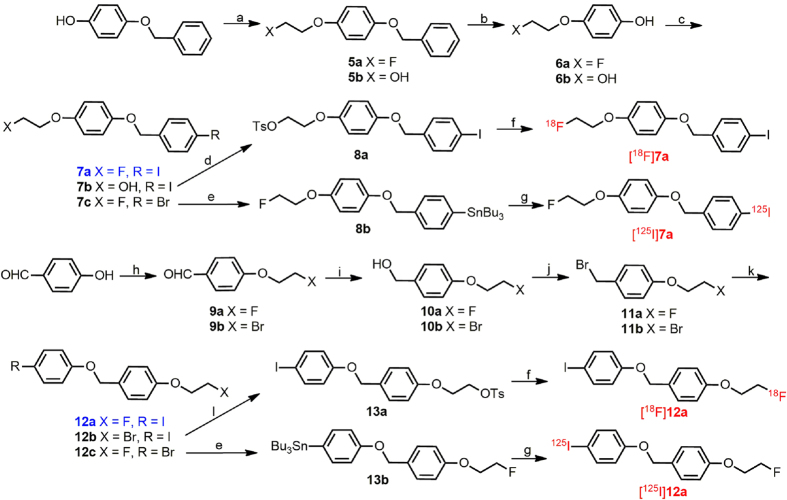
Chemical synthesis routes. Reagents and conditions: (**a**) 1-bromo-2-fluoroethane or 2-chloroethanol, KOH, EtOH, reflux; (**b**) 10% Pd/C, 1 atm H_2_, 50 °C; (**c**) 1-(bromomethyl)-4-iodobenzene or 1-bromo-4-(bromomethyl)benzene, K_2_CO_3_, DMF, 90 °C; (**d**) TsCl, CH_2_Cl_2_, Et_3_N, r.t.; (**e**) (Bu_3_Sn)_2_, (PPh_3_)_4_Pd, toluene, Et_3_N, reflux; (**f**)^18^F^–^, K_2_CO_3_, Kryptofix-2.2.2, acetonitrile, 100 °C; (**g**) [^125^I]NaI, HCl (1 M), H_2_O_2_ (3%); (**h**) 1-bromo-2-fluoroethane or 1,2-dibromoethane, K_2_CO_3_, DMF, 90 ^o^C; (**i**) NaBH_4_, MeOH, 0 °C; (**j**) PBr_3_, CH_2_Cl_2_, r.t.; (**k**) 4-iodophenol or 4-bromophenol, K_2_CO_3_, DMF, 90 °C; (**l**) AgOTs, MeCN, reflux.

Figure 4 outlines the synthesis of the desired benzyloxybenzene derivatives **7a**, **12a** and their precursors. The phenols **6a** and **6b** used to prepare **7a**-**7c** were acquired by coupling 1-bromo-2-fluoroethane or 2-chloroethanol with the hydroxy group of 4-(benzyloxy)phenol, followed by debenzylation with Pd/C under a hydrogen atmosphere. Coupling 1-bromo-2-fluoroethane or 1,2-dibromoethane with the hydroxy group of 4-hydroxybenzaldehyde yielded **9a** and **9b**. Then, **9a** and **9b** were reduced with NaBH_4_ to produce the corresponding benzyl alcohols **10a** and **10b** in high yields of 91.1% and 95.8%, respectively. Conversion of **10a** and **10b** to benzyl bromides **11a** and **11b** were achieved by bromination using PBr_3_ in high yields of 95.0% and 99.0%, respectively. The benzyloxybenzene backbone was formed in a base-catalyzed S_N_2 reaction between *para*-substituted benzyl bromides and corresponding phenols in yields greater than 70%. The tributyltin precursors **8b** and **13b** for ^125^I labeling were obtained using a bromo to tributyltin exchange reaction catalyzed by Pd(PPh_3_)_4_ from the corresponding bromo compounds **7c** and **12c** in yields of 30.4% and 25.1%, respectively. The tosylate precursor **8a** for ^18^F labeling was prepared through the common method of converting the free hydroxy group of **7b** into tosylate with TsCl in a yield of 40.6%. An attempt to synthesize the tosylate precursor **13a** from the hydroxyethoxy derivative 2-(4-((4-iodophenoxy)methyl)phenoxy) ethanol, failed due to the difficulty in obtaining the key intermediate, 2-(4-(bromomethyl)phenoxy)ethanol. Thus, an alternative method was adopted in which the bromo group was converted into tosylate using silver *p*-toluenesulfonate, providing the tosylate precursor **13a** from the bromoethoxy substituted compound **12b** in a yield of 57.0%.

Single crystals of **12a** were obtained by slow evaporation from ethyl acetate at room temperature, and its structure was then elucidated using the SHELXL97 program. The X-ray crystallographic structure conformed well to the geometries of the optimized structure with a root mean square deviation (RMSD) of 0.19 Å, which was calculated using VMD 1.9.1 software after alignment (Figure S1 in Supplementary information). The small RMSD value suggested that the geometric optimizations were successful. Therefore, it is valid to use the optimized structures for the QSAR and molecular docking calculations.

### *In vitro* binding assay using A*β* aggregates

The binding affinities of **7a** and **12a** to A*β*_42_ aggregates were quantitatively evaluated by a competition binding assay using [^125^I]**4** as the competing radio-ligand according to conventional methods^10^. Ligands **7a** and **12a** inhibited the binding of [^125^I]**4** with *K*_i_ values of 19.5 ± 7.1 nM and 23.9 ± 7.9 nM, respectively, which were slightly better than that of IMPY (*K*_i_ = 32.2 ± 2.1 nM) and PIB (*K*_i_ = 38.8 ± 2.6 nM) in the identical assay (Fig. 3). These values suggest that **7a** and **12a** have excellent binding abilities and can efficiently label A*β* plaques in the AD brain. The actual binding affinities agreed well with values predicted by the CoMFA and CoMSIA models, with p*K*_i_ values deviating by less than 0.5 log unit. This satisfactory result strongly validated the reliability of the established 3D-QSAR models. This ability to accurately predict the binding activities of new benzyloxybenzene derivatives will be of great help for future efforts to design new potent A*β* imaging agents.

### Radiolabeling

^125^I was introduced using corresponding tributyltin precursors via an iododestannylation reaction, providing [^125^I]**7a** and [^125^I]**12a** in high radiochemical yields of 67% and 93%, respectively. The specific activity of the no-carrier-added radioiodinated products was anticipated to be similar to that of [^125^I]NaI (2200 Ci/mmol). ^18^F labeling was achieved using tosylates **8a** and **13a** as precursors for nucleophilic substitution with [^18^F]fluoride, offering [^18^F]**7a** and [^18^F]**12a** in moderate radiochemical yields of 24–37% with a specific activity approximate to 60 GBq/*μ*mol (decay corrected). Simple purification using HPLC yielded these ^125^I- and ^18^F-labeled radiotracers in a radiochemical purity of greater than 98%. Small deviations of retention times between these radiotracers and corresponding non-radioactive compounds in co-injection HPLC analysis successfully verified their radiochemical identities (Table S2, Figure S2 in Supplementary information).

### *In vitro* autoradiography

**Figure 5:**
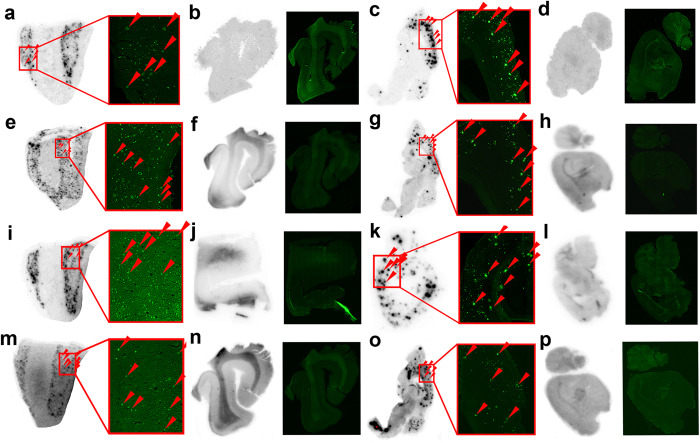
*In vitro* autoradiography of [^125^I]7a (a–d), [^18^F]7a (e–h), [^125^I]12a (i–l) and [^18^F]12a (m–p). (**a,e,i** and **m**) AD human brain sections, female, 64 years old; (**b,f,j** and **n**) normal human brain sections, male, 74 years old; (**c,g,k** and **o**) Tg mouse brain sections, APPswe/PSEN1, 11 months old; (**d,h,l** and **k**) wild-type mouse brain sections, C57BL6, 11 months old. Fluorescence staining using ThS on the right panel of each image confirmed the location and distribution of plaques on the same sections.

To characterize the high specific binding abilities of these dual radiolabeled imaging agents to A*β* plaques, *in vitro* autoradiography was performed on brain sections from AD patients and Tg model mice. As shown in Fig. 5a,e,i,m,c,g,k,o, clusters of hot spots accumulated in the cerebral cortex sections of AD patient and Tg mouse brains, and the locations coincided precisely with the fluorescent specks observed after staining with Thioflavin-S (ThS), a conventional dye usually used to stain A*β* plaques. In contrast, healthy human and wild-type mouse brain sections displayed no significant accumulation of radioactivity (Fig. 5b,f,j,n,d,h,l,p). This result reflected the high specific binding of **7a** and **12a** in the competition binding assay, and proved the feasibility and practicality of using [^125^I]**7a**, [^18^F]**7a**, [^125^I]**12a** and [^18^F]**12a** to specifically label A*β* deposits in AD brains.

### *In vivo* studies

An ideal A*β* imaging probe would combine a high binding affinity to A*β* plaques and excellent pharmacokinetics with high initial brain uptake and rapid washout from normal brain regions. Thus, the partition coefficients of these four radiolabeled ligands were determined to estimate their ability to penetrate the BBB. The log *D* values ([^125^I]**7a**: 3.96 ± 0.22; [^18^F]**7a**: 3.88 ± 0.17; [^125^I]**12a**: 3.62 ± 0.15; [^18^F]**12a**: 3.84 ± 0.07) shown in Table S3 in Supplementary information implied that the four radiolabeled tracers have slightly higher lipophilicity but are still able to cross the BBB.

**Figure 6:**
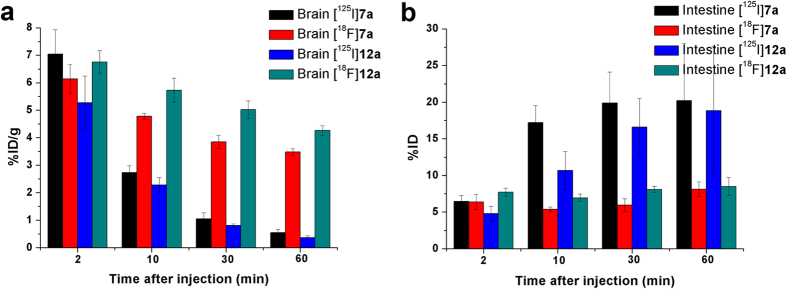
Plots of brain and intestinal uptake of [^125^I]7a, [^18^F]7a, [^125^I]12a and [^18^F]12a in normal ICR mice at different post-injection time points.

Histograms of brain and intestine uptake were presented in Fig. 6 for comparison, while detailed data concerning the *in vivo* biodistribution in normal ICR mice were collected in Table S3 in Supplementary information. All four radiolabeled tracers exhibited high initial brain uptake at 2 min post-injection (>5% ID/g). [^125^I]**7a** and [^125^I]**12a** were rapidly cleared from the normal brain, with only small amounts of radioactivity remaining at 60 min post-injection (0.55% ID/g for [^125^I]**7a** and 0.37% ID/g for [^125^I]**12a**). The brain_2_ _min_/brain_60_ _min_ ratio has generally been used as an important index to evaluate clearance, and the values of this ratio for [^125^I]**7a** and [^125^I]**12a** were as high as 12.8 and 14.2, respectively. The liver is significant for drug metabolism and clearance, resulting in a maximum tracer concentration in the liver of approximately 20% ID/g at 2 min, which gradually decreased to 5% ID/g at 60 min. In addition, no significant deiodination was observed (less than 0.5% ID in the thyroid at 60 min). The pharmacokinetics were favorable, comparable to those of [^125^I]**4** and [^125^I]IMPY^7^, and superior to those of ^125^I-labeled benzimidazole^11^, benzofuran^12^,^13^, quinoxaline^14^, quinacrine^15^, 1,3,4-oxadiazole^16^, flavone^17^, aurone^18^ and chalcone derivatives^19^, suggesting that [^125^I]**7a** and [^125^I]**12a** are suitable for SPECT imaging of A*β* plaques in the AD brain.

The clearance properties of [^18^F]**7a** and [^18^F]**12a** differed from those of the corresponding ^125^I-labeled ligands. Substantial amounts of radioactivity were detected in the brain until 60 min post-injection (3.48% ID/g for [^18^F]**7a** and 4.26% ID/g for [^18^F]**12a**), resulting in brain_2_ _min_/brain_60_ _min_ ratios as low as 1.8 and 1.6, respectively. Meanwhile, radioactivity in the blood, heart, spleen and muscle all decreased slowly over time, and the uptake levels remained appreciable at 60 min post-injection. By closely comparing the initial uptakes, we found that less [^18^F]**7a** and [^18^F]**12a** accumulated in the liver for metabolism and elimination in the early stages, leaving more radioactive tracers in the blood and muscle. This was probably one factor contributing to the slow clearance of these compounds. Examining the intestinal uptake, we found continuous intestinal accumulation of radioactivity for [^125^I]**7a** and [^125^I]**12a**, with the levels reaching approximately 20% ID/g at 60 min post-injection. In contrast, [^18^F]**7a** and [^18^F]**12a** exhibited no significant concentration in the intestine, maintaining nearly constant radioactivity values of approximately 8% ID/g through the whole investigative time period. Biodistribution studies of ^18^F-labeled *7a* and **12a** implied that the majority of radioactive compounds were not excreted by the normal mice and remained in circulation, resulting in relatively slower washout rates from the brain.

**Figure 7:**
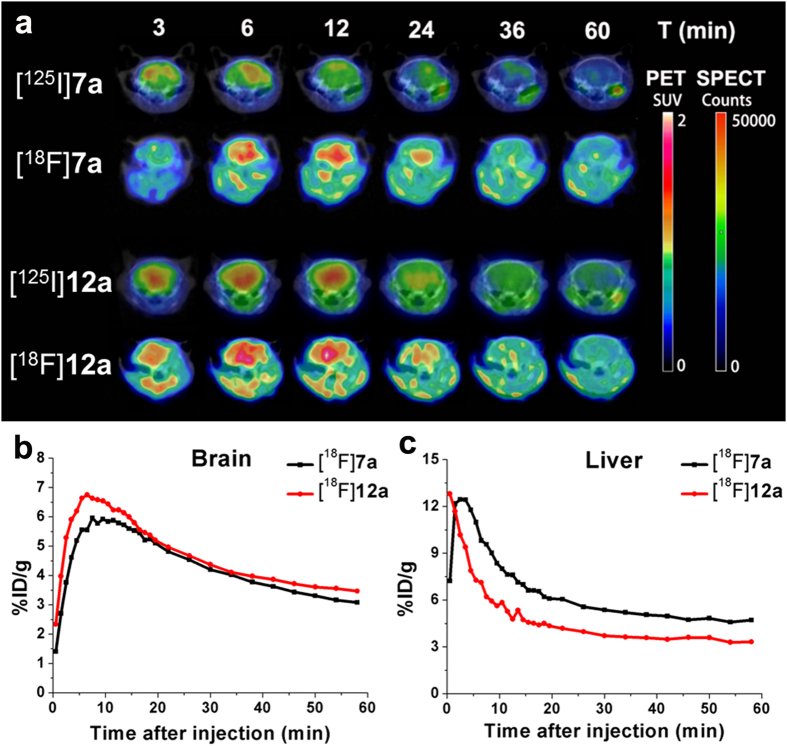
Dynamic microPET/CT and microSPECT/CT imaging. (**a**) Transversal brain SPECT images of [^125^I]**7a**, [^125^I]**12a** and PET images of [^18^F]**7a**, [^18^F]**12a** superimposed onto CT templates. SPECT image color intensities are expressed as counts, while PET images represent SUV. Upper row indicates time points of image acquisition. (**b,c**) TACs of [^18^F]**7a** and [^18^F]**12a** in brain and liver for the entire 60 min PET scan.

To further assess the pharmacokinetics of these two couple of radiolabeled benzyloxybenzenes, following dynamic PET/CT and SPECT/CT scans were conducted in normal ICR mice. High contrast PET and SPECT images implied that all the four radiolabeled benzyloxybenzenes can penetrate the BBB to a great degree and washout from the normal brain regions (Fig. 7a). Whole-body PET images of [^18^F]**7a** and [^18^F]**12a** showed that the radioactivity mainly accumulated in the brain, heart and liver at the early stages (Figure S3 in Supplementary information). Sustained low muscle and bone uptakes were observed during the entire 60 min scans with standardized uptake values (SUV) of approximate 0.7. As shown in Fig. 7b, the time−activity curves (TACs) derived from dynamic PET imaging data of [^18^F]**7a** and [^18^F]**12a** evidenced excellent brain uptake ([^18^F]**7a**: 5.96% ID/g at 8 min; [^18^F]**12a**: 6.74% ID/g at 7 min) and moderate clearance profiles with high radioactivity retention at 60 min ([^18^F]**7a**: 3.08% ID/g; [^18^F]**12a**: 3.47% ID/g). These PET imaging data well matched the *in vivo* biodistribution results.

As the specific activity of ^18^F-labeled ligands (about 60 GBq/*μ*mol) was slightly lower than that of corresponding no-carrier-added ^125^I-labeled ligands (81 GBq/*μ*mol), the impact of specific activity on pharmacokinetics was studied. *In vivo* biodistribution of radioactivity after injection of carrier-added [^125^I]**7a** with equal specific activity to corresponding [^18^F]**7a** (60 GBq/*μ*mol) was conducted in normal ICR mice through the same procedure. As shown in Table S3 in Supplementary information, the brain uptake and clearance profiles (5.39% ID/g at 2 min and 0.37% ID/g at 60 min) were similar to that of the no-carrier-added [^125^I]**7a** (7.04% ID/g at 2 min and 0.55% ID/g at 60 min). The brain_2_ _min_/brain_60_ _min_ ratio of 14.2 was comparable to that of no-carrier-added [^125^I]**7a** (12.8) and much higher than that of [^18^F]**7a** (1.8). This result demonstrated that the specific activity was not responsible for the slow clearance of ^18^F-labeled benzyloxybenzenes.

Structurally identical ^125^I/^18^F-labeled aurone and ^11^C/^18^F-labeled chalcone derivatives previously reported by Ono *et al.* showed a similar difference in the *in vivo* pharmacokinetics of radioactive components, which the authors attributed to differences in the physicochemical characteristics of the radiometabolites produced in the brain^18^,^20^. However, no further investigation was conducted to identify the precise reason for this difference.

To address this question, the *in vivo* metabolic studies of [^125^I]**7a**, [^18^F]**7a**, [^125^I]**12a** and [^18^F]**12a** in normal ICR mice were evaluated by HPLC analysis of selected organ samples obtained at different post-injection time points (Table S4, Table S5 and Figure S4–S7 in Supplementary information). All four radioactive ligands degraded to two corresponding polar radioactive products with shorter retention times. [^125^I]**7a** and [^125^I]**12a** both displayed excellent stability in the brain, with 95.9% and 92.1% of the radioactivity present as parent tracers at 2 min and 72.1% and 68.6% after 60 min, respectively. The good *in vivo* biostability of these compounds enhances their potential for SPECT mapping of the brain. [^18^F]**7a** and [^18^F]**12a** degraded to a greater extent in the brain, with only 34.0% and 29.8% intact at 60 min. In the plasma, all four ligands were rapidly converted to polar metabolites, leaving only approximately 10% of the parent tracers at 60 min. In the liver, the main metabolic organ, no significant metabolism of [^18^F]**7a** and [^18^F]**12a** occurred, and the levels of intact [^18^F]**7a** and [^18^F]**12a** remained as high as 48.7% and 82.2% after 30 min. In contrast, the breakdown of [^125^I]**7a** and [^125^I]**12a** was so rapid that almost negligible amounts of parent tracers remained at 30 min (8.8% and 6.3%, respectively). The polar and hydrophilic metabolites metabolized by the liver could be absorbed by the intestine and excreted through the urine, in accordance with the biodistribution results. The rapid metabolism in the liver may be another reason for the faster clearance of these compounds from the normal brain. For structurally identical molecules labeled with radioactive isotopes at different positions, the *in vivo* metabolic pathways were expected to be the same. However, as only radioactive products can be detected with PET/SPECT scanners, different pharmacokinetics of the radioactive components could lead to different imaging results. This dual radiolabeling strategy has been widely used to demonstrate the proposed mechanism of action of drugs^21^. The dual radiolabeled identical benzyloxybenzene derivatives in this study were expected to undergo the same metabolic reactions, generating different radioactive metabolites. Differences in the biochemical characteristics of the radiometabolites including absorption, distribution, metabolism and excretion are responsible for the discrepancy in the pharmacokinetics of the radioactivity.

**Figure 8:**
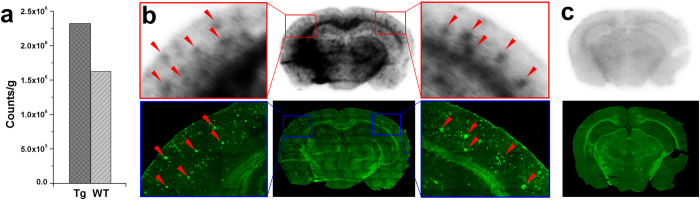
*Ex vivio* autoradiography of [^125^I]7a. (**a**) A comparison of brain radioactivity retention after intravenous injection of [^125^I]**7a** into a transgenic mouse (APPswe/PSEN1, male, 27 months old) and a wild-type mouse (C57BL6, male, 27 months old). (**b,c**) Ex vivo autoradiography of [^125^I]**7a**. The A*β* plaques were confirmed by *in vitro* staining of the same brain sections with ThS.

To further characterize the *in vivo* A*β* imaging capabilities, we conducted *ex vivo* autoradiographic studies with [^125^I]**7a** which combined high binding affinity, high initial brain uptake, fast clearance and excellent *in vivo* biostability in the brain. [^125^I]**7a** (1 mCi, 200 *μ*L) was injected intravenously into a Tg mouse (APPswe/PSEN1, male, 27 months old) and an age-matched wild-type mouse (C57BL6, male). Thirty minutes after injection, the brains were removed, weighed and the radioactivity was counted. Then the brains were frozen and sectioned for ex vivo autoradiography. As shown in Fig. 8, Tg mouse brain exhibited 40% higher radioactivity retention than that of wild-type control mouse at 30 min post-injection. Autoradiographic images displayed clear labeling of A*β* plaques in the Tg mouse brain, and the distributions of radioactive spots correlated well with the amyloid burden as visualized in the same brain sections stained with ThS (Fig. 8b). Conversely, no such labeling was observed in wild-type mouse brain (Fig. 8c). These results suggested that [^125^I]**7a** penetrated the intact BBB and bound to cerebral A*β* plaques specifically *in vivo*.

## Discussion

Two structurally identical fluorinated and iodinated benzyloxybenzene derivatives were designed for PET/SPECT imaging of A*β* plaques. Molecular docking and 3D-QSAR models predicted excellent binding to A*β* fibers. The high binding affinities were verified by an *in vitro* competition binding assay using A*β* aggregates with *K*_i_ values of 19.5 nM and 23.9 nM for **7a** and **12a**, respectively. Then, the compounds were separately ^18^F and ^125^I labeled in high radiochemical yields that were suitable for commercial scale production. *In vitro* autoradiography showed that all of the radiolabeled ligands specifically labeled A*β* plaques on brain sections from AD patients and Tg mice, reflecting the high binding affinities. Biodistribution experiments showed that [^125^I]**7a** and [^125^I]**12a** exhibited high initial uptakes (7.04 and 5.27% ID/g at 2 min) and rapid washout, with brain_2_ _min_/brain_60_ _min_ ratios of 12.8 and 14.2, respectively. [^18^F]**7a** and [^18^F]**12a** also displayed high initial uptake but moderate clearance. Dynamic microPET/CT and microSPECT/CT imaging in normal ICR mice generated similar results. All the four radiotracers exhibited high BBB penetration abilities, and [^18^F]**7a** and [^18^F]**12a** showed moderate egress from the normal brain. In metabolic studies, ^125^I-labeled benzyloxybenzene derivatives displayed good biostability in the brain, while the corresponding ^18^F-labeled tracers exhibited slightly severer degradation to polar radiometbaolites. The metabolic studies explained the discrepancy of the *in vivo* pharmacokinetics of the structurally identical ^125^I- and ^18^F-labeled tracers. After injected intravenously into a Tg mouse and a wild-type mouse, [^125^I]**7a** was retained *in vivo* in amyloid-laden brain and, in contrast, was rapidly washed out from healthy brain. Ex vivo autoradiography demonstrated that [^125^I]**7a** did penetrate the BBB and bind strongly to A*β* plaques *in vivo*. Overall, the preliminary results suggested that the ^125^I-labeled benzyloxybenzenes are excellent candidates as SPECT probes for the early detection of A*β* plaques in AD brains. In pursuit of new ^18^F-labeled benzyloxybenzenes with improved clearance property and *in vivo* biostability, additional chemical refinements are under study.

## Methods

### General Remarks

A structural model of an A*β* fiber (PDB ID: 2LMO) derived from solid state NMR was downloaded from the RCSB Protein Data Bank (www.rcsb.org/pdb). Geometric optimization was performed using Gaussian 09 at the B3LYP/6–31 G and 3-21G levels in the water phase. Molecular docking was performed using AutoDock4.0, and the docking results were graphed with VMD 1.9.1 software. SYBYL-X 1.1 software was used to predict the binding affinities with previously constructed 3D-QSAR models. All reagents for chemical synthesis were used as purchased without further purification. [^125^I]NaI was purchased from PerkinElmer and ^18^F^–^ was kindly provided by the Chinese PLA General Hospital. ^1^H and ^13^C NMR spectra were recorded on a Bruker Avance III NMR spectrometer (400 MHz for ^1^H; 101 MHz for ^13^C) in CDCl_3_ or DMSO-*d*_6_ solutions at room temperature. Mass spectra were acquired with a GCT CA127 Micronass UK instrument. X-ray crystallographic data of compound **12a** were collected on a Bruker Smart APEX II diffractometer (Bruker Co., Germany) and deposited at the Cambridge Crystallographic Data Centre as supplementary publication (no. CCDC 1063679). HPLC analysis was performed on a Shimadzu SCL-20 AVP equipped with a Bioscan Flow Count 3200 NaI/PMT *γ*-radiation scintillation detector and a SPD-20A UV detector, *λ* = 254 nm. A Venusil MP C18 reverse phase column (Agela Technologies, 5 *μ*m, 4.6 × 250 mm) was used for separations and purity determinations with a binary gradient system (acetonitrile: water = 80%: 20%) at a 1.0 mL/min flow rate. Fluorescent observation was performed on the Axio Observer Z1 inverted fluorescence microscope (Zeiss, Germany) equipped with a DAPI filter set (excitation, 405 nm). Post-mortem brain sections from an autopsy-confirmed AD subject (64 years old, female) and a control subject (74 years old, male) were acquired from the Chinese Brain Bank. Transgenic mice (APPswe/PSEN1, male) and wild-type mice (C57BL6, male) were purchased from the Institute of Laboratory Animal Science, Chinese Academy of Medical Sciences. Normal ICR mice (20–22 g, male) were used for *in vivo* biodistribution, dynamic PET/CT and SPECT/CT scans, and biostability experiments. All experiments on mice were performed in accordance with the guidelines approved by the animal care committee of Beijing Normal University and Xiamen University.

### Computational methods

To guarantee the reliability of the docking results and the predicted *K*_i_ values, all of the molecules were prepared in the same manner as the other benzyloxybenzene derivatives we recently reported. Briefly, molecules **7a** and **12a** were constructed with GaussView 5.0 based on the X-ray crystal structural of **4** (1-iodo-4-((4-methoxyphenoxy)methyl)benzene). Geometric optimizations were performed in Gaussian 09 (Gaussian 09, Revision C.01, Gaussian, Inc., Wallingford CT, 2010) using B3LYP/3-21G^22^,^23^,^24^ for iodine and B3LYP/6-31G^25^ for other atoms in the water phase. A lamarckian genetic algorithm^26^ was used to perform docking simulations on A*β* fibers (PDB ID: 2LMO^27^) with AutoDock4.0^28^,^29^ according to previously described methods. SYBYL-X 1.1 software (Sybyl-X, version 1.1, Tripos Inc., St. Louis, MO, 2010) was used to predict the *K*_i_ values of **7a** and **12a** with the previously generated CoMFA and CoMSIA models. As ligand **4** was the template molecule that everything was aligned to in the preceding calculations, it was added to a new database along with **7a** and **12a**. **7a** and **12a** were charged and aligned in the same way as the molecules in these models. A spreadsheet was created from the new database, and the *K*_i_ values were predicted using the previously developed CoMFA and CoMSIA models as the tables referencing the analysis.

### Radiolabeling and Biological Evaluation

The radio-iodinated and fluorinated ligands **7a** and **12a** were prepared from the corresponding tributyltin and tosylate precursors using previously reported procedures^30^.

Biological evaluation including the *in vitro* binding assay using A*β* aggregates, *in vitro* autoradiography, partition coefficient determination, biodistribution and *in vivo* biostability studies were all conducted according to previously reported methods^9^,^10^.

Four normal ICR mice (20–22 g, male) were used for dynamic microPET/CT and microSPECT/CT imaging. The whole-body PET/CT scans were performed using Siemens Inveon device (Siemens Medical Solutions), while SPECT/CT scans were conducted with Mediso nanoScan SPECT/CT scanner (Mediso Medical Imaging System). All mice were anesthetized with 2% isoflurane. Dynamic PET and SPECT scans from 0 to 60 min were started immediately after intravenous injection of [^18^F]**7a** (injected dose, 2.2 MBq), [^18^F]**12a** (injected dose, 4.1 MBq), [^125^I]**7a** (injected dose, 25 MBq), and [^125^I]**12a** (injected dose, 48 MBq), respectively. PET images were reconstructed using three-dimensional ordered subsets expectation maximization (3D OSEM) algorithm, and 3D iterative algorithm was used for SPECT image reconstruction. Time frames for PET imaging were 20 × 60 s and 10 × 240 s, and that for SPECT imaging were 20 × 180 s. Regions of interests (ROIs) were manually drawn on the whole brain, heart, liver, muscle and bone using the CT templates and then transferred to corresponding coregistered PET images. SUVs, injected dose and body weight normalized and decay corrected radioactivity concentration, were calculated for all ROIs. TACs were calculated from this.

Ex vivo autoradiography was performed on a transgenic mouse (APPswe/PSEN1, male, 27 months old) and a wild-type mouse (C57BL6, male, 27 months old). A saline solution (200 *μ*L, 10% EtOH) of 1 mCi of [^125^I]**7a** was injected into the mice via the tail vein. The mice were sacrificed by decapitation at 30 min post-injection, and the brains were immediately removed, weighed and the radioactivity was counted. Frozen brain sections of 20 *μ*m were cut and exposed to a phosphorus plate (Perkin-Elmer, USA) for one week. Ex vivo autoradiographic images were obtained using a phosphor imaging system (Cyclone, Packard). Subsequently the same sections were stained with ThS to confirm the presence and location of A*β* plaques.

## Additional Information

**How to cite this article**: Yang, Y. *et al.* Preliminary Characterization and *In Vivo* Studies of Structurally Identical ^18^F- and ^125^I-Labeled Benzyloxybenzenes for PET/SPECT Imaging of *β*-Amyloid Plaques. *Sci. Rep.* **5**, 12084; doi: 10.1038/srep12084 (2015).

## Supplementary Material

Supplementary Information

## Figures and Tables

**Figure 1:**
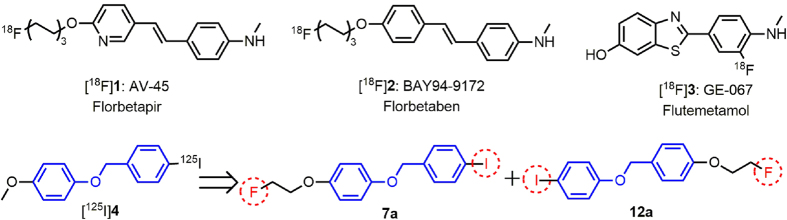
A*β* probes approved by the U.S. FDA, and the design of structurally identical ^18^F- and ^125^I-labeled benzyloxybenzene derivatives for PET/SPECT imaging of A*β* plaques.
