# Supplementary material for: Preliminary Characterization and In Vivo Studies of Structurally Identical 18F- and 125I-Labeled Benzyloxybenzenes for PET/SPECT Imaging of β-Amyloid Plaques
Source: Sci Rep. 2015 Jul 14;5:12084. doi: 10.1038/srep12084 (PMC4501006; doi:10.1038/srep12084)
Supplement: Supplementary Information [file srep12084-s1.pdf]

SUPPLEMENTARY INFORMATION

Preliminary Characterization and In Vivo Studies of Structurally Identical ^{18}F- and ^{125}I-Labeled Benzyloxybenzenes for PET/SPECT Imaging of *β*-Amyloid Plaques

Yanping Yang[1], Xiaoyang Zhang[1], Mengchao Cui[1,*], Jinming Zhang[2,*], Zhide Guo[3], Yesen Li[3], Xianzhong Zhang[3], Jiapei Dai[4] & Boli Liu[1]

[1]Key Laboratory of Radiopharmaceuticals, Ministry of Education, College of Chemistry, Beijing Normal University, Beijing 100875, P. R. China, [2]Department of Nuclear Medicine, Chinese PLA General Hospital, Beijing 100853, P. R. China, [3]Center for Molecular Imaging and Translational Medicine, State Key Laboratory of Molecular Vaccinology and Molecular Diagnostics, School of Public Health, Xiamen University, Xiamen 361102, China, [4]Wuhan Institute for Neuroscience and Neuroengineering, South-Central University for Nationalities, Wuhan 430074, P. R. China.

Corresponding Author:

*Tel/Fax: +86-10-58808891. E-mail: cmc@bnu.edu.cn (M. Cui)

*Tel/Fax: +86-10-66936108. E-mail: zhangjm301@yahoo.com.cn (J. Zhang)

Table of Contents

1 Chemical Synthesis

2 Supplementary Tables

2.1 Table S1. Crystal data and structure refinements for compound **12a**

2.2 Table S2. Purity of key target compounds

2.3 Table S3. Biodistribution of [^{125}I]**7a**, [^{18}F]**7a**, [^{125}I]**12a** and [^{18}F] **12a** in normal ICR mice

2.4 Table S4. Percentages of metabolites after intravenous injection of [^{125}I]**7a** and [^{18}F]**7a** in to ICR mice

2.5 Table S5. Percentages of metabolites after intravenous injection of [^{125}I]**12a** and [^{18}F]**12a** in to ICR mice

3 Supplementary Figures

3.1 Figure S1. Optimized structures of **7a**, **12a** and X-ray crystal structure of **12a**

3.2 Figure S2. Co-injection HPLC profiles of **7a** and [^{125}I]**7a**; **7a** and [^{18}F]**7a**; **12a** and [^{125}I]**12a**; **12a** and [^{18}F]**12a**

3.3 Figure S3. Whole-body dynamic microPET/CT imaging of [^{18}F]**7a** and [^{18}F]**12a** in normal ICR mice.

3.4 Figure S4. HPLC profiles for radioactive metabolites of [^{125}I]**7a** in ICR mouse organs

3.5 Figure S5. HPLC profiles for radioactive metabolites of [^{18}F]**7a** in ICR mouse organs

3.6 Figure S6. HPLC profiles for radioactive metabolites of [^{125}I]**12a** in ICR mouse organs

3.7 Figure S7. HPLC profiles for radioactive metabolites of [^{18}F]**12a** in ICR mouse organs

4 NMR and MS Spectra

Chemical Synthesis

1-(Benzyloxy)-4-(2-fluoroethoxy)benzene (5a)

A mixture of 4-(benzyloxy)phenol (2.00 g, 10.0 mmol) and KOH (0.56 g, 10.0 mmol) in dry EtOH (30 mL) was stirred under reflux for 30 min. 1-bromo-2-fluoroethane (1.52 g, 12.0mmol) was then added dropwise, and the mixture was further stirred for 1 h, and evaporated under a vacuum. A white precipitate was formed by adding 50 mL of 1M NaOH, which was then filtered, washed with 50 mL water and recrystallized from methanol to obtain a white solid of **5a** (2.23 g, 90.4%). mp: 69.1-69.6 ℃; ^{1}H NMR (400 MHz, $CDCl_3$) δ 7.46 – 7.28 (m, 5H), 6.91 (d, *J* = 9.2 Hz, 2H), 6.86 (d, *J* = 9.3 Hz, 2H), 5.02 (s, 2H), 4.81 – 4.64 (m, 2H), 4.22 – 4.10 (m, 2H); MS (EI): m/z calcd for $C_{15}H_{15}FO_2$ 246; found 246 M^+.

2-(4-(Benzyloxy)phenoxy)ethanol (5b)

The procedure described above for the preparation of **5a** was employed to obtain a white solid of **5b** from 4-(benzyloxy)phenol and 2-chloroethanol (2.35 g, 48.1%). mp: 105.8-106.4 ℃; ^{1}H NMR (400 MHz, $CDCl_3$) δ 7.46 – 7.30 (m, 5H), 6.91 (d, *J* = 9.1 Hz, 2H), 6.85 (d, *J* = 9.0 Hz, 2H), 5.02 (s, 2H), 4.08 – 3.99 (m, 2H), 3.97 – 3.87 (m, 2H), 1.95 (s, 1H); MS (EI): m/z calcd for $C_{15}H_{16}O_3$ 244; found 244 M^+.

4-(2-Fluoroethoxy)phenol (6a)

To a solution of **5a** (2.08 g, 8.44 mmol) in anhydrous MeOH (10 mL) was added 10% Pd/C (89.4 mg, 0.84 mmol). The mixture was stirred for 4 h at 50 ℃ under 1 atm of hydrogen atmosphere. The catalyst was filtered while hot and washed with MeOH, and the filtrate was concentrated under reduced pressure to give a white solid of **6a** (1.32 g, 57.4%). mp: 95.7-96.5 ℃; ^{1}H NMR (400 MHz, $CDCl_3$) δ 6.83 (d, *J* = 8.9 Hz, 2H), 6.77 (d, *J* = 9.0 Hz,

2H), 4.80 – 4.64 (m, 2H), 4.42 (s, 1H), 4.23 – 4.09 (m, 2H); MS (EI): m/z calcd for $C_8H_9FO_2$ 156; found 156 M^+.

4-(2-Hydroxyethoxy)phenol (6b)

The procedure described above for the preparation of **6a** was employed to obtain a white solid of **6b** from **5b** (1.35 g, 100%). mp: 92.1-92.8 ℃; ^{1}H NMR (400 MHz, DMSO-d_6) δ 8.86 (s, 1H), 6.74 (d, *J* = 8.6 Hz, 2H), 6.66 (d, *J* = 8.8 Hz, 2H), 4.78 (t, *J* = 5.2 Hz, 1H), 3.90 – 3.80 (m, 2H), 3.70 – 3.61 (m, 2H); MS (EI): m/z calcd for $C_8H_{10}O_3$ 154; found 154 M^+.

1-(2-Fluoroethoxy)-4-((4-iodobenzyl)oxy)benzene (7a)

To a solution of **6a** (468.5 mg, 3.0 mmol) and 1-(bromomethyl)-4-iodobenzene (890.8 mg, 3.0 mmol) in anhydrous DMF (5 mL), K_2CO_3 was added (414.6 mg, 3.0 mmol). The resulting mixture was stirred at 90 ℃ for 30 min. After cooling to room temperature, a white precipitate was formed by adding 50 mL of water, which was then filtered, washed with 50 mL of water and recrystallized from methanol to obtain a white solid of **7a** (986.8 mg, 88.4%). mp: 108.3-108.8 ℃; ^{1}H NMR (400 MHz, $CDCl_3$) δ 7.70 (d, *J* = 8.3 Hz, 2H), 7.16 (d, *J* = 8.2 Hz, 2H), 6.90 – 6.84 (m, 4H), 4.96 (s, 2H), 4.79 – 4.65 (m, 2H), 4.21 – 4.11 (m, 2H); ^{13}C NMR (101 MHz, $CDCl_3$) δ 153.11 (C_q), 152.93 (C_q), 137.62 (2 × CH), 136.94 (C_q), 129.24 (2 × CH), 115.89 (2 × CH), 115.79 (2 × CH), 93.35 (C_q), 82.01 (d, *J* = 170.6 Hz, CH_2), 69.96 (CH_2), 67.87 (d, *J* = 20.5 Hz, CH_2); HRMS (EI): m/z calcd for $C_{15}H_{14}FIO_2$ 372.0023; found 372.0029 M^+.

2-(4-((4-Iodobenzyl)oxy)phenoxy)ethanol (7b)

The procedure described above for the preparation of **7a** was employed to obtain a white solid of **7b** from **6b** and 1-(bromomethyl)-4-iodobenzene (1.38 g, 72.9%). mp: 129.3-129.9

℃; ^{1}H NMR (400 MHz, $CDCl_3$) δ 7.70 (d, *J* = 8.3 Hz, 2H), 7.17 (d, *J* = 8.4 Hz, 2H), 6.89 – 6.83 (m, 4H), 4.96 (s, 2H), 4.06 – 4.00 (m, 2H), 3.96 – 3.91 (m, 2H); MS (EI): m/z calcd for $C_{15}H_{15}IO_3$ 370; found 370 M^+.

1-Bromo-4-((4-(2-fluoroethoxy)phenoxy)methyl)benzene (7c)

The procedure described above for the preparation of **7a** was employed to obtain a white solid of **7c** from **6a** and 1-bromo-4-(bromomethyl)benzene (1.55 g, 95.2%). mp: 115.6-116.4 ℃; ^{1}H NMR (400 MHz, $CDCl_3$) δ 7.50 (d, *J* = 8.4 Hz, 2H), 7.29 (d, *J* = 8.4 Hz, 2H), 6.90 – 6.84 (m, 4H), 4.97 (s, 2H), 4.80 – 4.65 (m, 2H), 4.21 – 4.11 (m, 2H); MS (EI): m/z calcd for $C_{15}H_{14}BrFO_2$ 324; found 324 M^+.

2-(4-((4-Iodobenzyl)oxy)phenoxy)ethyl 4-methylbenzenesulfonate (8a)

A mixture of **7b** (740.4 mg, 2.0 mmol) and Et_3N (10 mL) was stirred in anhydrous CH_2Cl_2 (10 mL) in an ice bath, and tosyl chloride (571.9 mg, 3.0 mmol) was slowly added. The reaction mixture was stirred for 4 h at room temperature, and the solvent was evaporated under reduced pressure. Water was added (50 mL), and the mixture was extracted by CH_2Cl_2 (3 × 10 mL). Combined organic layers were dried over anhydrous $MgSO_4$, filtered and concentrated under a vacuum. The crude mixture was purified by silica gel chromatography (petroleum ether/AcOEt = 4/1, v/v) to obtain a white solid of **7a** (425.7 mg, 40.6%). mp: 120.8-121.5 ℃; ^{1}H NMR (400 MHz, $CDCl_3$) δ 7.81 (d, *J* = 8.3 Hz, 2H), 7.70 (d, *J* = 8.3 Hz, 2H), 7.33 (d, *J* = 8.1 Hz, 2H), 7.15 (d, *J* = 8.3 Hz, 2H), 6.83 (d, *J* = 9.1 Hz, 2H), 6.72 (d, *J* = 9.1 Hz, 2H), 4.94 (s, 2H), 4.36 – 4.31 (m, 2H), 4.12 – 4.07 (m, 2H), 2.44 (s, 3H); MS (EI): m/z calcd for $C_{22}H_{21}IO_5S$ 524; found 524 M^+.

Tributyl(4-((4-(2-fluoroethoxy)phenoxy)methyl)phenyl)stannane (8b)

A mixture of **7c** (650.3 mg, 2.0 mmol), $(Bu_3Sn)_2$ (2.32 g, 4.0 mmol), $(Ph_3P)_4Pd$ (231.7 mg, 0.2 mmol) and Et_3N (1 mL) in toluene (10 mL) was stirred under reflux overnight. The mixture was concentrated under reduced pressure and purified by silica gel chromatography (petroleum ether/AcOEt = 15/1, v/v) to give a colorless oil of **8b** (325.6 mg, 30.4%). ^1H NMR (400 MHz, $CDCl_3$) δ 7.54 – 7.41 (m, 2H), 7.37 (d, J = 7.8 Hz, 2H), 6.92 (d, J = 9.3 Hz, 2H), 6.87 (d, J = 9.3 Hz, 2H), 4.99 (s, 2H), 4.80 – 4.65 (m, 2H), 4.22 – 4.11 (m, 2H), 1.58 – 1.50 (m, 6H), 1.39 – 1.27 (m, 6H), 1.14 – 0.96 (m, 6H), 0.88 (t, J = 7.3 Hz, 9H); MS (EI): m/z calcd for $C_{27}H_{41}FO_2Sn$ 536; found 536 M^+.

4-(2-Fluoroethoxy)benzaldehyde (9a)

To a solution of 4-hydroxybenzaldehyde (2.44 g, 20 mmol) and 1-bromo-2-fluoroethane (2.54 g, 20 mmol) in anhydrous DMF (5 mL), K_2CO_3 (5.53 g, 40 mmol) was added. The resulting mixture was stirred at 90 ℃ for 2h, and the solvent was evaporated under reduced pressure. 50 mL of water was added, and the mixture was extracted by CH_2Cl_2 (3 × 10 mL). Combined organic layers were dried over anhydrous $MgSO_4$, filtered and concentrated under a vacuum. The crude mixture was purified by silica gel chromatography (petroleum ether/AcOEt = 4/1, v/v) to give a white solid of **9a** (2.95 g, 87.8%). mp: 53.3-54.5 ℃; ^1H NMR (400 MHz, $CDCl_3$) δ 9.90 (s, 1H), 7.86 (d, J = 8.7 Hz, 2H), 7.04 (d, J = 8.6 Hz, 2H), 4.88 – 4.71 (m, 2H), 4.37 – 4.24 (m, 2H); MS (EI): m/z calcd for $C_9H_9FO_2$ 168; found 168 M^+.

4-(2-Bromoethoxy)benzaldehyde (9b)

The procedure described above for the preparation of **9a** was employed to obtain a white solid of **9b** from 4-hydroxybenzaldehyde and 1,2-dibromoethane (1.32 g, 28.9%). mp:

51.2-51.7 ℃. ^{1}H NMR (400 MHz, $CDCl_3$) δ 9.90 (s, 1H), 7.85 (d, *J* = 8.8 Hz, 2H), 7.02 (d, *J* = 8.7 Hz, 2H), 4.38 (t, *J* = 6.2 Hz, 2H), 3.67 (t, *J* = 6.2 Hz, 2H).

(4-(2-Fluoroethoxy)phenyl)methanol (10a)

To a stirring mixture of **9a** (2.95 g, 17.6 mmol) in anhydrous MeOH (10 mL) in ice bath, $NaBH_4$ (1.33 g, 35.2 mmol) was slowly added. The reaction mixture was stirred for 30 min at 0 ℃ and 10 mL of water was added to quench the reaction. MeOH was evaporated under reduced pressure and the mixture was neutralized with 1 M HCl and then extracted by CH_2Cl_2 (3 × 10 mL). Combined organic layers were dried over anhydrous $MgSO_4$, filtered and concentrated under reduced pressure to give a yellow oil of **6a** (2.73 g, 91.1%). mp: 50.6-51.5 ℃; ^{1}H NMR (400 MHz, $CDCl_3$) δ 7.31 (d, *J* = 8.5 Hz, 2H), 6.93 (d, *J* = 8.5 Hz, 2H), 4.84 – 4.68 (m, 2H), 4.63 (s, 2H), 4.27 – 4.17 (m, 2H); MS (EI): m/z calcd for $C_9H_{11}FO_2$ 170; found 170 M^+.

(4-(2-Bromoethoxy)phenyl)methanol (10b)

The procedure described above for the preparation of **10a** was employed to obtain a white solid of **10b** from **9b** (1.14 g, 95.8%). mp: 88.7-89.6 ℃; ^{1}H NMR (400 MHz, $CDCl_3$) δ 7.30 (d, *J* = 8.5 Hz, 2H), 6.91 (d, *J* = 8.6 Hz, 2H), 4.63 (s, 2H), 4.30 (t, *J* = 6.3 Hz, 2H), 3.64 (t, *J* = 6.3 Hz, 2H); MS (EI): m/z calcd for $C_9H_{11}BrO_2$ 230; found 230 M^+.

1-(Bromomethyl)-4-(2-fluoroethoxy)benzene (11a)

To a stirring solution of **10a** (2.73 g, 16.0 mmol) in anhydrous CH_2Cl_2 (25 mL) at 0 ℃, phosphorus tribromide (4.33 g, 16.0 mmol) was added dropwise. The resulting mixture was stirred at room temperature for 30 min and the reaction was quenched by addition of 20 mL of water. The mixture was neutralized with $NaHCO_3$ and then extracted by CH_2Cl_2 (3 × 10

mL). Combined organic layers were dried over anhydrous $MgSO_4$, filtered and concentrated under reduced pressure to give a colorless oil of **11a** (3.54 g, 95.0%). ^{1}H NMR (400 MHz, $CDCl_3$) δ 7.33 (d, *J* = 8.6 Hz, 2H), 6.89 (d, *J* = 8.6 Hz, 2H), 4.84 – 4.68 (m, 2H), 4.50 (s, 2H), 4.27 – 4.16 (m, 2H); MS (EI): m/z calcd for $C_9H_{11}BrFO$ 232; found 232 M^+.

1-(2-Bromoethoxy)-4-(bromomethyl)benzene (11b)

The procedure described above for the preparation of **11a** was employed to obtain a white solid of **11b** from **10b** (1.29 g, 99.0%). mp: 53.9-54.7 ℃. ^{1}H NMR (400 MHz, $CDCl_3$) δ 7.33 (d, *J* = 8.7 Hz, 2H), 6.87 (d, *J* = 8.7 Hz, 2H), 4.49 (s, 2H), 4.29 (t, *J* = 6.3 Hz, 2H), 3.63 (t, *J* = 6.3 Hz, 2H); MS (EI): m/z calcd for $C_9H_{10}Br_2O$ 292; found 292 M^+.

1-(2-Fluoroethoxy)-4-((4-iodophenoxy)methyl)benzene (12a)

The procedure described above for the preparation of **7a** was employed to obtain a white solid of **12a** from 4-iodophenol and **11a** (623.4 mg, 94.1%). mp: 127.6-128.2 ℃; ^{1}H NMR (400 MHz, $CDCl_3$) δ 7.55 (d, *J* = 8.9 Hz, 2H), 7.34 (d, *J* = 8.6 Hz, 2H), 6.94 (d, *J* = 8.6 Hz, 2H), 6.74 (d, *J* = 8.9 Hz, 2H), 4.95 (s, 2H), 4.83 – 4.68 (m, 2H), 4.27 – 4.16 (m, 2H); ^{13}C NMR (101 MHz, $CDCl_3$) δ 158.65 (C_q), 158.39 (C_q), 138.23 (2 × CH), 129.24 (C_q), 129.21 (2 × CH), 117.34 (2 × CH), 114.81 (2 × CH), 83.00 (C_q), 81.87 (d, *J* = 170.8 Hz, CH_2), 69.80 (CH_2), 67.20 (d, *J* = 20.6 Hz, CH_2); HRMS (EI): m/z calcd for $C_{15}H_{14}FIO_2$ 372.0023; found 372.0028 M^+.

1-(2-Bromoethoxy)-4-((4-iodophenoxy)methyl)benzene (12b)

The procedure described above for the preparation of **7a** was employed to obtain a white solid of **12b** from 4-iodophenol and **11b** (493.7 mg, 92.7%). mp: 118.6-119.7 ℃; ^{1}H NMR (400 MHz, $CDCl_3$) δ 7.55 (d, *J* = 8.9 Hz, 2H), 7.34 (d, *J* = 8.6 Hz, 2H), 6.92 (d, *J* = 8.6 Hz,

2H), 6.74 (d, *J* = 8.9 Hz, 2H), 4.96 (s, 2H), 4.30 (t, *J* = 6.3 Hz, 2H), 3.64 (t, *J* = 6.3 Hz, 2H); MS (EI): m/z calcd for $C_{15}H_{14}BrIO_2$ 432; found 432 M^+.

1-Bromo-4-((4-(2-fluoroethoxy)benzyl)oxy)benzene (12c)

The procedure described above for the preparation of **7a** was employed to obtain a white solid of **12c** from 4-bromophenol and **11a** (1.03 g, 76.1%). mp: 116.7-117.3 ℃; ^{1}H NMR (400 MHz, $CDCl_3$) δ 7.39 – 7.31 (m, 4H), 6.94 (d, *J* = 8.0 Hz, 2H), 6.84 (d, *J* = 8.2 Hz, 2H), 4.96 (s, 2H), 4.84 – 4.68 (m, 2H), 4.28 – 4.17 (m, 2H); MS (EI): m/z calcd for $C_{15}H_{14}BrFO_2$ 324; found 324 M^+.

2-(4-((4-Iodophenoxy)methyl)phenoxy)ethyl 4-methylbenzenesulfonate (13a)

A mixture of **12b** (402.5 mg, 0.93 mmol) and silver *p*-toluenesulfonate (519.1 mg, 1.86 mmol) in acetonitrile (20 mL) was stirred for 12 h at 90 ℃. The mixture was concentrated under reduced pressure and purified by silica gel chromatography (petroleum ether/AcOEt = 4/1) to give a white solid of **13a** (277.6 mg, 57.0%). m.p. 141.7-142.5 ℃; ^{1}H NMR (400 MHz, $CDCl_3$) δ 7.82 (d, *J* = 8.3 Hz, 2H), 7.55 (d, *J* = 8.9 Hz, 2H), 7.34 (d, *J* = 8.0 Hz, 2H), 7.29 (d, *J* = 8.6 Hz, 2H), 6.79 (d, *J* = 8.6 Hz, 2H), 6.73 (d, *J* = 8.9 Hz, 2H), 4.94 (s, 2H), 4.39 – 4.35 (m, 2H), 4.17 – 4.14 (m, 2H), 2.45 (s, 3H); MS (EI): m/z calcd for $C_{22}H_{21}IO_5S$ 524; found 524 M^+.

Tributyl(4-((4-(2-fluoroethoxy)benzyl)oxy)phenyl)stannane (13b)

The procedure described above for the preparation of **8b** was used to obtain a colorless oil of **13b** from **12c** (134.6 mg, 25.1%). ^{1}H NMR (400 MHz, $CDCl_3$) δ 7.37 (d, *J* = 8.7 Hz, 2H), 7.32 – 7.26 (m, 2H), 6.99 – 6.93 (m, 4H), 5.00 (s, 2H), 4.83 – 4.68 (m, 2H), 4.28 – 4.17 (m,

2H), 1.58 – 1.50 (m, 6H), 1.39 – 1.27 (m, 6H), 1.14 – 0.96 (m, 6H), 0.88 (t, J = 7.3 Hz, 9H);

MS (EI): m/z calcd for $C_{27}H_{41}FO_2Sn$ 536; found 536 M^+.

Supplementary Tables

Table S1 | Crystal data and structure refinements for compound **12a** (CCDC 1063679)

	**12a**
**Data collection**	
Formula sum	$C_{15}H_{14}FIO_2$
Formula weight (g/mol)	372.16
Crystal system	monoclinic
Space group	P2(1)/c
Cell dimensions	
a, *b*, *c* (Å)	17.65, 10.49, 7.66
α, β, γ (°)	90, 96.4, 90
Cell volume ($Å^3$)	1408.94
Z	4
F(000)	728
Crystal size (mm)	0.27×0.20×0.11
Calc. density (g/cm^3)	1.754
Completeness (%)	99.9
**Refinement**	
$R[F^2 > 2\sigma(F^2)]$	0.0180
$wR(F^2)$	0.0409
GoF	1.036
Reflections	2550
Parameters	172
Restraints	0

Table S2 | Purity of key target compounds

**Compounds**	**Flow rate (mL/min)**	**Mobile phase (CH_3CN %)**	**Column (Venusil MP C18)**	**Retention time (RT, min)**	**Purity (%)**
**7a**	1	80	4.6 ×250 mm	8.81	99.4
[^{125}I]**7a**	1	80	4.6 ×250 mm	9.28	99.2
**7a**	1	80	4.6 ×250 mm	8.12	99.2
[^{18}F]**7a**	1	80	4.6 ×250 mm	8.53	99.7
**12a**	1	80	4.6 ×250 mm	8.21	99.7
[^{125}I]**12a**	1	80	4.6 ×250 mm	8.63	99.0
**12a**	1	80	4.6 ×250 mm	8.96	99.2
[^{18}F]**12a**	1	80	4.6 ×250 mm	9.61	99.9

Table S3 | Biodistribution of radioactivity after intravenous injection of [125I]**7a**, [18F]**7a**, [125I]**12a** and [18F] **12a** in normal ICR mice [a]

Organ	2 min	10 min	30 min	60 min
[125I]**7a** (log *D* = 3.96 ±0.22, SA ≈ 81 GBq/*μ*mol)				
blood	5.03 ±0.20	3.03 ±0.48	2.17 ±0.31	1.96 ±0.31
brain	7.04 ±0.89	2.73 ±0.25	1.05 ±0.22	0.55 ±0.11
heart	9.70 ±2.25	2.01 ±0.14	1.65 ±0.43	1.00 ±0.18
liver	22.45 ±3.88	14.43 ±2.37	12.35 ±2.83	5.15 ±1.09
spleen	3.86 ±0.21	1.32 ±0.13	1.26 ±0.20	1.14 ±0.56
lung	10.78 ±2.33	7.44 ±1.59	4.25 ±0.41	4.04 ±1.63
kidney	12.03 ±1.20	9.53 ±1.58	6.71 ±0.78	6.56 ±1.10
pancreas	8.15 ±1.09	2.07 ±0.30	2.61 ±0.59	1.97 ±0.48
muscle	3.72 ±0.51	1.28 ±0.11	1.29 ±0.31	1.36 ±0.25
thyroid [b]	0.12 ±0.02	0.09 ±0.02	0.12 ±0.01	0.16 ±0.03
stomach [b]	1.32 ±0.21	1.08 ±0.24	4.38 ±1.00	2.44 ±0.97
intestine [b]	6.46 ±0.79	17.21 ±2.33	19.88 ±4.22	20.21 ±7.78
[125I]**7a** (log *D* = 3.96 ±0.22, SA ≈ 60 GBq/*μ*mol)				
blood	3.30 ±0.43	2.31 ±0.22	1.39 ±0.15	0.83 ±0.06
brain	5.39 ±0.36	3.51 ±0.36	0.97 ±0.11	0.37 ±0.05
heart	7.34 ±0.69	2.32 ±0.15	0.96 ±0.13	0.55 ±0.10
liver	12.12 ±2.01	11.30 ±0.58	6.29 ±0.22	3.31 ±0.37
spleen	3.63 ±0.83	1.53 ±0.23	0.62 ±0.03	0.36 ±0.03
lung	7.77 ±0.18	6.50 ±0.69	4.30 ±0.58	1.91 ±0.29
kidney	9.80 ±1.05	10.01 ±1.43	6.25 ±1.23	3.40 ±0.48
pancreas	6.79 ±1.53	2.79 ±0.35	1.06 ±0.16	0.57 ±0.05
muscle	3.37 ±0.50	1.42 ±0.20	0.95 ±0.08	0.72 ±0.20
thyroid [b]	0.12 ±0.03	0.11 ±0.01	0.12 ±0.04	0.22 ±0.05
stomach [b]	1.15 ±0.21	0.74 ±0.09	1.67 ±0.33	1.04 ±0.30
intestine [b]	4.57 ±0.96	8.51 ±0.48	18.87 ±2.27	25.30 ±3.94
[18F]**7a** (log *D* = 3.88 ±0.17, SA ≈ 60 GBq/*μ*mol)				
blood	5.99 ±0.16	4.29 ±0.14	4.36 ±0.20	4.97 ±0.12
brain	6.14 ±0.52	4.78 ±0.11	3.85 ±0.24	3.48 ±0.12
heart	9.32 ±0.27	4.03 ±0.34	4.20 ±0.52	4.26 ±0.13
liver	17.83 ±1.41	6.07 ±0.27	4.31 ±0.32	3.84 ±0.21
spleen	5.92 ±1.30	3.33 ±0.20	3.38 ±0.23	3.28 ±0.29
lung	9.34 ±0.45	4.63 ±0.35	4.21 ±0.18	4.32 ±0.17
kidney	12.70 ±0.69	7.69 ±1.15	6.12 ±0.79	4.77 ±0.52
pancreas	8.18 ±0.79	3.96 ±0.15	3.62 ±0.30	2.89 ±0.37
muscle	5.03 ±0.63	3.46 ±0.19	3.82 ±0.40	4.31 ±0.38
bone	2.53 ±0.43	1.55 ±0.50	2.70 ±0.48	4.16 ±0.59
stomach [b]	1.55 ±0.11	1.24 ±0.13	1.82 ±0.48	1.58 ±0.18
intestine [b]	6.38 ±1.04	5.38 ±0.33	5.95 ±0.89	8.13 ±1.01
[125I]**12a** (log *D* = 3.62 ±0.15, SA ≈ 81 GBq/*μ*mol)				
blood	4.96 ±0.35	3.88 ±0.79	3.89 ±0.53	3.01 ±1.07

brain	5.27 ± 0.98	2.28 ± 0.27	0.81 ± 0.06	0.37 ± 0.06
heart	6.61 ± 1.42	2.12 ± 0.16	1.67 ± 0.09	1.17 ± 0.45
liver	18.12 ± 2.34	12.07 ± 1.06	8.68 ± 1.02	4.64 ± 0.77
spleen	2.85 ± 0.30	1.29 ± 0.06	1.03 ± 0.10	0.84 ± 0.31
lung	10.30 ± 2.16	4.16 ± 0.26	3.56 ± 0.38	3.11 ± 1.02
kidney	9.16 ± 0.59	7.61 ± 1.23	7.44 ± 0.90	6.09 ± 2.17
pancreas	6.45 ± 0.56	2.44 ± 0.28	1.75 ± 0.10	1.37 ± 0.41
muscle	3.47 ± 0.45	1.31 ± 0.12	0.99 ± 0.17	1.20 ± 0.26
thyroid [b]	0.13 ± 0.02	0.13 ± 0.01	0.19 ± 0.04	0.34 ± 0.10
stomach [b]	1.30 ± 0.14	1.34 ± 0.16	2.02 ± 0.49	2.27 ± 0.40
intestine [b]	4.81 ± 0.97	10.68 ± 2.60	16.61 ± 3.88	18.84 ± 8.75
$[^{18}F]$**12a** (log *D* = 3.84 ± 0.07, SA ≈ 60 GBq/μmol)				
blood	7.53 ± 0.80	4.55 ± 0.36	5.91 ± 0.33	5.87 ± 0.19
brain	6.76 ± 0.41	5.73 ± 0.44	5.02 ± 0.32	4.26 ± 0.18
heart	9.11 ± 1.07	4.40 ± 0.36	5.41 ± 0.36	4.76 ± 0.40
liver	13.20 ± 1.45	4.74 ± 0.36	4.84 ± 0.28	4.64 ± 0.21
spleen	4.98 ± 0.32	3.55 ± 0.26	4.29 ± 0.36	3.87 ± 0.34
lung	9.17 ± 1.10	4.91 ± 0.60	5.34 ± 0.31	5.19 ± 0.36
kidney	11.63 ± 0.86	6.19 ± 0.46	6.19 ± 0.37	5.48 ± 0.41
pancreas	8.75 ± 0.33	3.96 ± 0.27	4.55 ± 0.36	3.95 ± 0.25
muscle	5.38 ± 0.47	3.92 ± 0.41	4.71 ± 0.50	4.51 ± 0.49
bone	2.55 ± 0.29	2.13 ± 0.27	3.31 ± 0.21	3.51 ± 0.78
stomach [b]	1.70 ± 0.08	1.54 ± 0.32	2.09 ± 0.32	1.70 ± 0.29
intestine [b]	7.72 ± 0.54	6.94 ± 0.53	8.09 ± 0.43	8.50 ± 1.23

[a] Expressed as % injected dose per gram. Each value represents the mean ± SD for 4-5 mice at each interval.

[b] Expressed as % injected dose per organ.

Table S4 | Percentages of metabolites extracted from the brain, plasma, liver, urine and feces of ICR mice after intravenous injection of [^{125}I]**7a** and [^{18}F]**7a**

**Organ**	**Post-injection time (min)**	**[^{125}I]7a**			**[^{18}F]7a**	
		**Metabolite [^{125}I]7a-1**	**Metabolite [^{125}I]7a-2**	**Parent tracer [^{125}I]7a**	**Metabolite [^{18}F]7a-1 and 2**	**Parent tracer [^{18}F]7a**
**Brain**	2	4.1%	0.0%	95.9%	15.3%	84.7%
	10	5.1%	8.9%	86.0%	30.2%	69.8%
	30	10.0%	10.6%	79.4%	31.1%	68.9%
	60	10.3%	17.6%	72.1%	66.0%	34.0%
**Plasma**	2	14.8%	0.0%	85.2%	56.9%	43.1%
	10	58.7%	0.0%	41.3%	78.4%	21.6%
	30	77.9%	0.0%	22.1%	78.2%	21.8%
	60	87.7%	0.0%	12.3%	92.8%	7.2%
**Liver**	2	60.5%	4.3%	35.2%	20.5%	79.5%
	10	72.3%	14.2%	13.5%	37.4%	62.6%
	30	86.3%	4.9%	8.8%	51.3%	48.7%
	60	93.0%	3.7%	3.3%	76.6%	23.4%
**Urine**	2	0.0%	0.0%	0.0%	100%	0.0%
	10	97.4%	0.0%	2.6%	100%	0.0%
	30	100%	0.0%	0.0%	100%	0.0%
	60	100%	0.0%	0.0%	100%	0.0%
**Feces**	2	77.1%	0.0%	22.9%	46.4%	53.6%
	10	41.2%	0.0%	58.8%	81.2%	18.8%
	30	98.0%	0.0%	2.0%	89.5%	10.5%
	60	91.7%	8.3%	0.0%	92.7%	7.3%

Table S5 | Percentages of metabolites extracted from the brain, plasma, liver, urine and feces of ICR mice after intravenous injection of [^{125}I]**12a** and [^{18}F]**12a**

**Organ**	**Post-injection time (min)**	**[^{125}I]12a**			**[^{18}F]12a**	
		**Metabolite [^{125}I]12a-1**	**Metabolite [^{125}I]12a-2**	**Parent tracer [^{125}I]12a**	**Metabolite [^{18}F]12a-1 and 2**	**Parent tracer [^{18}F]12a**
**Brain**	2	7.9%	0.0%	92.1%	12.1%	87.9%
	10	8.2%	9.7%	82.1%	34.3%	65.7%
	30	9.6%	12.1%	78.3%	50.8%	49.2%
	60	12.7%	18.7%	68.6%	70.2%	29.8%
**Plasma**	2	11.6%	0.0%	88.4%	26.3%	73.7%
	10	70.5%	0.0%	29.6%	59.6%	40.4%
	30	73.7%	0.0%	26.3%	65.9%	34.1%
	60	95.3%	0.0%	4.68%	86.1%	13.9%
**Liver**	2	48.7%	2.2%	49.1%	8.9%	91.1%
	10	76.7%	1.8%	21.5%	14.7%	85.3%
	30	90.5%	3.2%	6.3%	17.8%	82.2%
	60	94.7%	1.5%	3.8%	78.3%	21.7%
**Urine**	2	0.0%	0.0%	0.0%	100%	0.0%
	10	100%	0.0%	0.0%	100%	0.0%
	30	100%	0.0%	0.0%	100%	0.0%
	60	98.9%	1.1%	0.0%	100%	0.0%
**Feces**	2	0.0%	0.0%	0.0%	53.3%	46.7%
	10	0.0%	0.0%	0.0%	79.1%	20.9%
	30	0.0%	0.0%	0.0%	83.2%	16.8%
	60	0.0%	0.0%	0.0%	82.6%	17.4%

Supplementary Figures

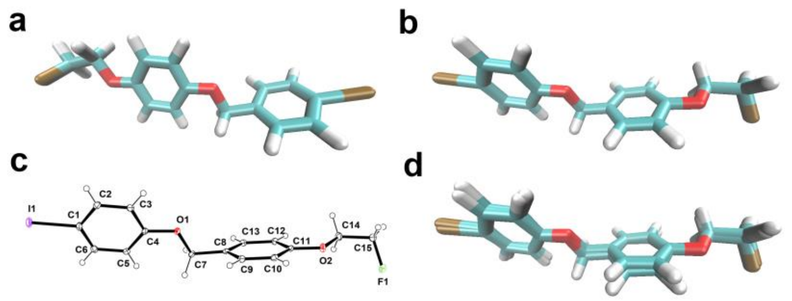

Figure S1 | Chemical structures of 7a and 12a. Optimized structures of benzyloxybenzene derivatives **7a** (a) and **12a** (b). (c) X-ray crystal structure of **12a** (CCDC 1063679). Superposition of **12a** optimized and its X-ray crystal structure was show in d. RMSD = 0.190 Å.

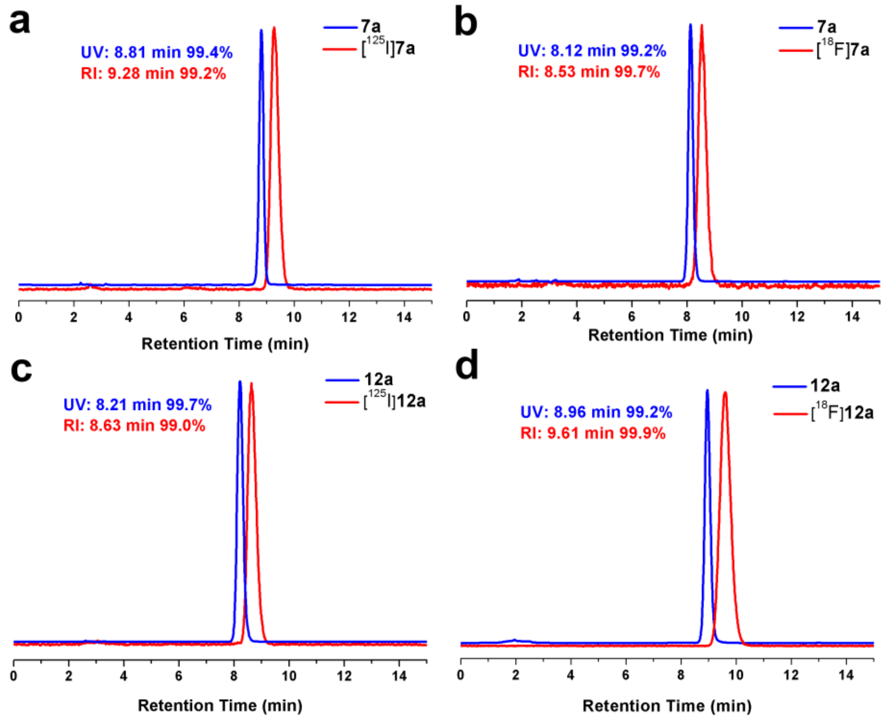

Figure S2 | Co-injection HPLC profiles of 7a and [^{125}I]7a; 7a and [^{18}F]7a; 12a and [^{125}I]12a; 12a and [^{18}F]12a. HPLC conditions: Venusil MP C18 column (Agela Technologies, 5 μm, 4.6 × 250 mm), CH_3CN/H_2O = 80%/20%, 1 mL/min, UV, 254 nm.

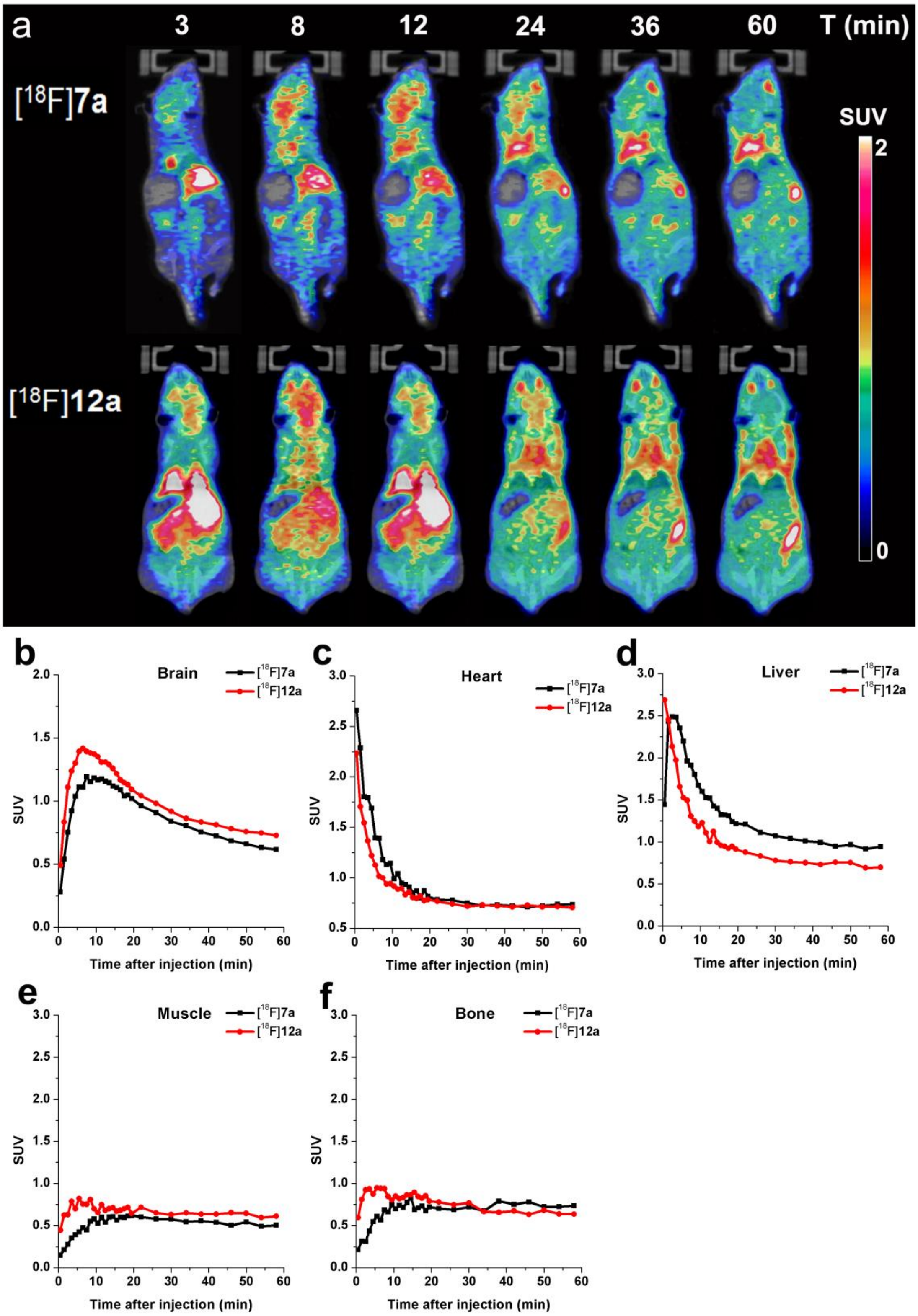

Figure S3 | Dynamic microPET/CT imaging of [^{18}F]7a and [^{18}F]12a in normal ICR mice. (a) Whole body time-radioactivity biodistribution by dynamic microPET/CT imaging. PET image color intensities are expressed as standardized uptake value (SUV). (b-f) Time-activity curves (TACs) of [^{18}F]**7a** and [^{18}F]**12a** in brain, heart, liver, muscle and bone for the entire 60 min PET scan.

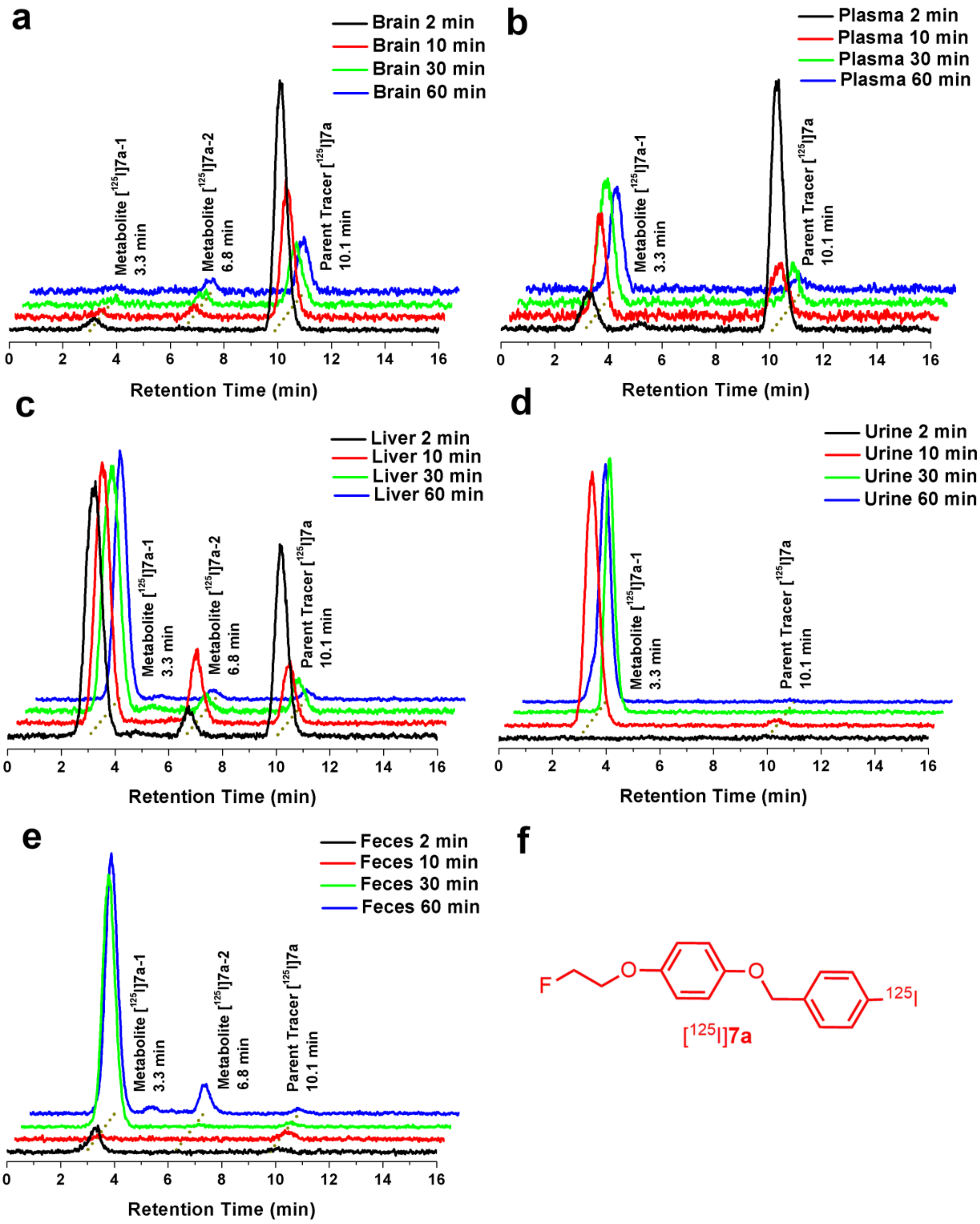

Figure S4 | HPLC profiles for radioactive metabolites of [^{125}I]7a in ICR mice brain (a), plasma (b), liver (c), urine (d) and feces (e) at 2, 10, 30 and 60 min post-injection time points. Reversed-phase HPLC performed on a Venusil MP C18 reverse phase column (Agela Technologies, 5 μm, 4.6 mm × 250 mm) using a binary gradient system (acetonitrile/water : 80%/20%) at a 1.0 mL/min flow rate. (f) Chemical structure of [^{125}I]**7a**.

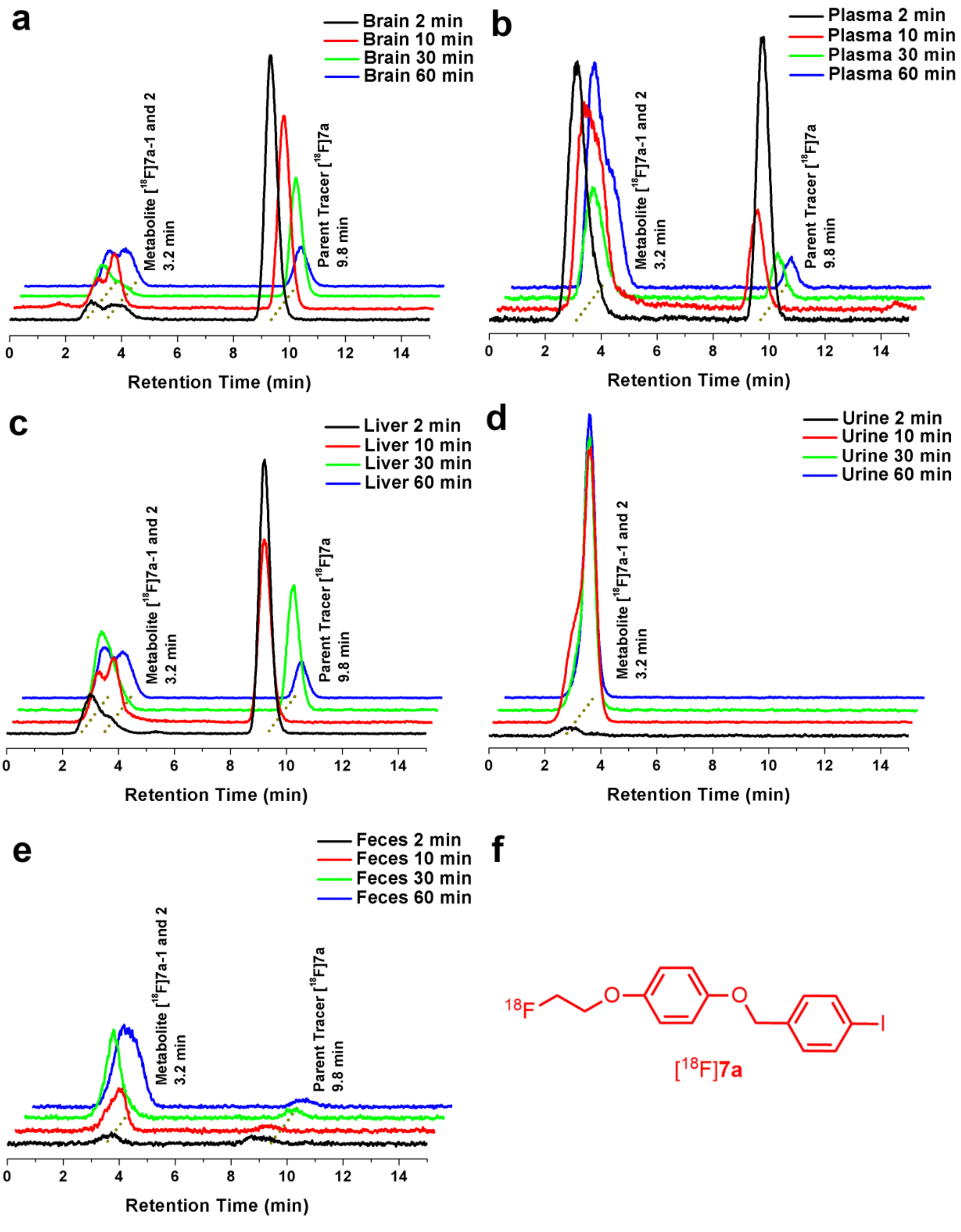

Figure S5 | HPLC profiles for radioactive metabolites of [^{18}F]7a in ICR mice brain (a), plasma (b), liver (c), urine (d) and feces (e) at 2, 10, 30 and 60 min post-injection time points. Reversed-phase HPLC performed on a Venusil MP C18 reverse phase column (Agela Technologies, 5 μm, 4.6 mm × 250 mm) using a binary gradient system (acetonitrile/water : 80%/20%) at a 1.0 mL/min flow rate. (f) Chemical structure of [^{18}F]**7a**.

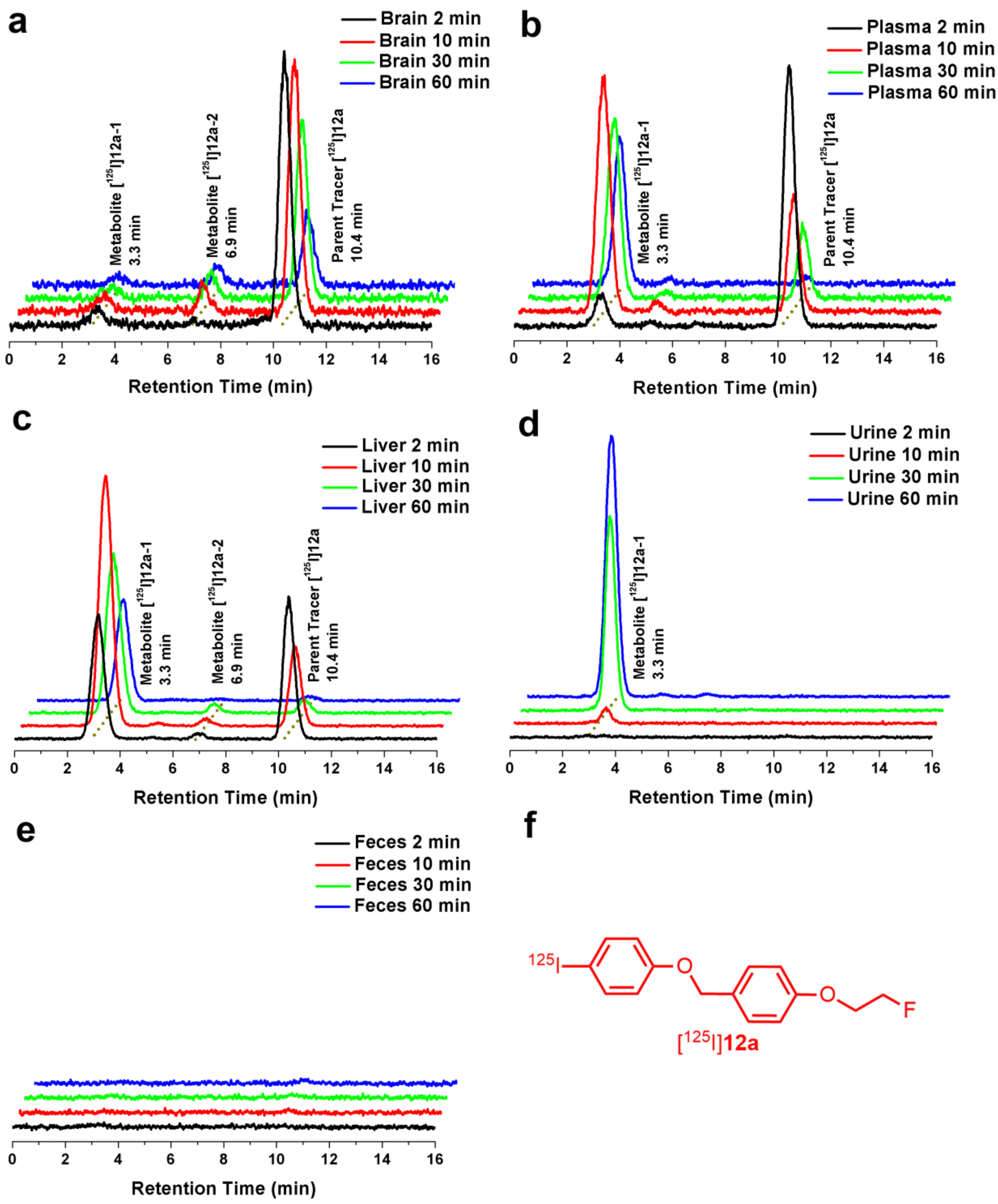

Figure S6 | HPLC profiles for radioactive metabolites of [^{125}I]12a in ICR mice brain (a), plasma (b), liver (c), urine (d) and feces (e) at 2, 10, 30 and 60 min post-injection time points. Reversed-phase HPLC performed on a Venusil MP C18 reverse phase column (Agela Technologies, 5 μm, 4.6 mm × 250 mm) using a binary gradient system (acetonitrile/water : 80%/20%) at a 1.0 mL/min flow rate. (f) Chemical structure of [^{125}I]**12a**.

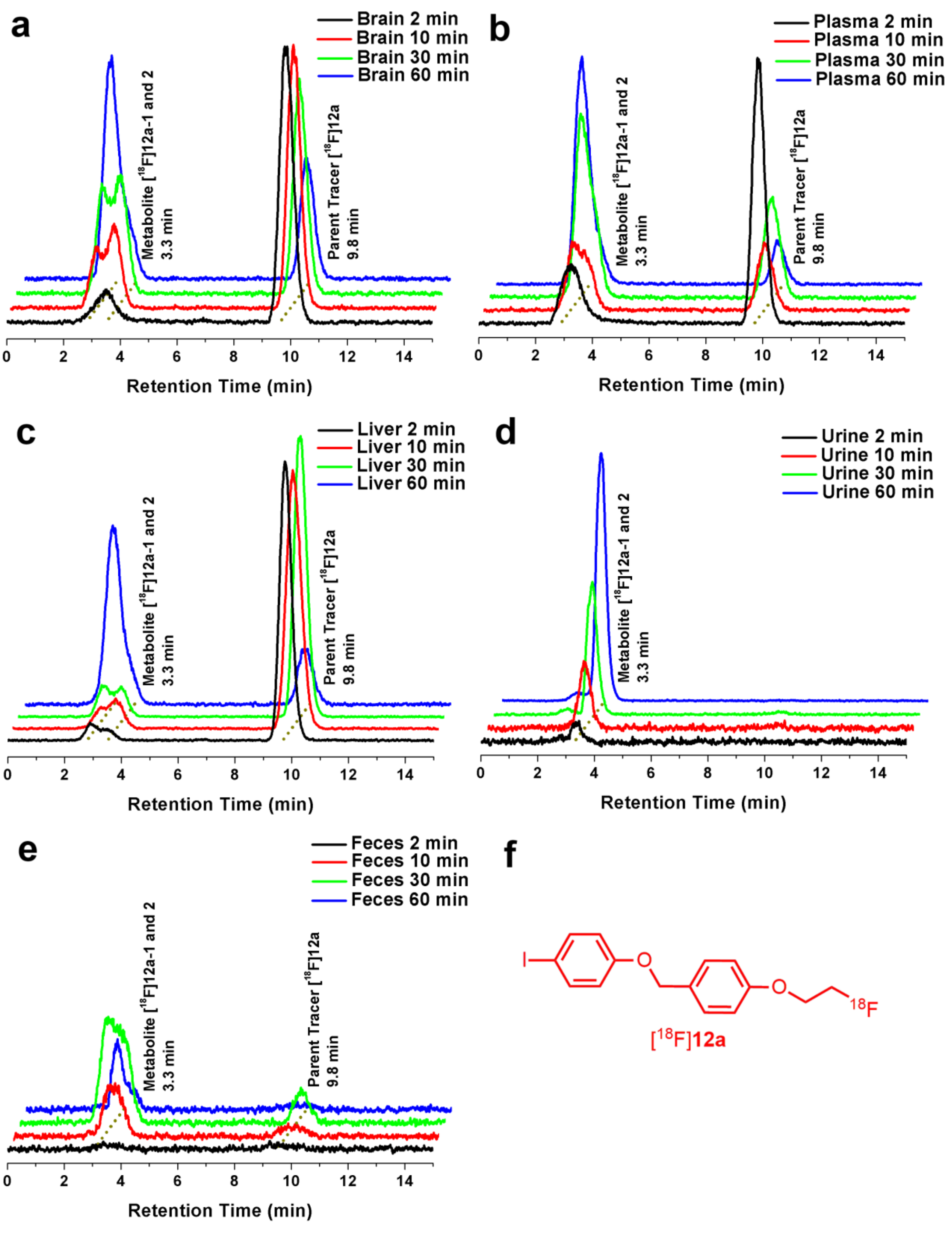

Figure S7 | HPLC profiles for radioactive metabolites of [^{18}F]12a in ICR mice brain (a), plasma (b), liver (c), urine (d) and feces (e) at 2, 10, 30 and 60 min post-injection time points. Reversed-phase HPLC performed on a Venusil MP C18 reverse phase column (Agela Technologies, 5 μm, 4.6 mm × 250 mm) using a binary gradient system (acetonitrile/water : 80%/20%) at a 1.0 mL/min flow rate. (f) Chemical structure of [^{18}F]**12a**.

NMR and MS (EI) Spectra

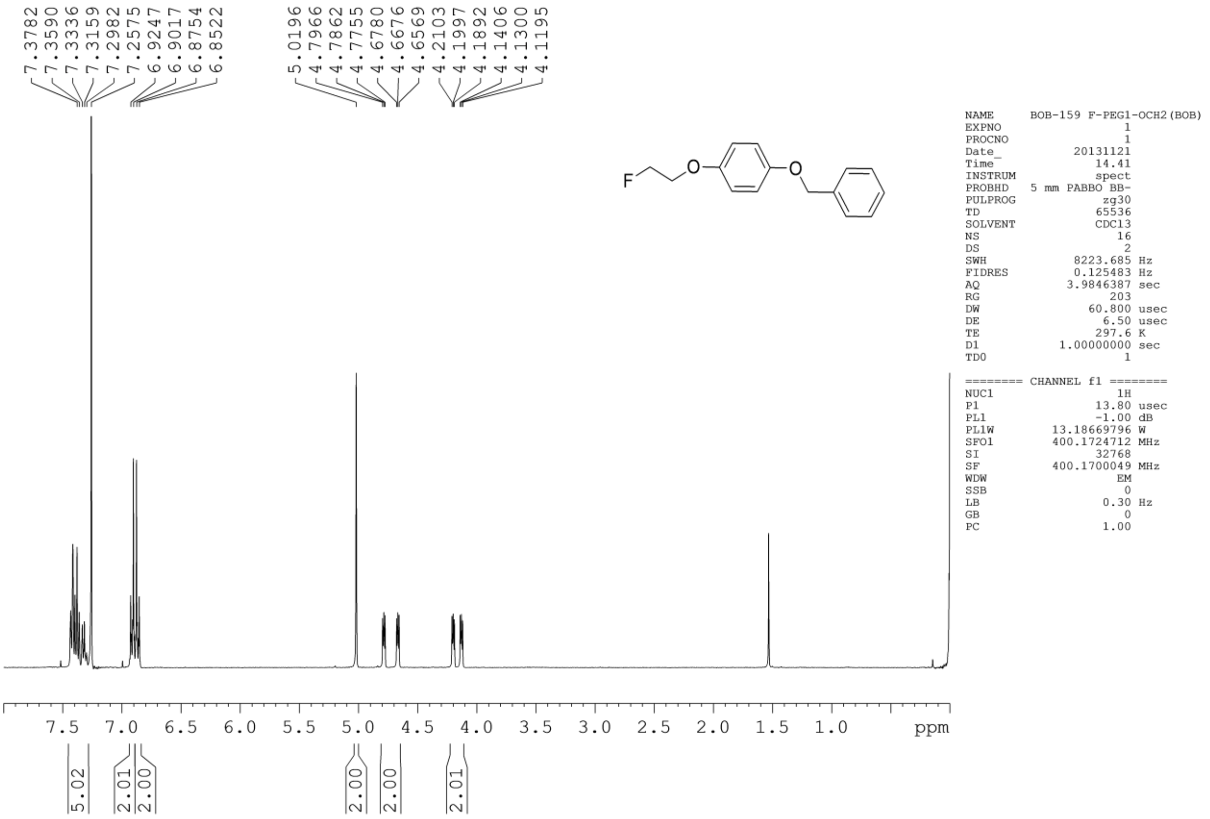

^{1}H NMR (CDCl3) spectrum of 1-(benzyloxy)-4-(2-fluoroethoxy)benzene (**5a**)

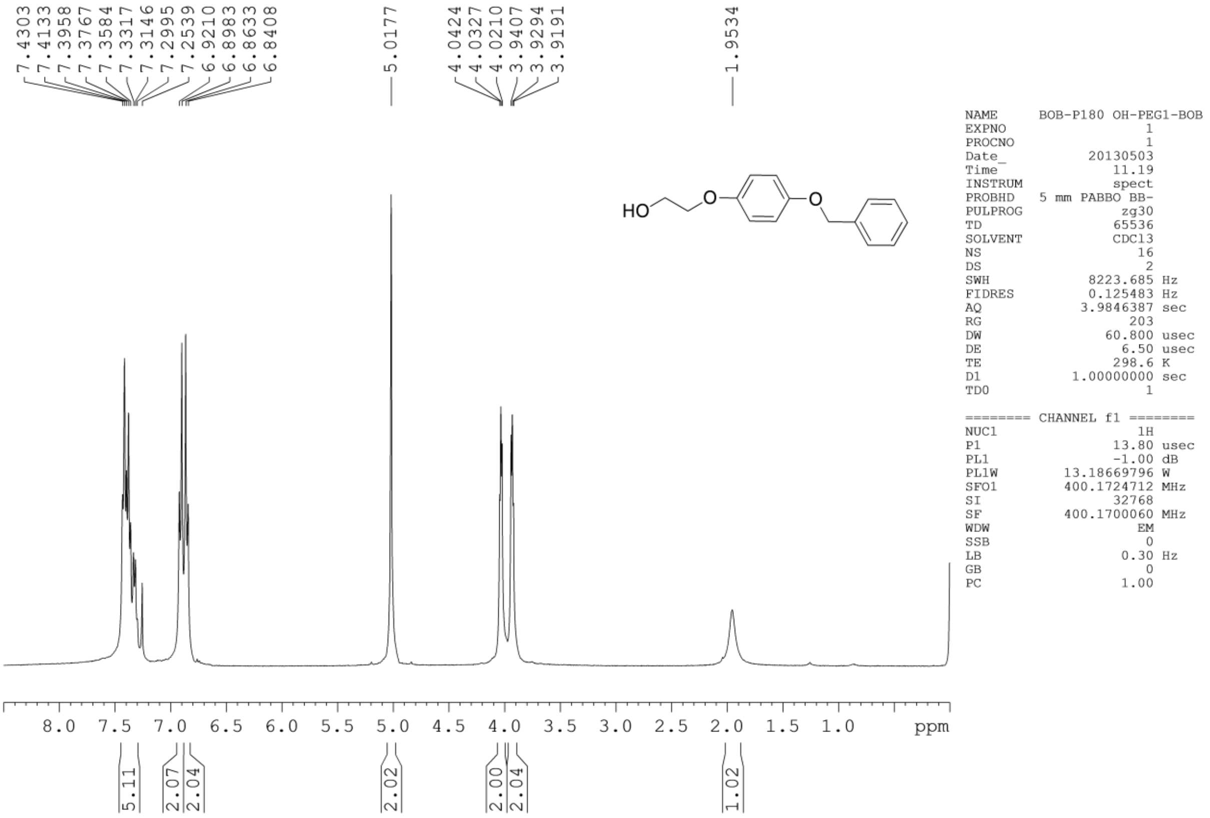

^{1}H NMR ($CDCl_3$) spectrum of 2-(4-(benzyloxy)phenoxy)ethanol (**5b**)

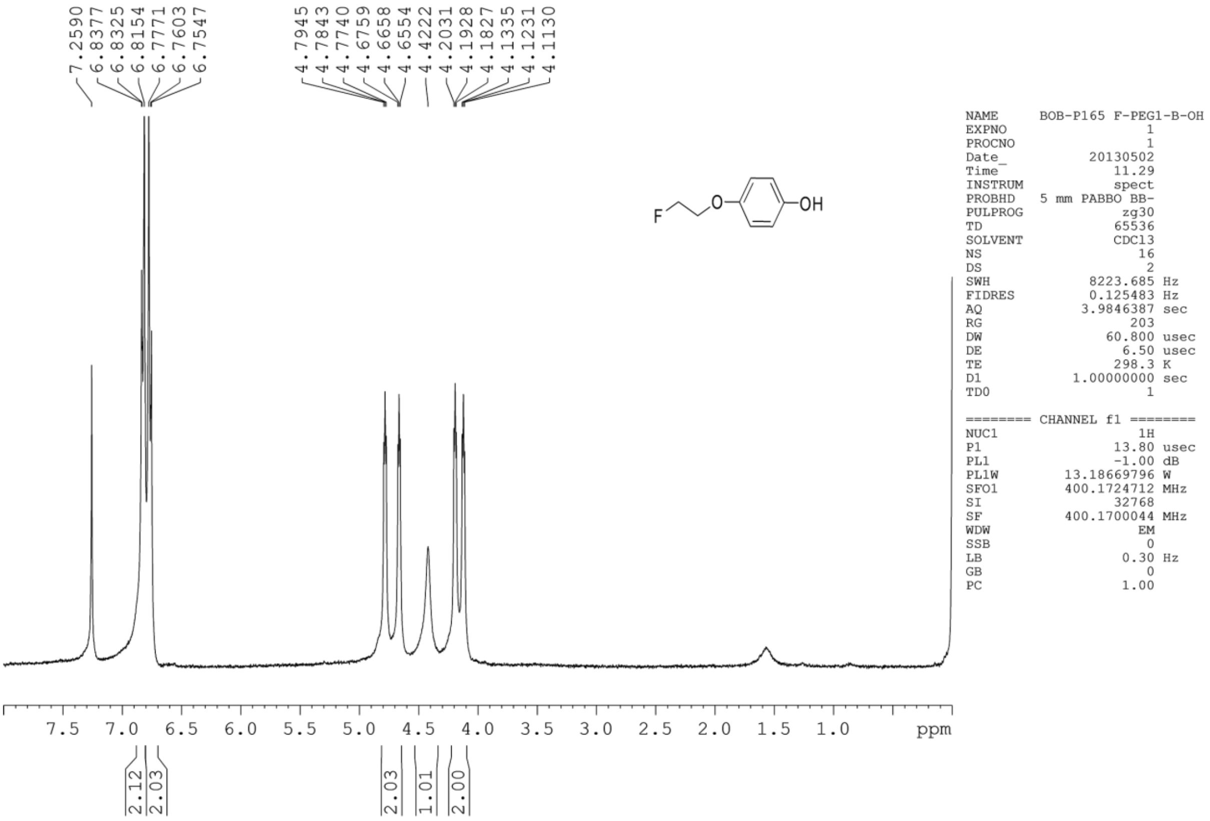

^{1}H NMR ($CDCl_3$) spectrum of 4-(2-fluoroethoxy)phenol (**6a**)

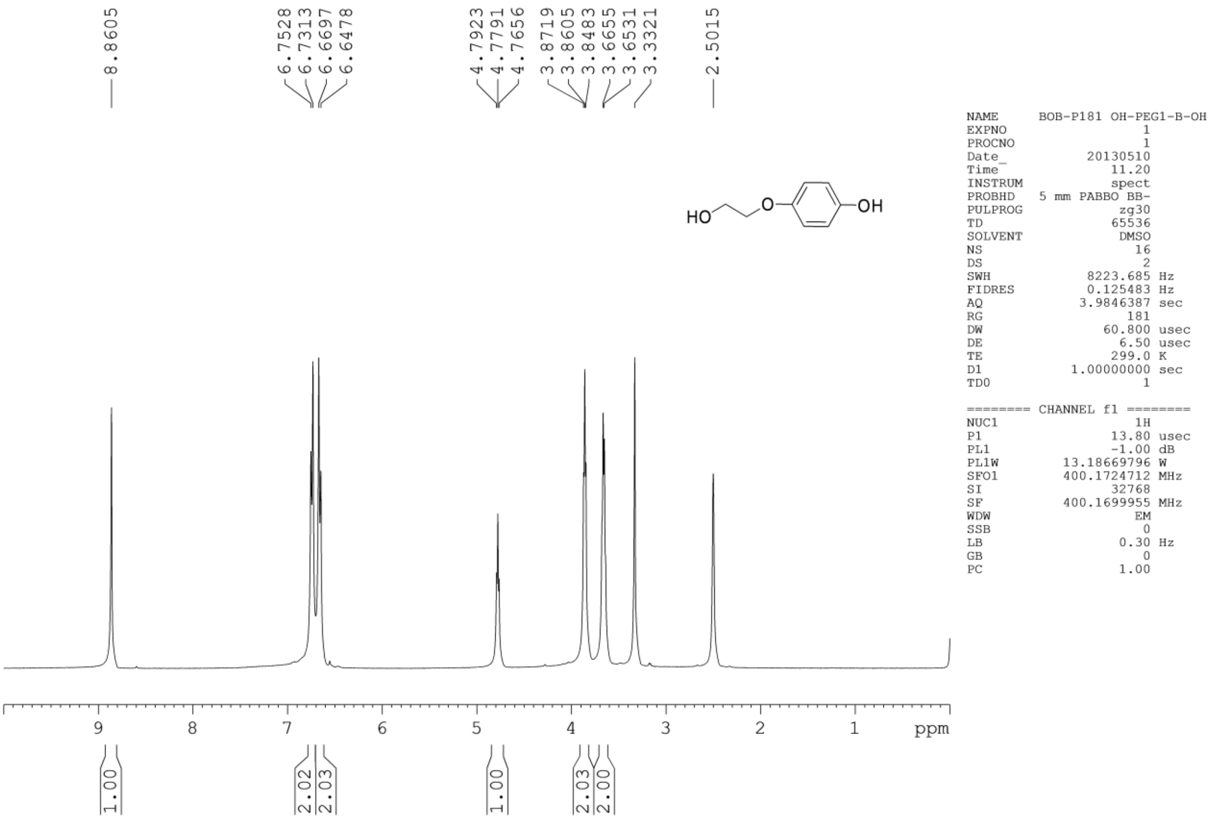

^{1}H NMR (DMSO-d_6) spectrum of 4-(2-hydroxyethoxy)phenol (**6b**)

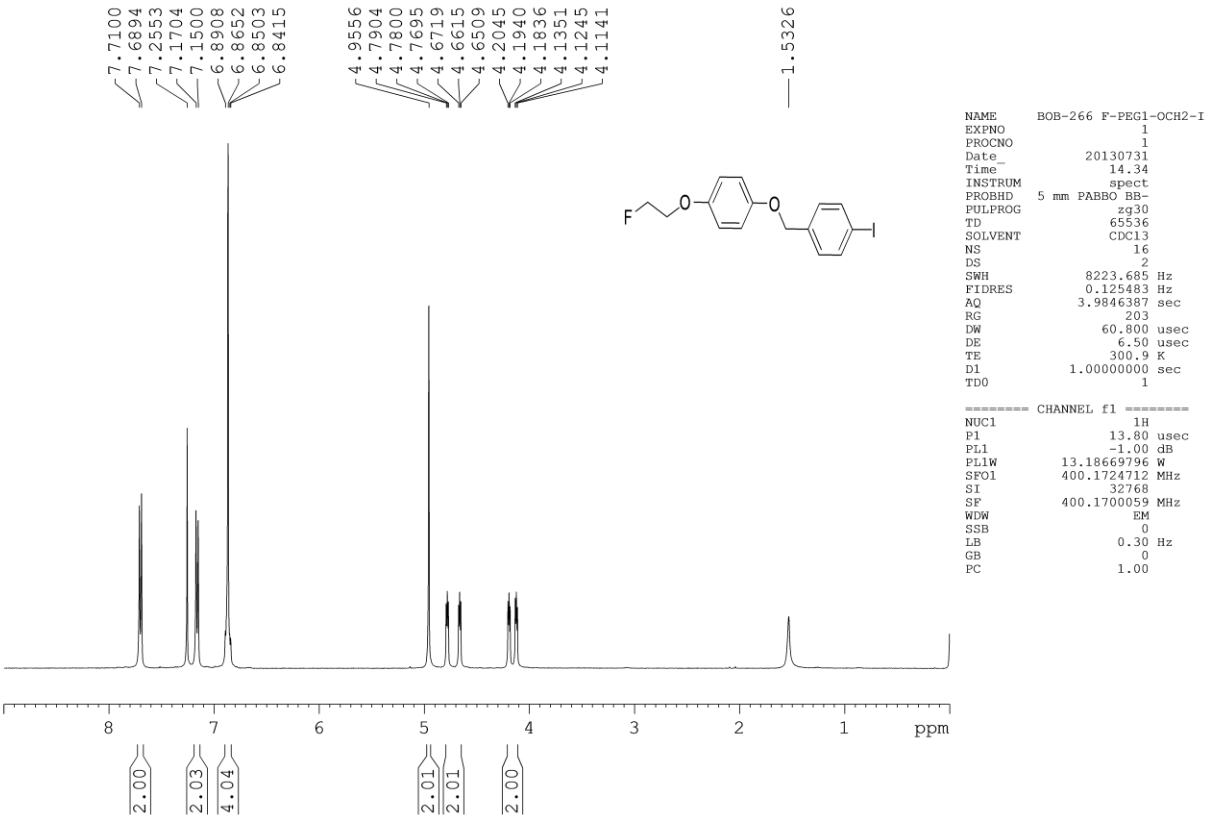

^{1}H NMR ($CDCl_3$) spectrum of 1-(2-fluoroethoxy)-4-((4-iodobenzyl)oxy)benzene (**7a**)

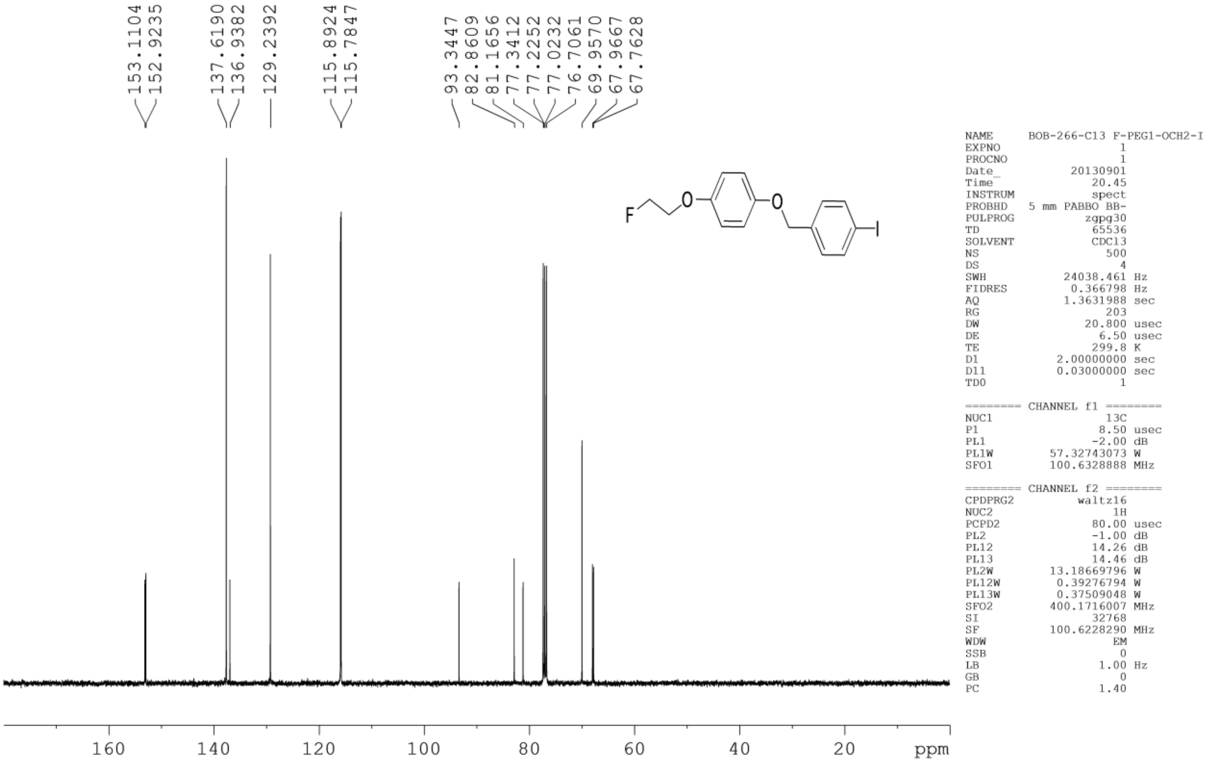

^{13}C NMR ($CDCl_3$) spectrum of 1-(2-fluoroethoxy)-4-((4-iodobenzyl)oxy)benzene (**7a**)

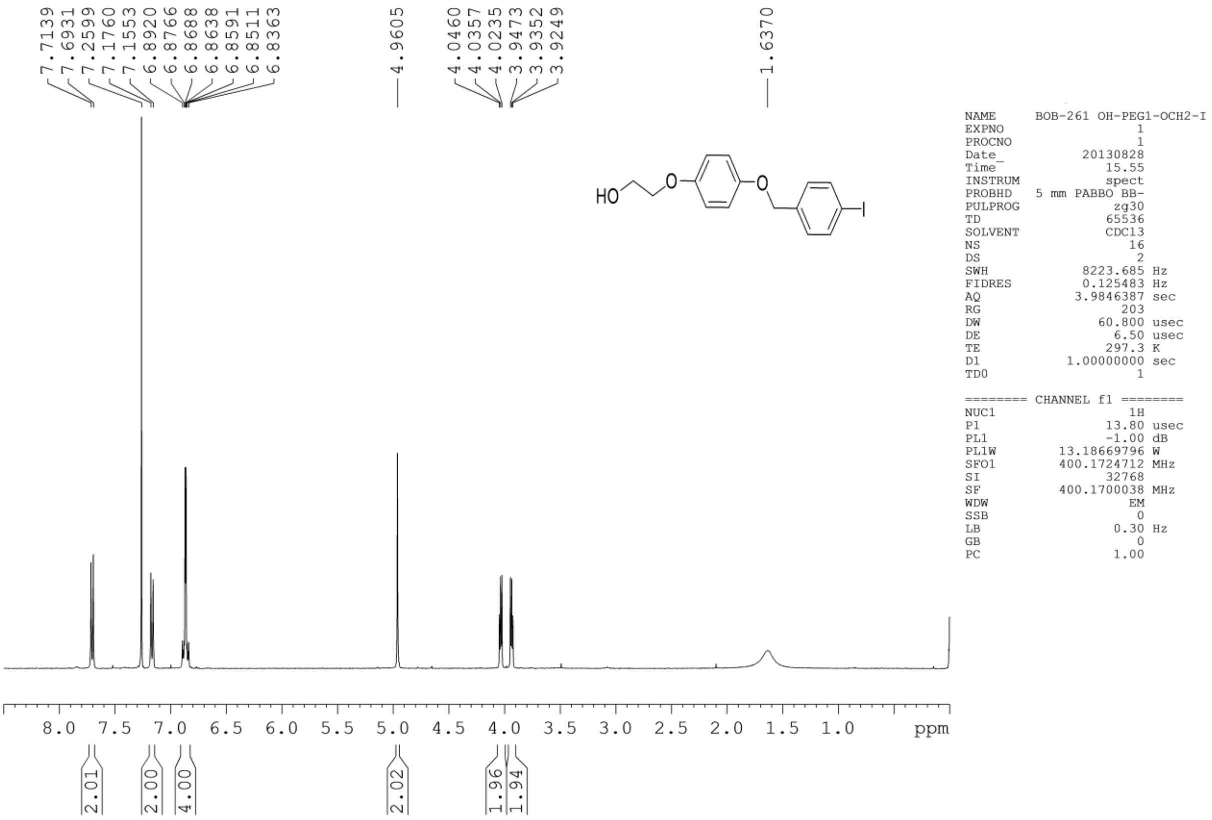

^{1}H NMR ($CDCl_3$) spectrum of 2-(4-((4-iodobenzyl)oxy)phenoxy)ethanol (**7b**)

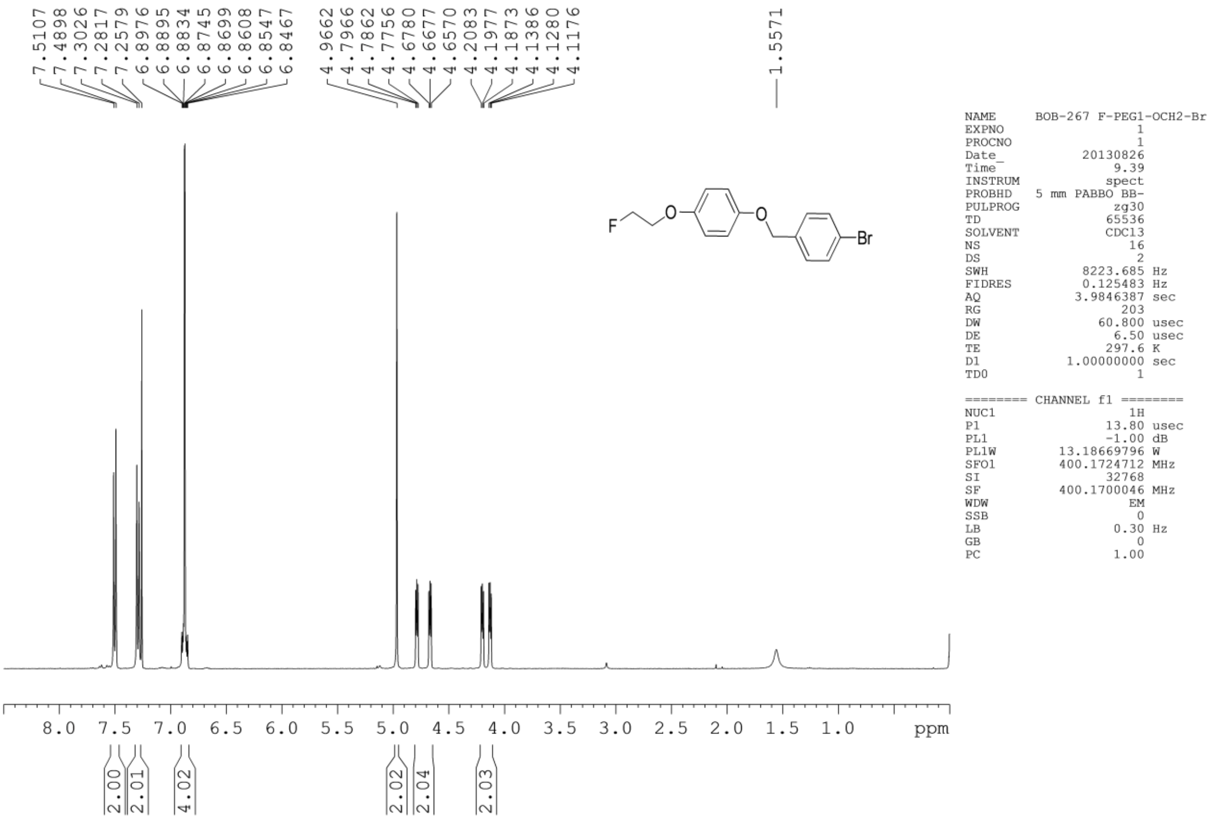

^{1}H NMR ($CDCl_3$) spectrum of 1-bromo-4-((4-(2-fluoroethoxy)phenoxy)methyl)benzene (**7c**)

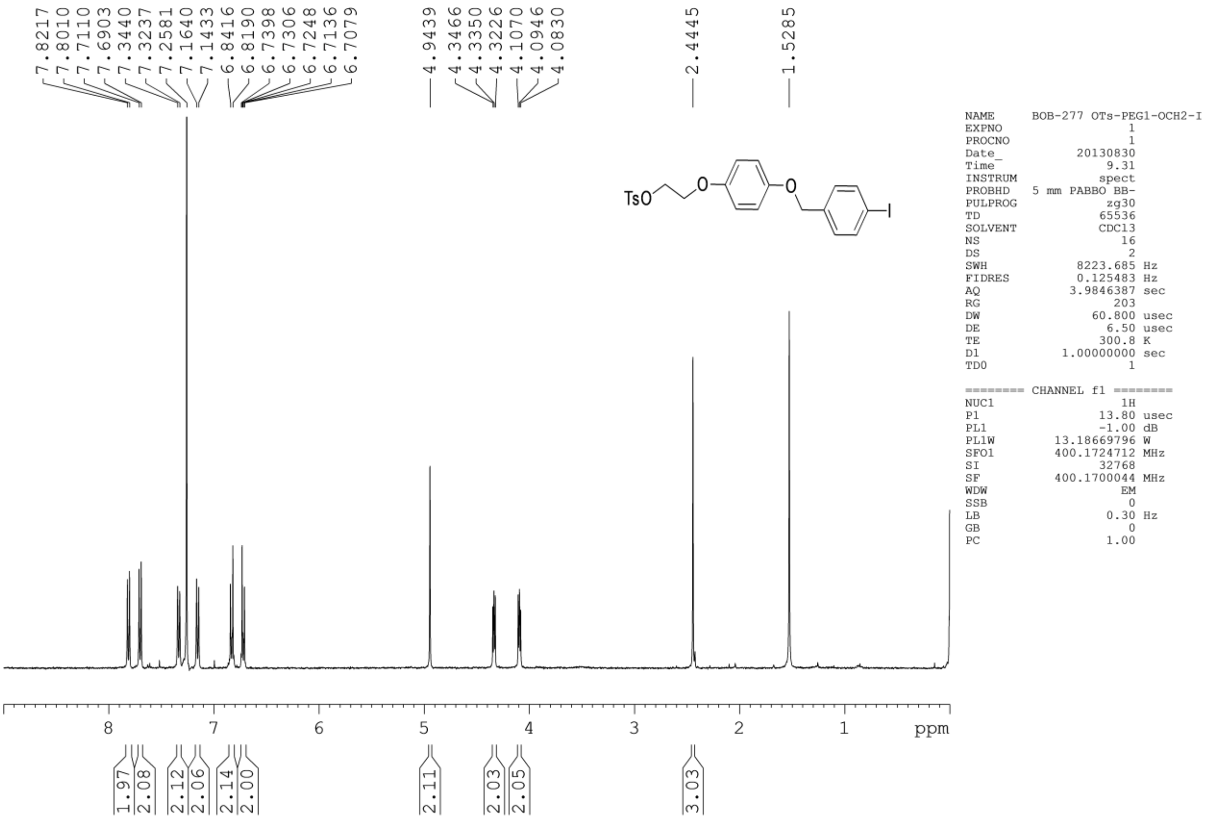

^{1}H NMR ($CDCl_3$) spectrum of 2-(4-((4-iodobenzyl)oxy)phenoxy)ethyl 4-methylbenzenesulfonate (**8a**)

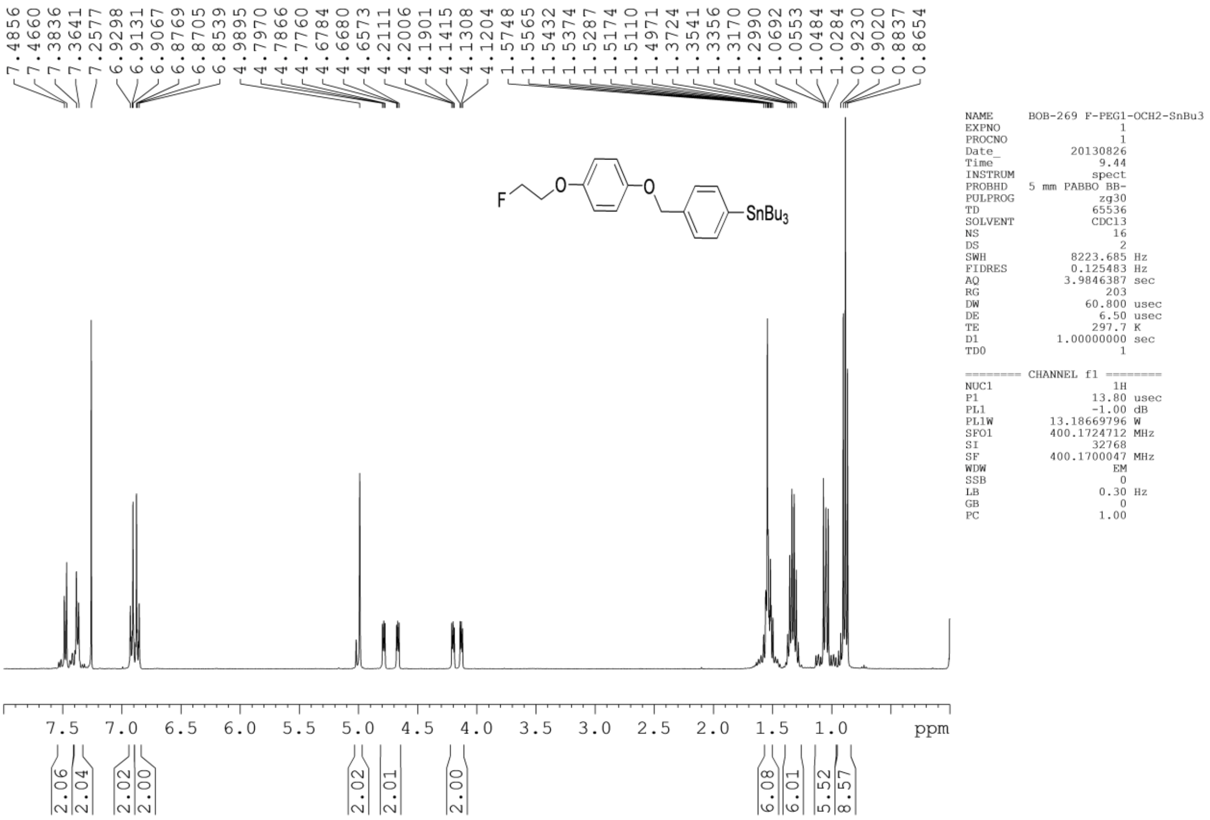

^{1}H NMR ($CDCl_3$) spectrum of tributyl(4-((4-(2-fluoroethoxy)phenoxy)methyl)phenyl)stannane (**8b**)

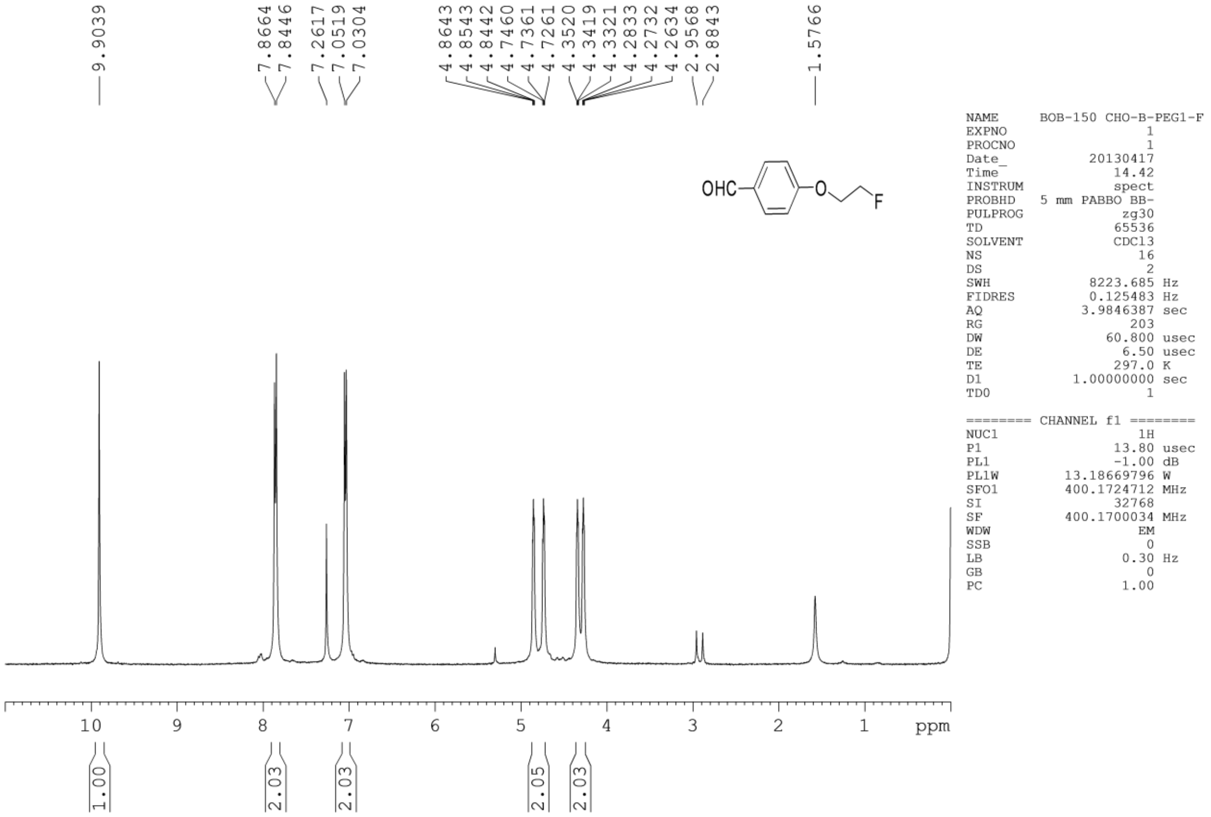

^{1}H NMR ($CDCl_3$) spectrum of 4-(2-fluoroethoxy)benzaldehyde (**9a**)

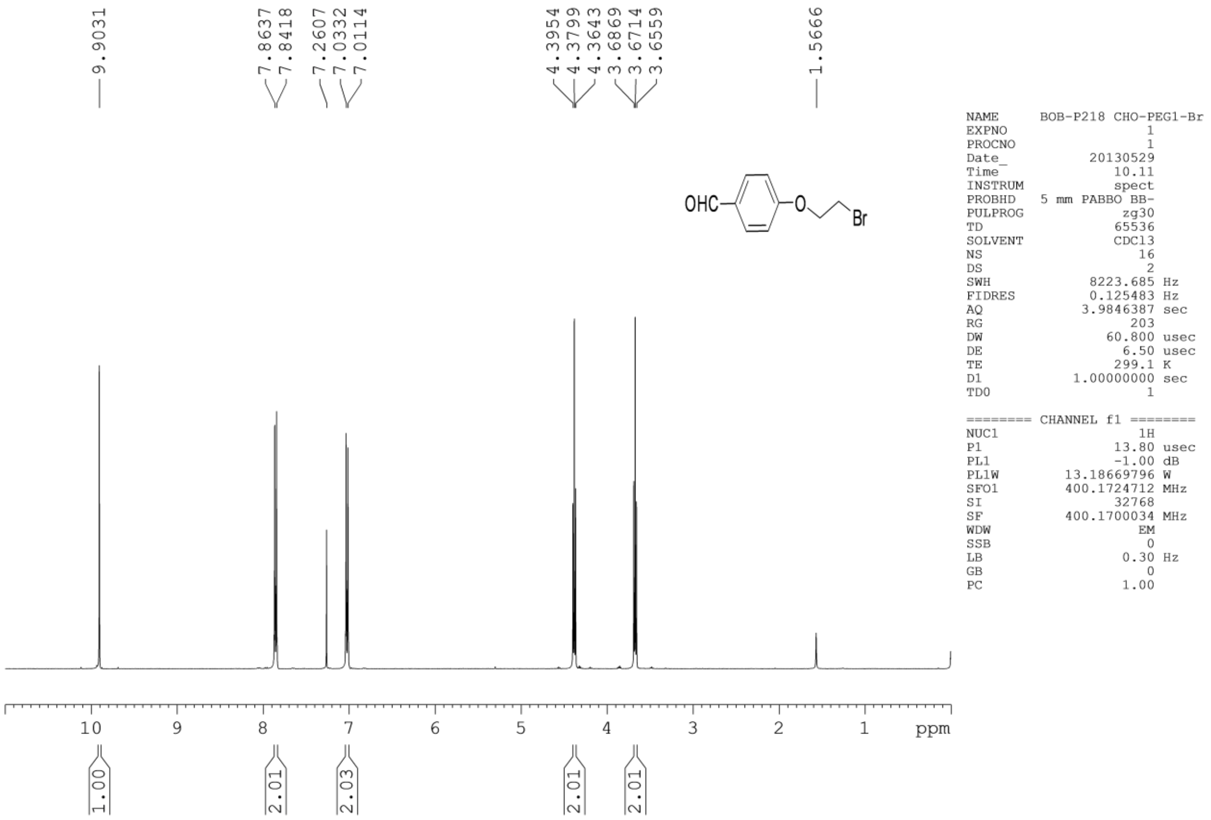

^{1}H NMR ($CDCl_3$) spectrum of 4-(2-bromoethoxy)benzaldehyde (**9b**)

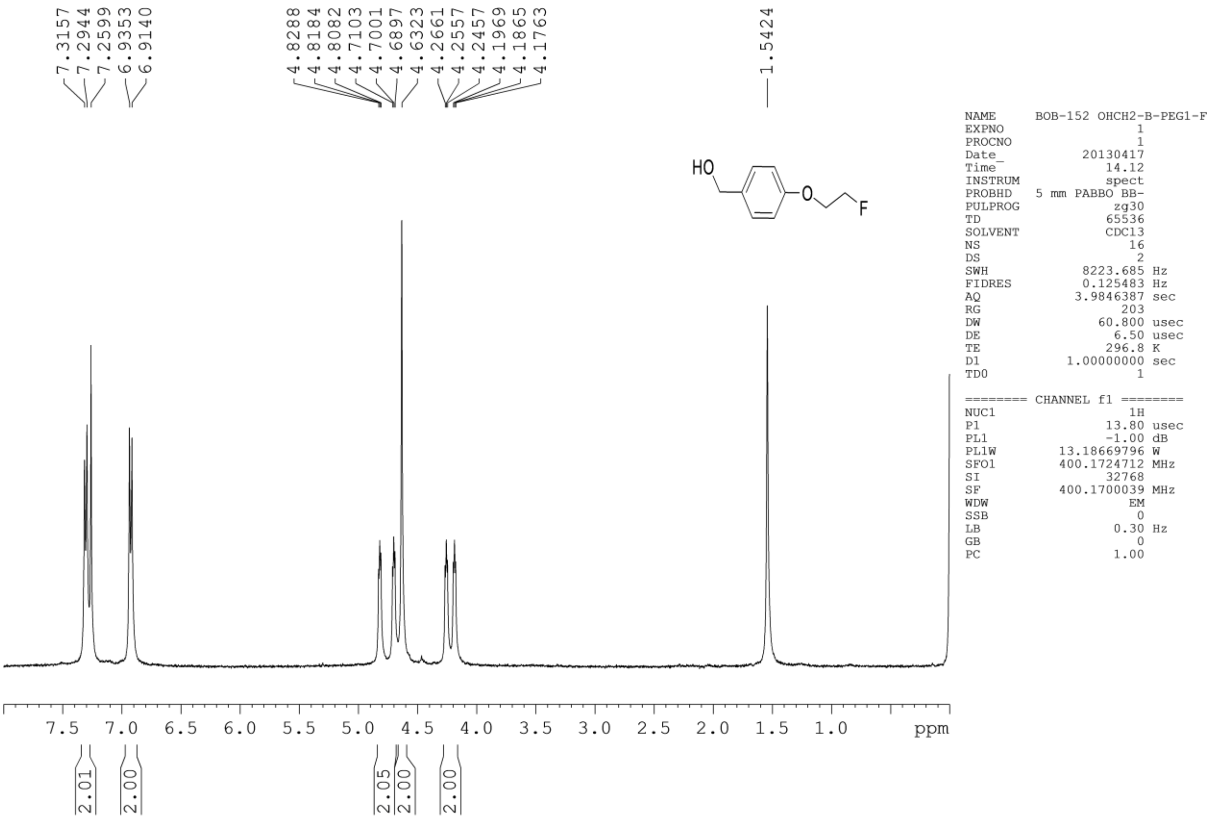

^{1}H NMR ($CDCl_3$) spectrum of (4-(2-fluoroethoxy)phenyl)methanol (**10a**)

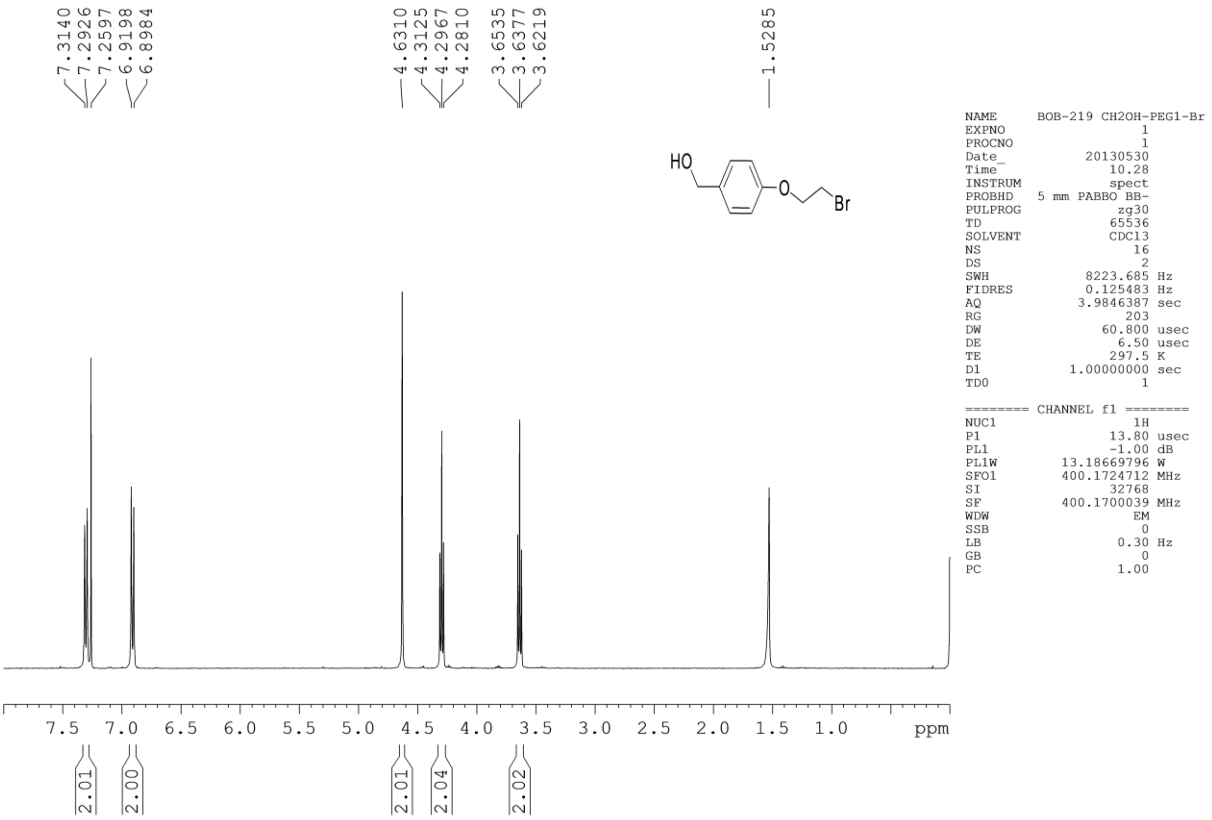

^{1}H NMR ($CDCl_3$) spectrum of (4-(2-bromoethoxy)phenyl)methanol (**10b**)

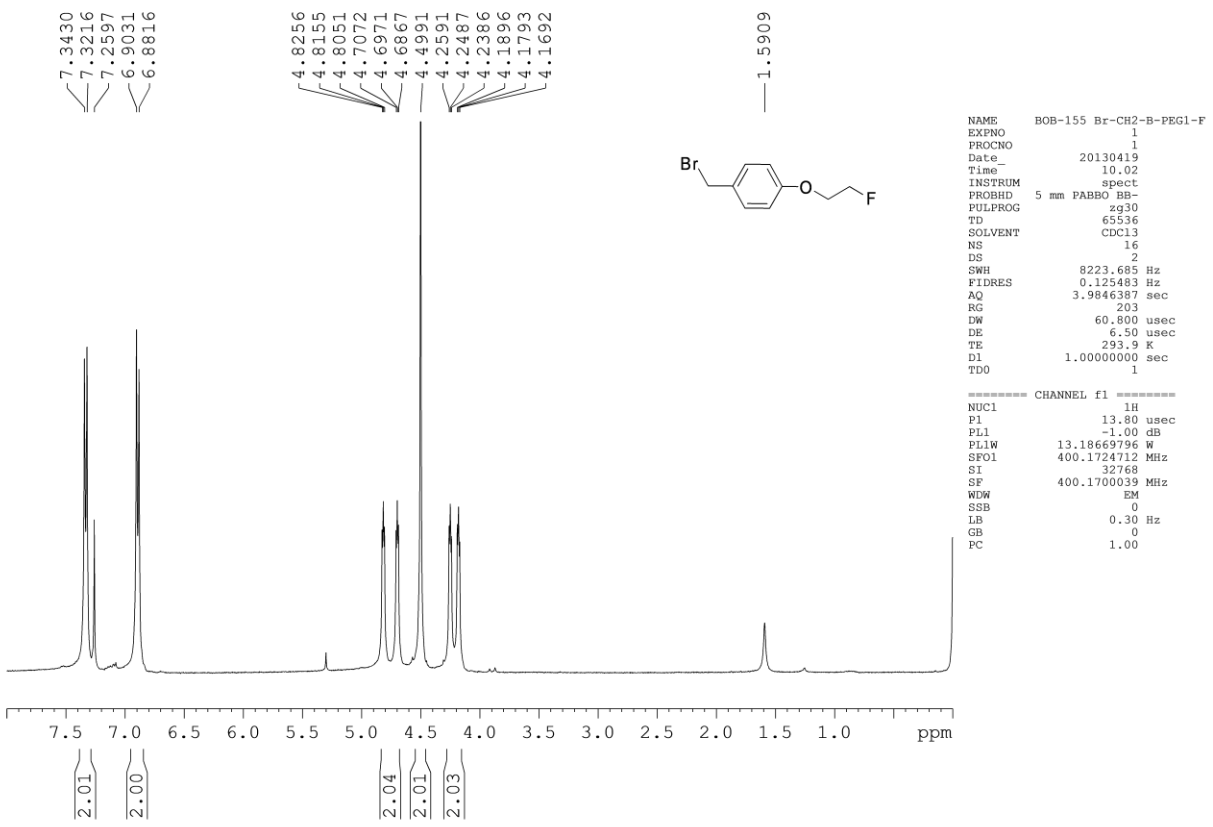

^{1}H NMR ($CDCl_3$) spectrum of 1-(bromomethyl)-4-(2-fluoroethoxy)benzene (**11a**)

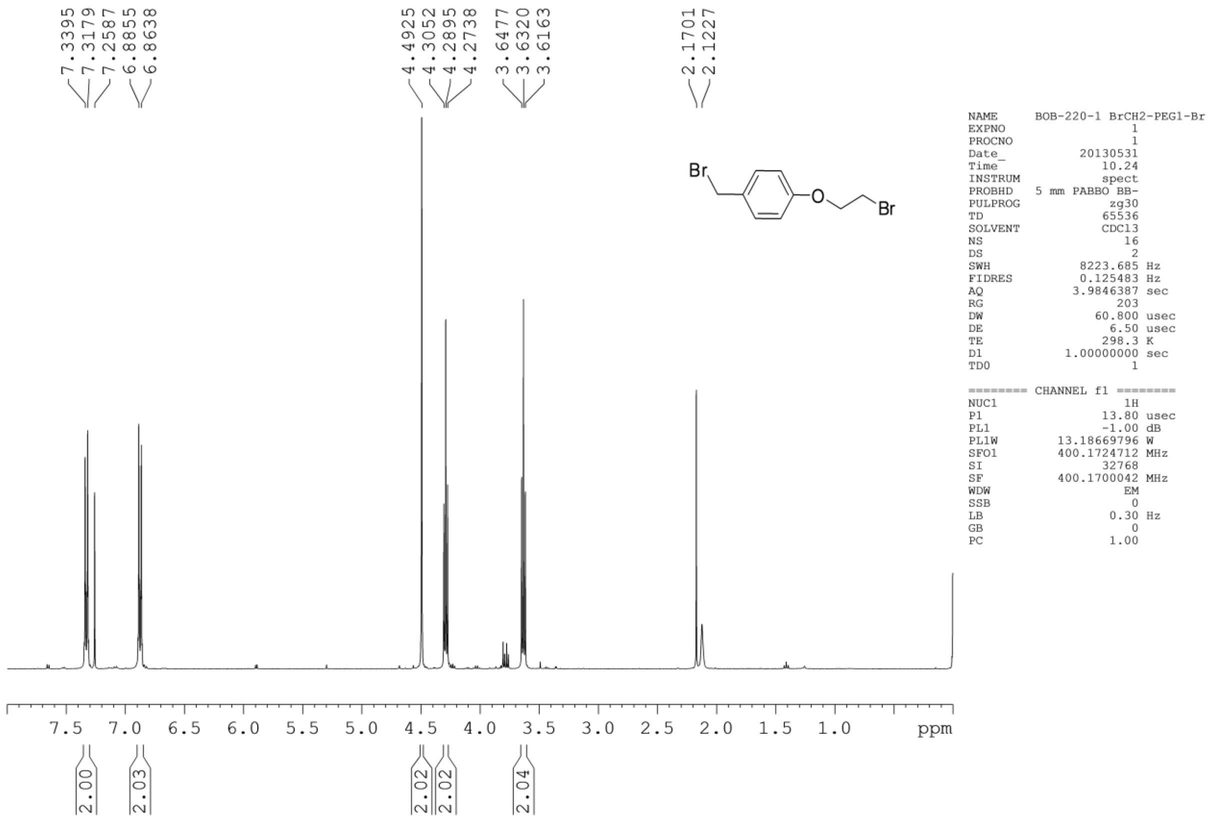

^{1}H NMR ($CDCl_3$) spectrum of 1-(2-bromoethoxy)-4-(bromomethyl)benzene (**11b**)

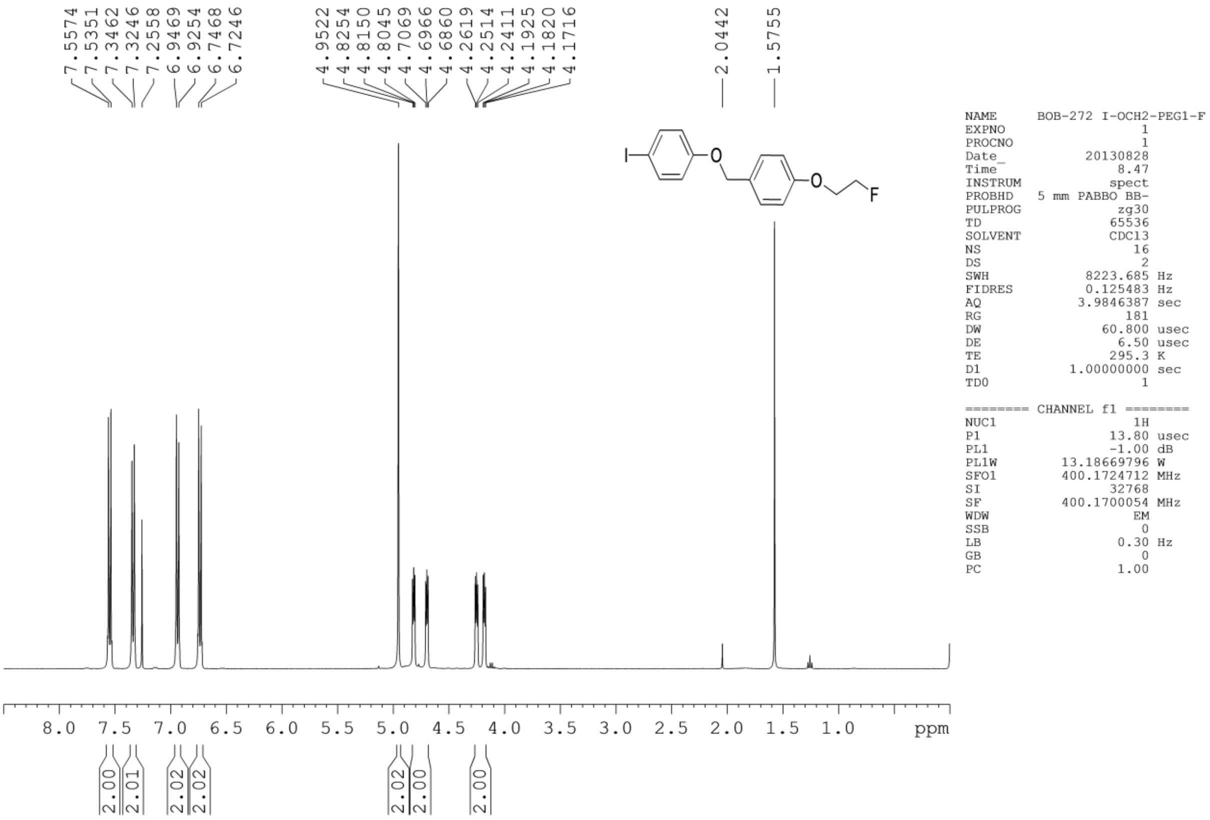

^{1}H NMR ($CDCl_3$) spectrum of 1-(2-fluoroethoxy)-4-((4-iodophenoxy)methyl)benzene (**12a**)

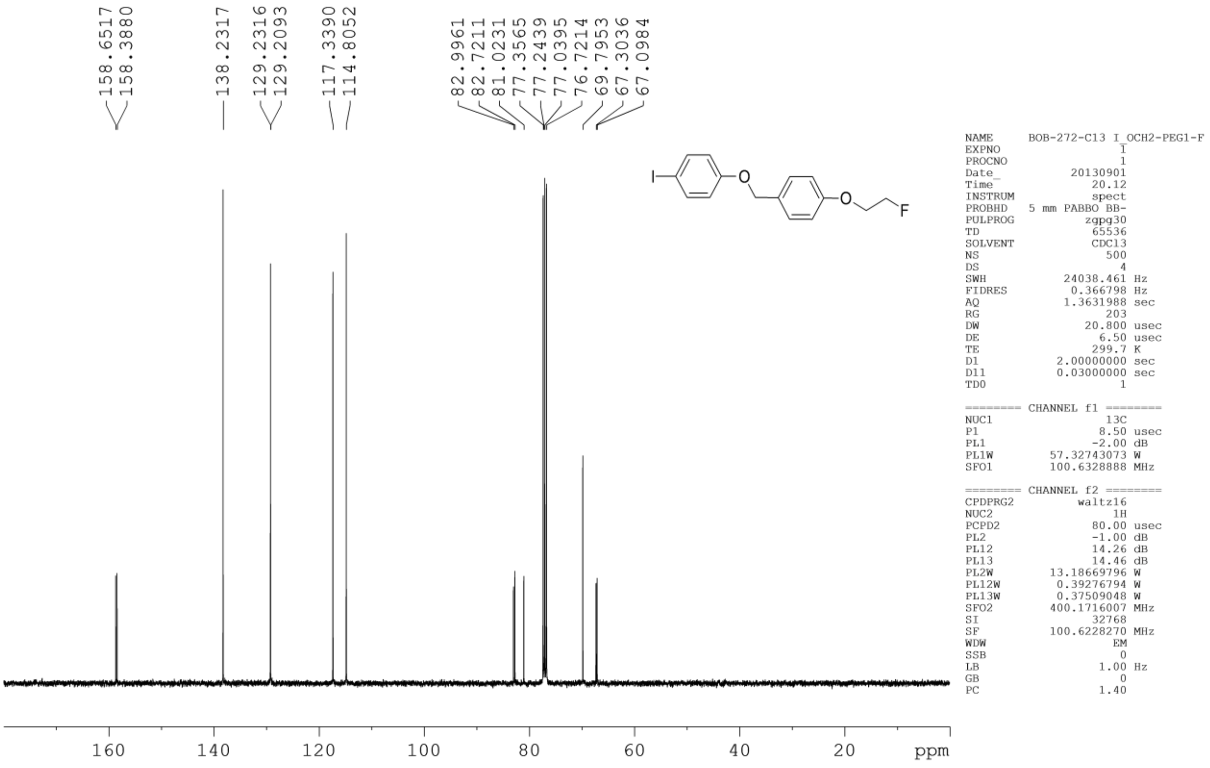

^{13}C NMR ($CDCl_3$) spectrum of 1-(2-fluoroethoxy)-4-((4-iodophenoxy)methyl)benzene (**12a**)

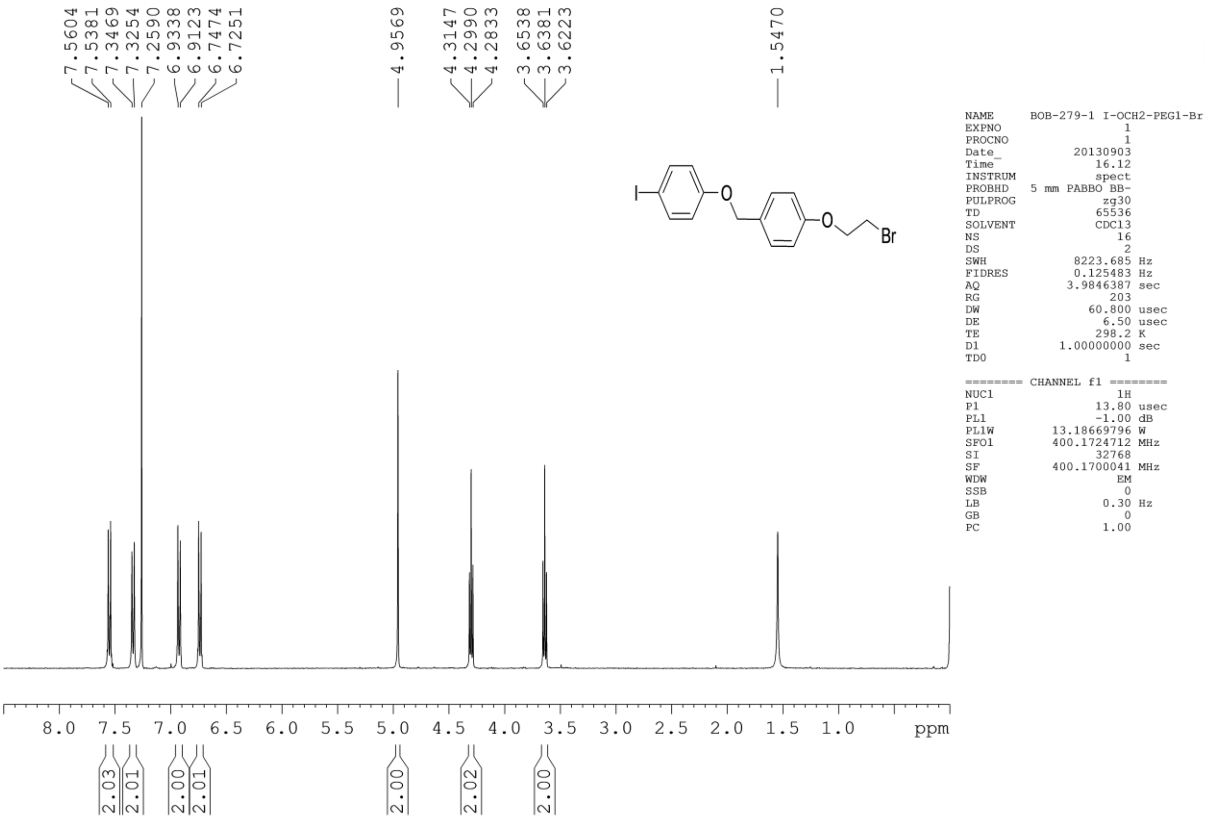

^{1}H NMR ($CDCl_3$) spectrum of 1-(2-bromoethoxy)-4-((4-iodophenoxy)methyl)benzene (**12b**)

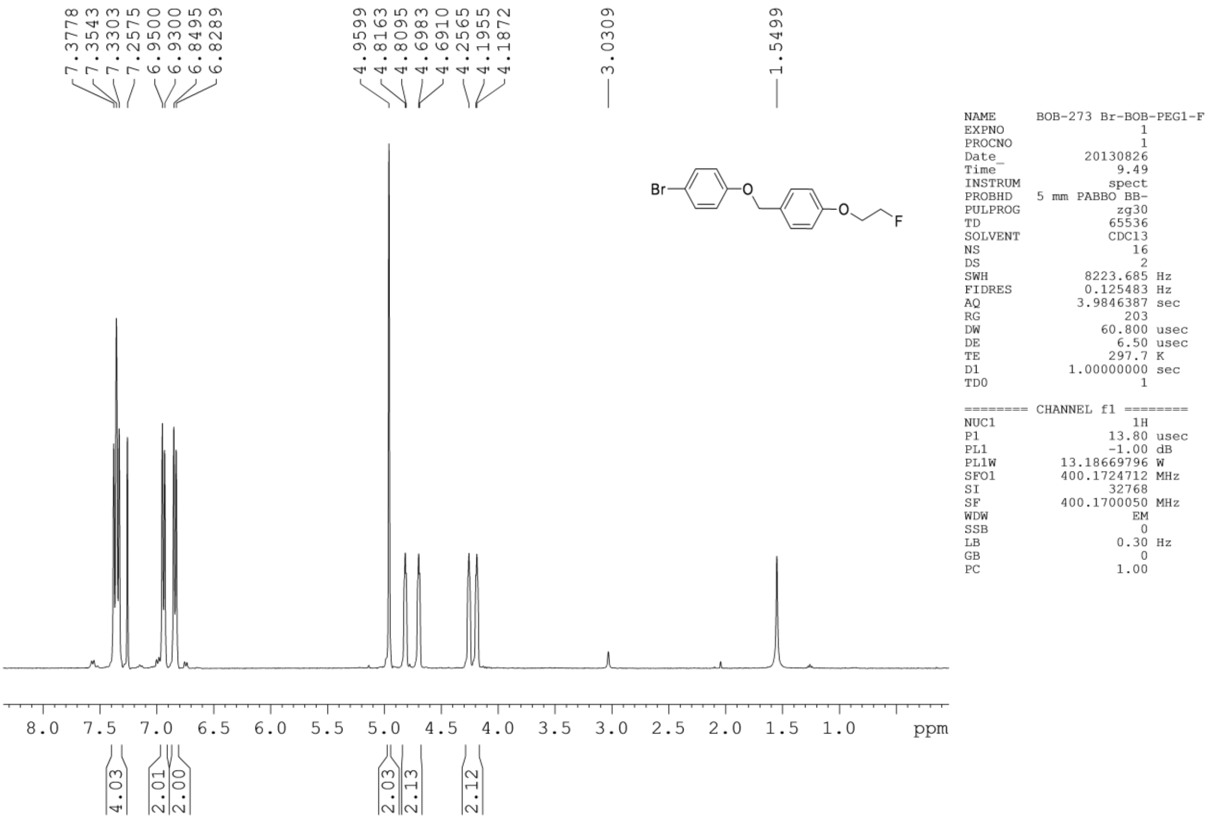

^{1}H NMR ($CDCl_3$) spectrum of 1-bromo-4-((4-(2-fluoroethoxy)benzyl)oxy)benzene (**12c**)

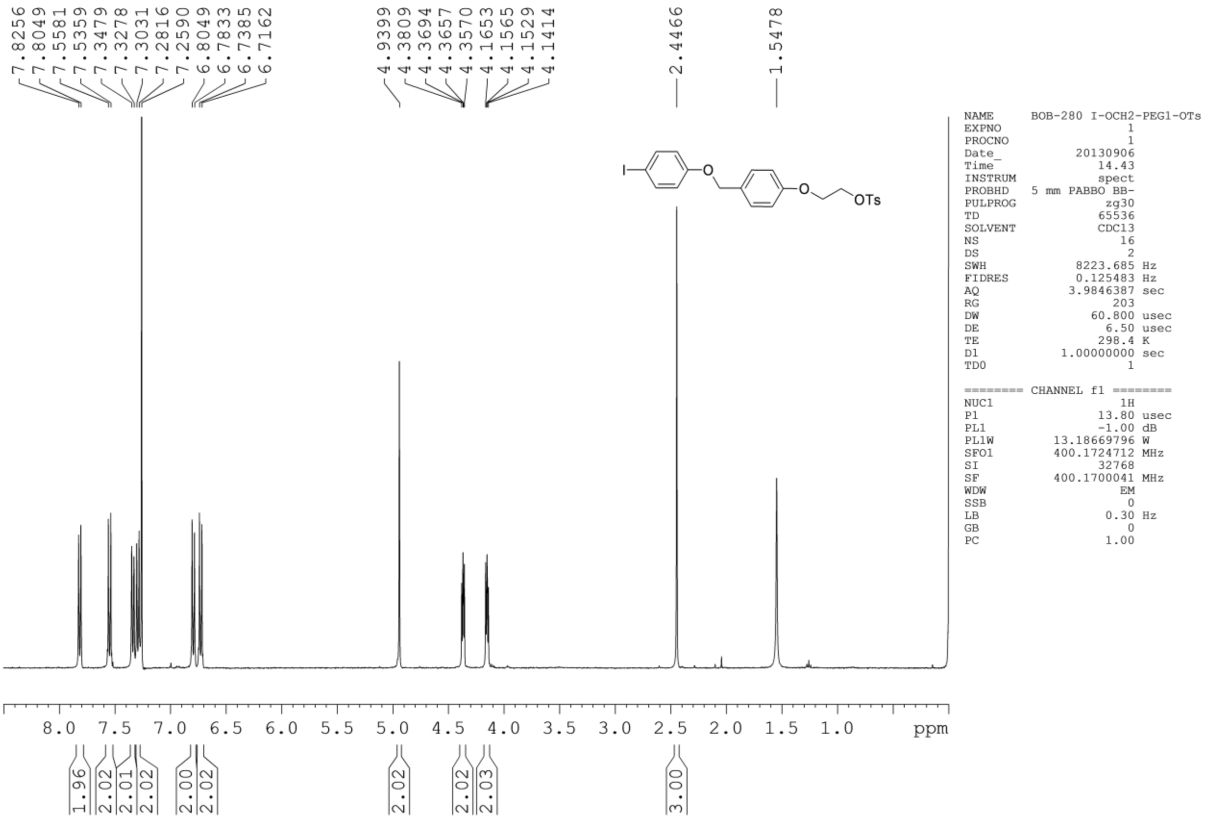

^{1}H NMR ($CDCl_3$) spectrum of 2-(4-((4-iodophenoxy)methyl)phenoxy)ethyl 4-methylbenzenesulfonate (**13a**)

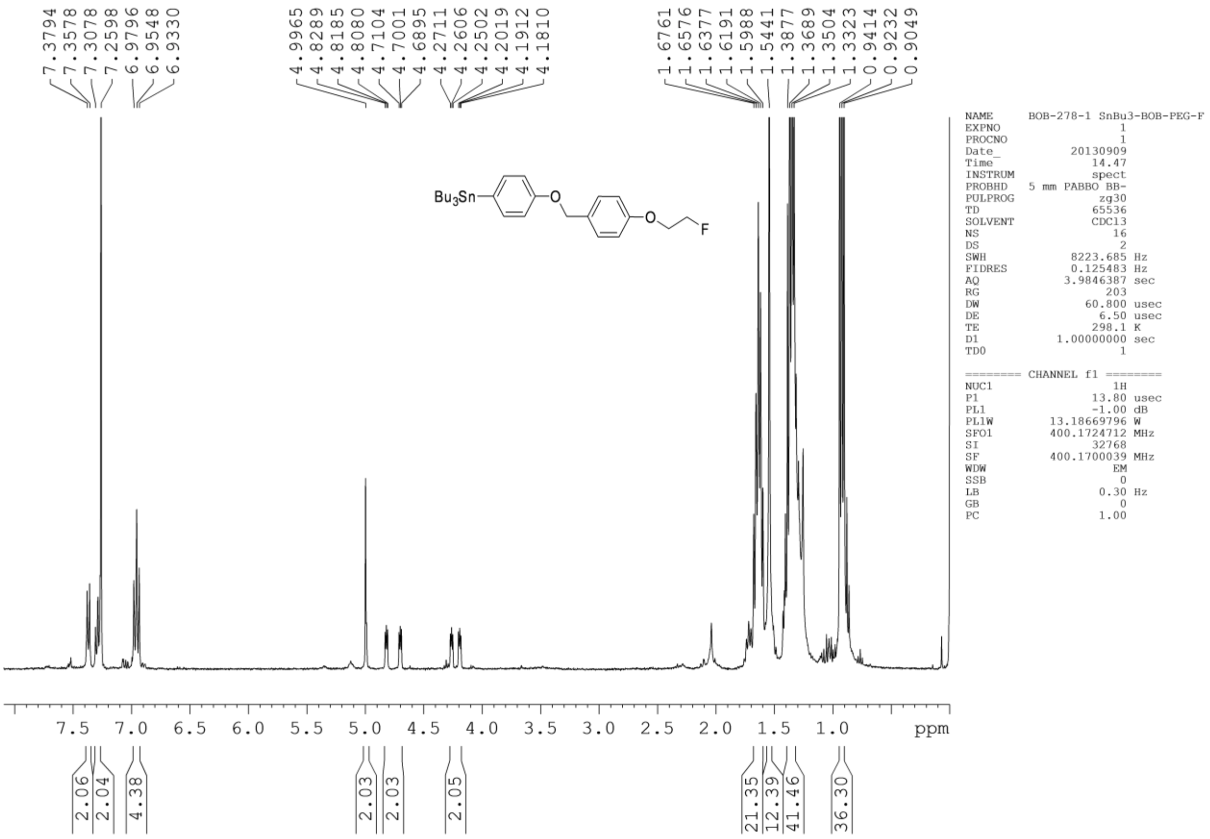

^{1}H NMR ($CDCl_3$) spectrum of tributyl(4-((4-(2-fluoroethoxy)benzyl)oxy)phenyl)stannane (**13b**)

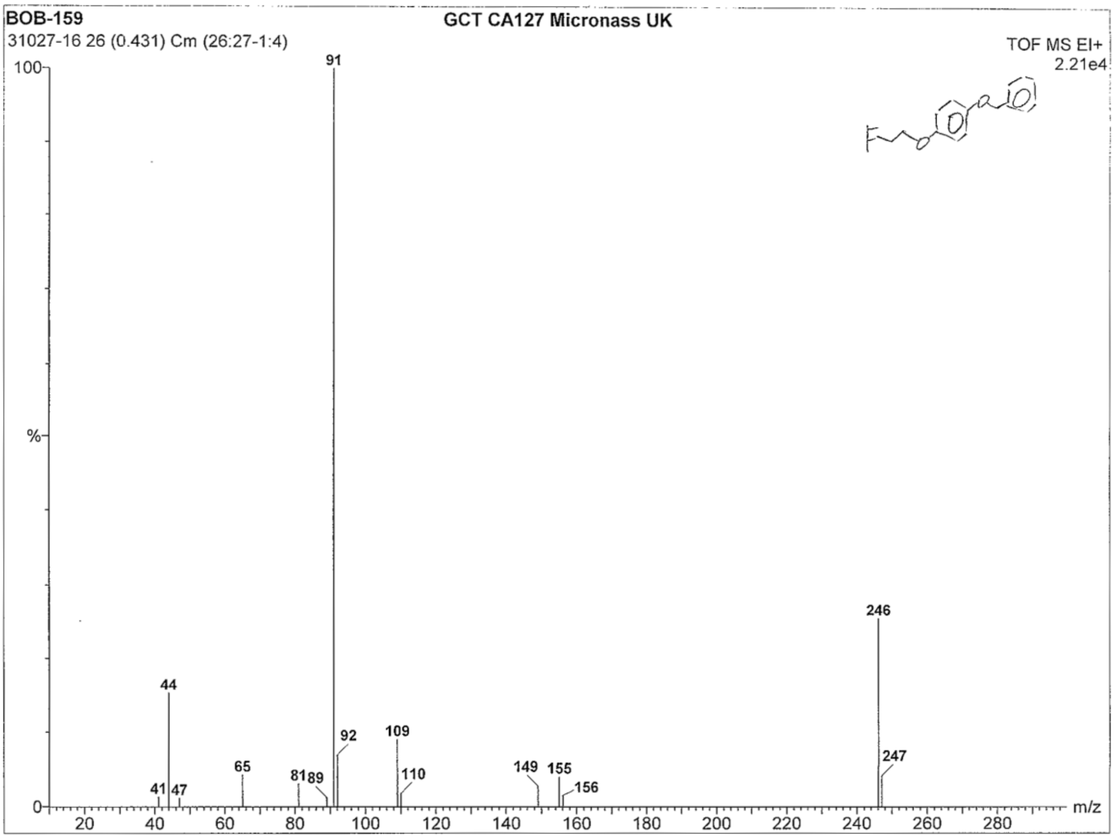

MS spectrum of 1-(benzyloxy)-4-(2-fluoroethoxy)benzene (**5a**)

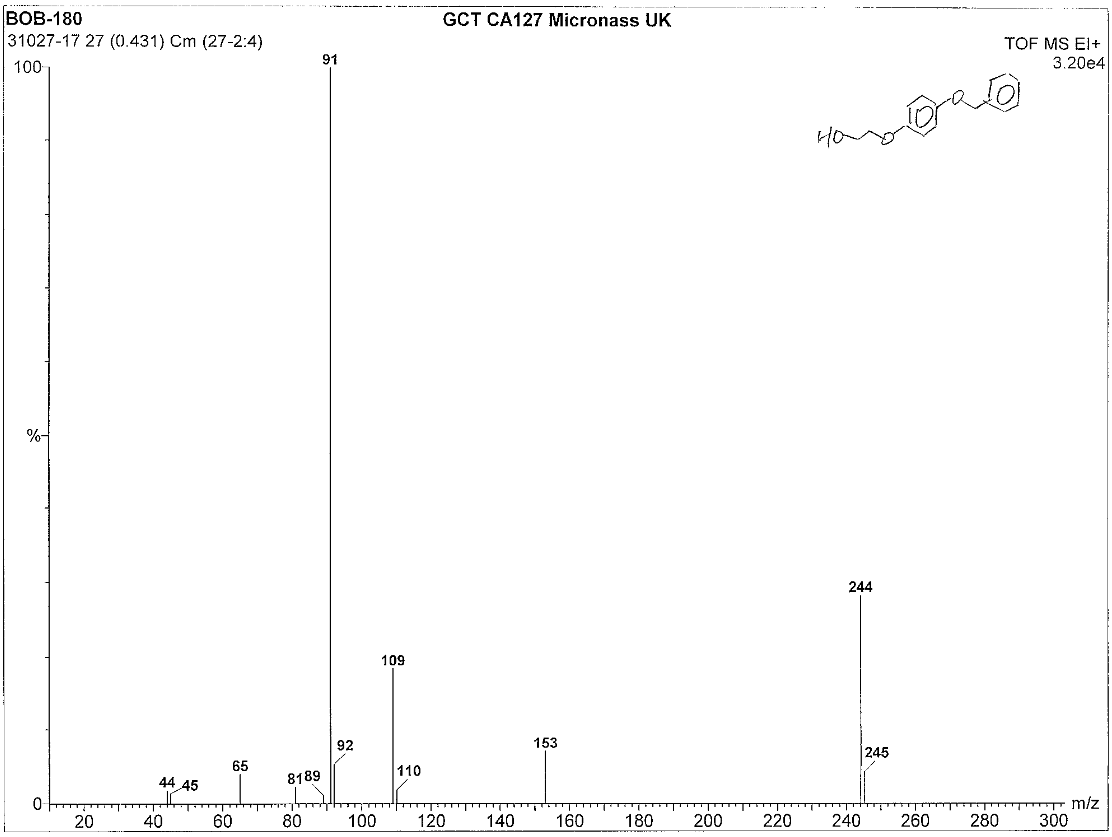

MS spectrum of 2-(4-(benzyloxy)phenoxy)ethanol (**5b**)

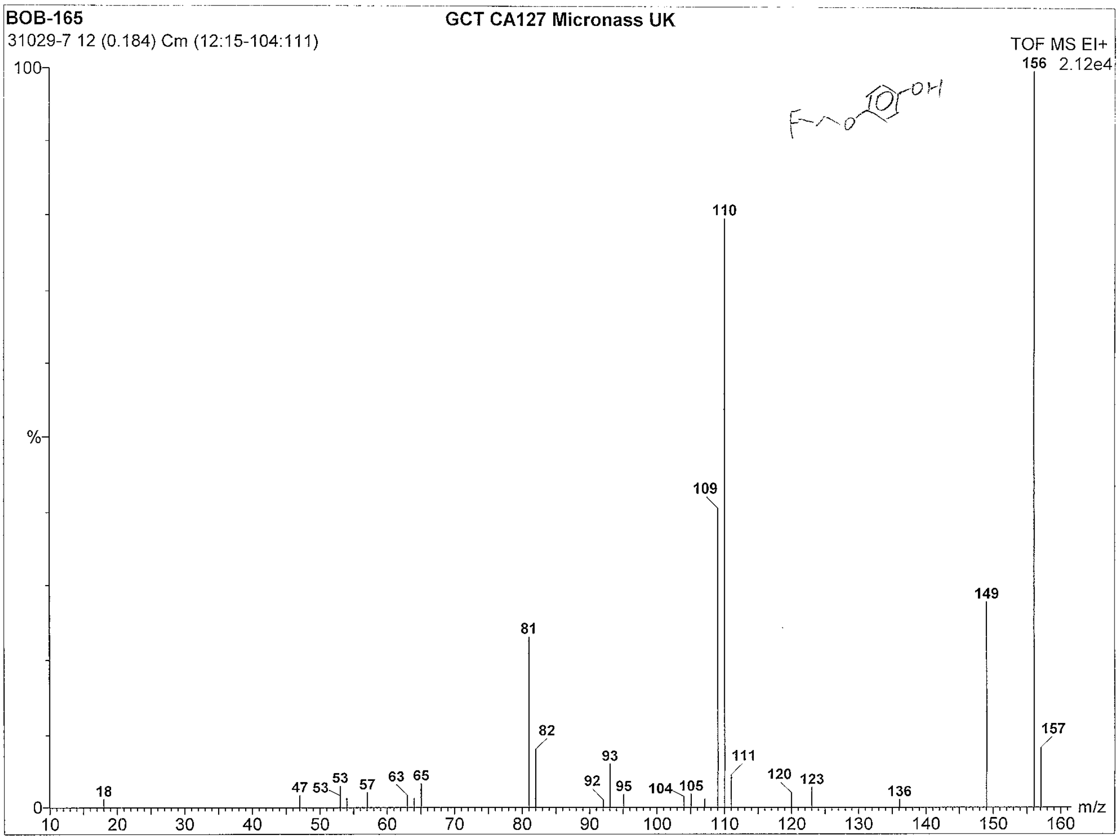

MS spectrum of 4-(2-fluoroethoxy)phenol (**6a**)

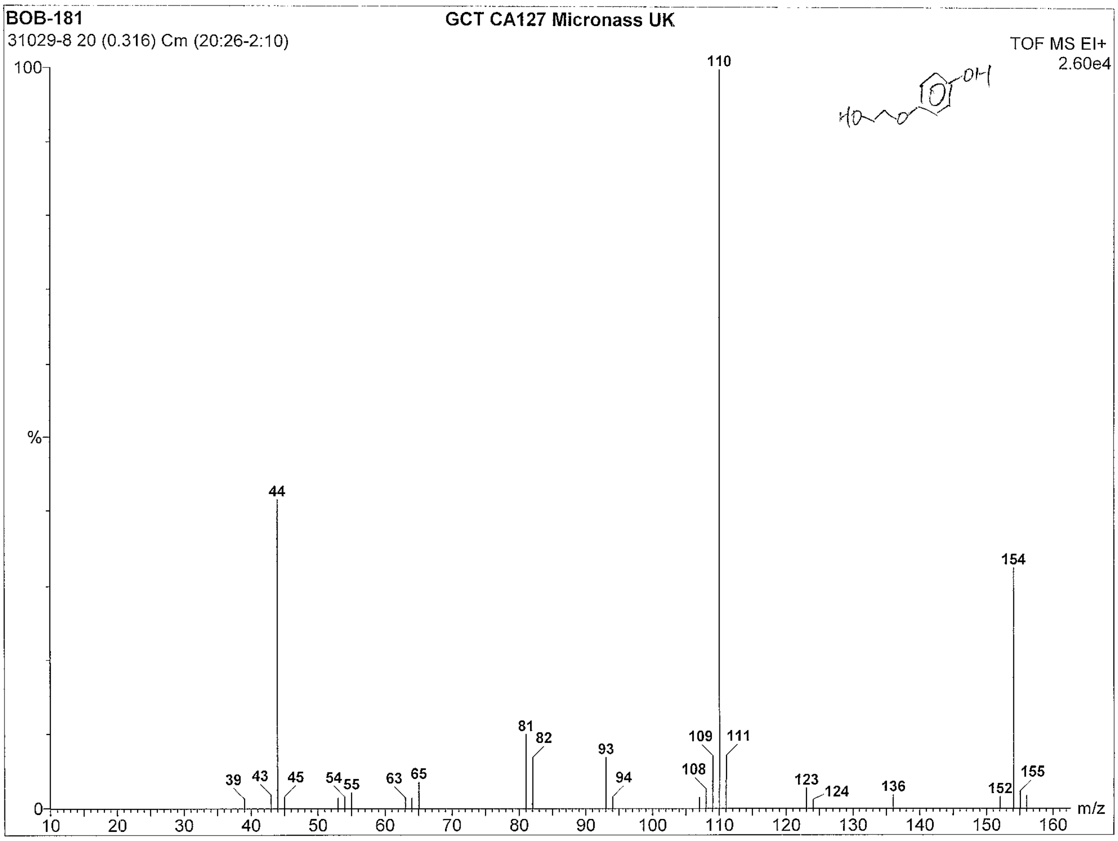

MS spectrum of 4-(2-hydroxyethoxy)phenol (**6b**)

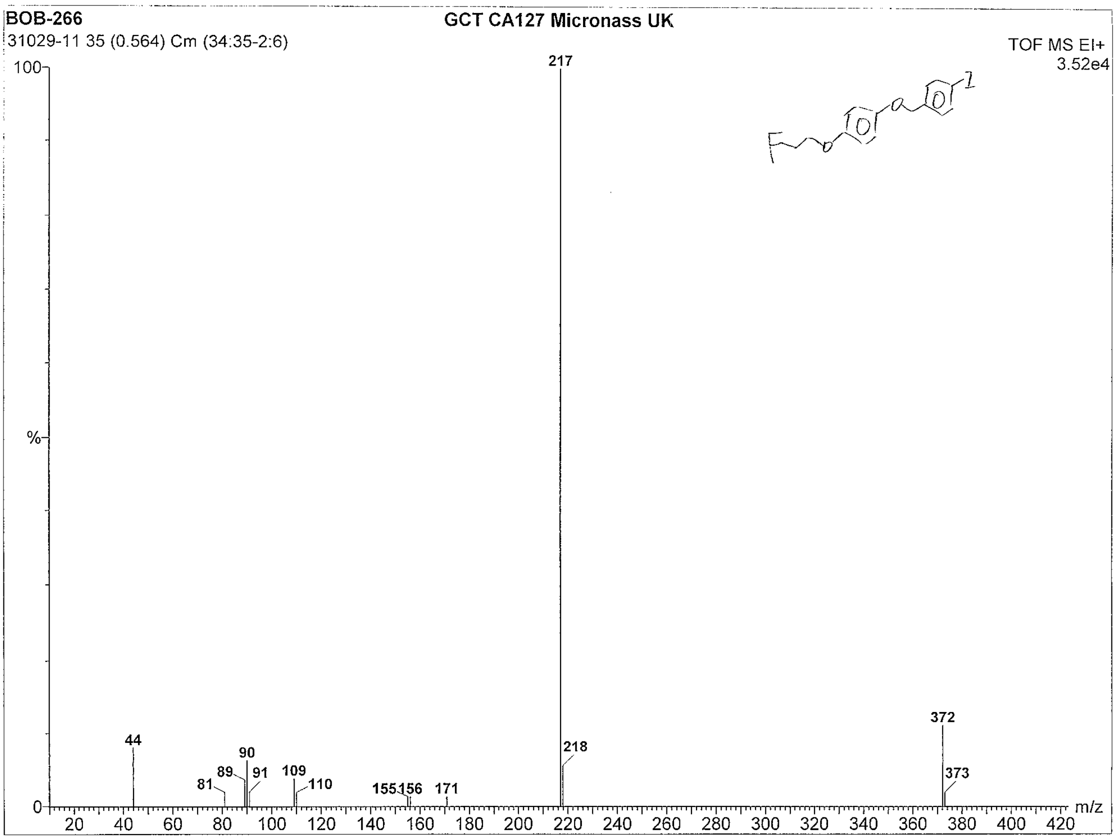

MS spectrum of 1-(2-fluoroethoxy)-4-((4-iodobenzyl)oxy)benzene (**7a**)

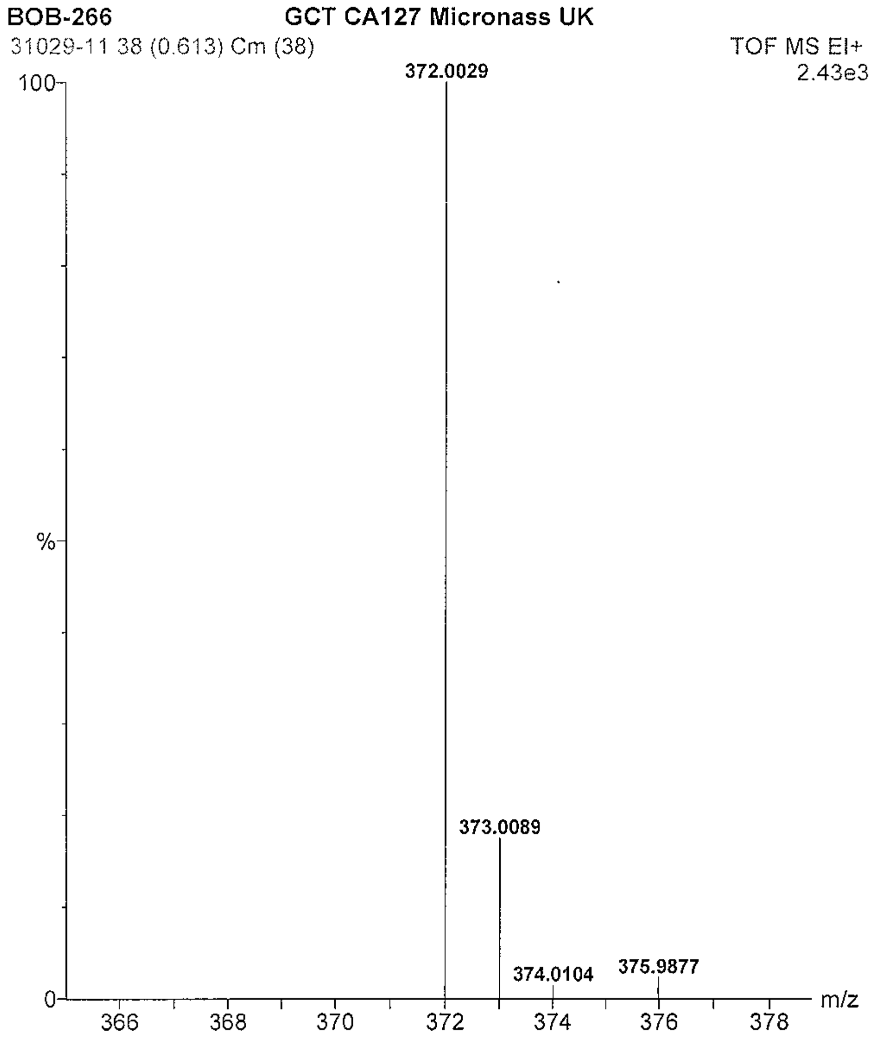

Elemental Composition Report

Tolerance = 10.0 PPM / DBE: min = -1.5, max = 50.0
Isotope cluster parameters: Separation = 1.0 Abundance = 1.0%

Monoisotopic Mass, Odd and Even Electron Ions
87 formula(e) evaluated with 3 results within limits (up to 50 closest results for each mass)

Minimum:	80.00				-1.5		
Maximum:	100.00		200.0	10.0	50.0		
Mass	RA	Calc. Mass	mDa	PPM	DBE	Score	Formula
372.0029	100.00	372.0023	0.6	1.7	8.0	1	C15 H14 O2 F I

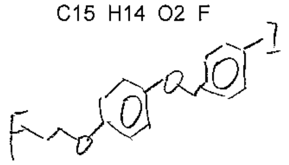

HRMS spectrum of 1-(2-fluoroethoxy)-4-((4-iodobenzyl)oxy)benzene (**7a**)

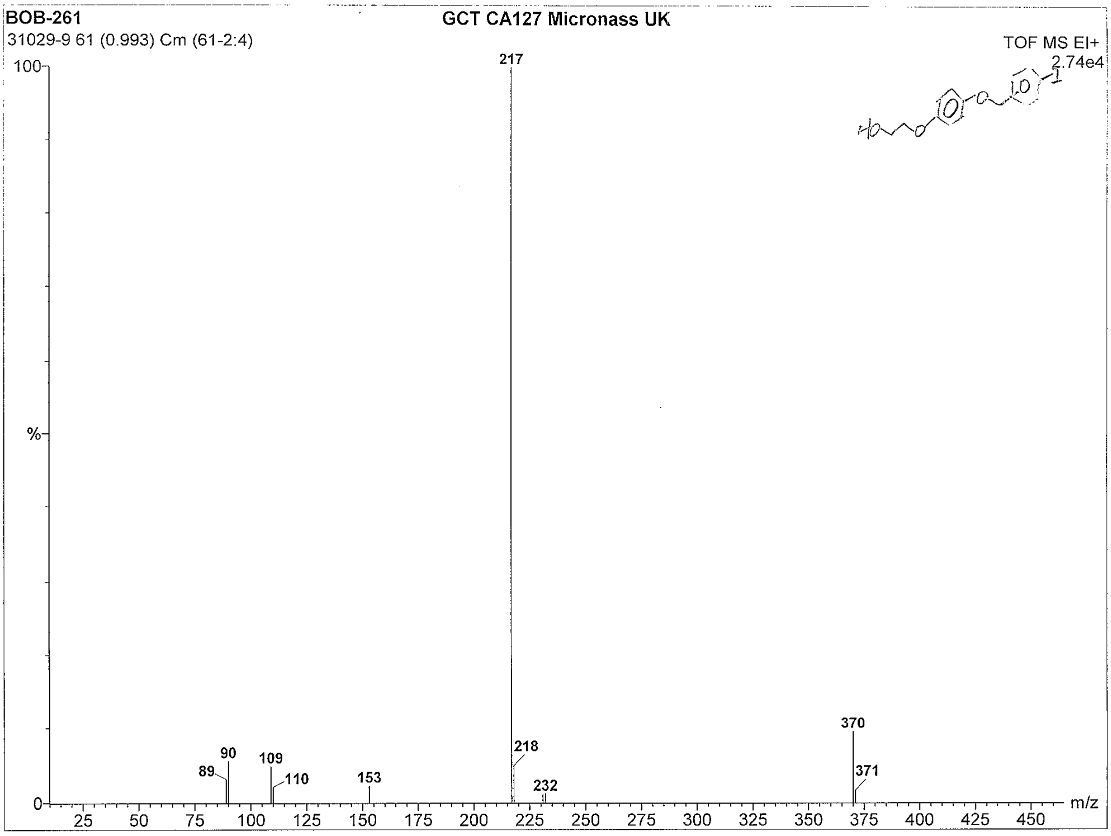

MS spectrum of 2-(4-((4-iodobenzyl)oxy)phenoxy)ethanol (**7b**)

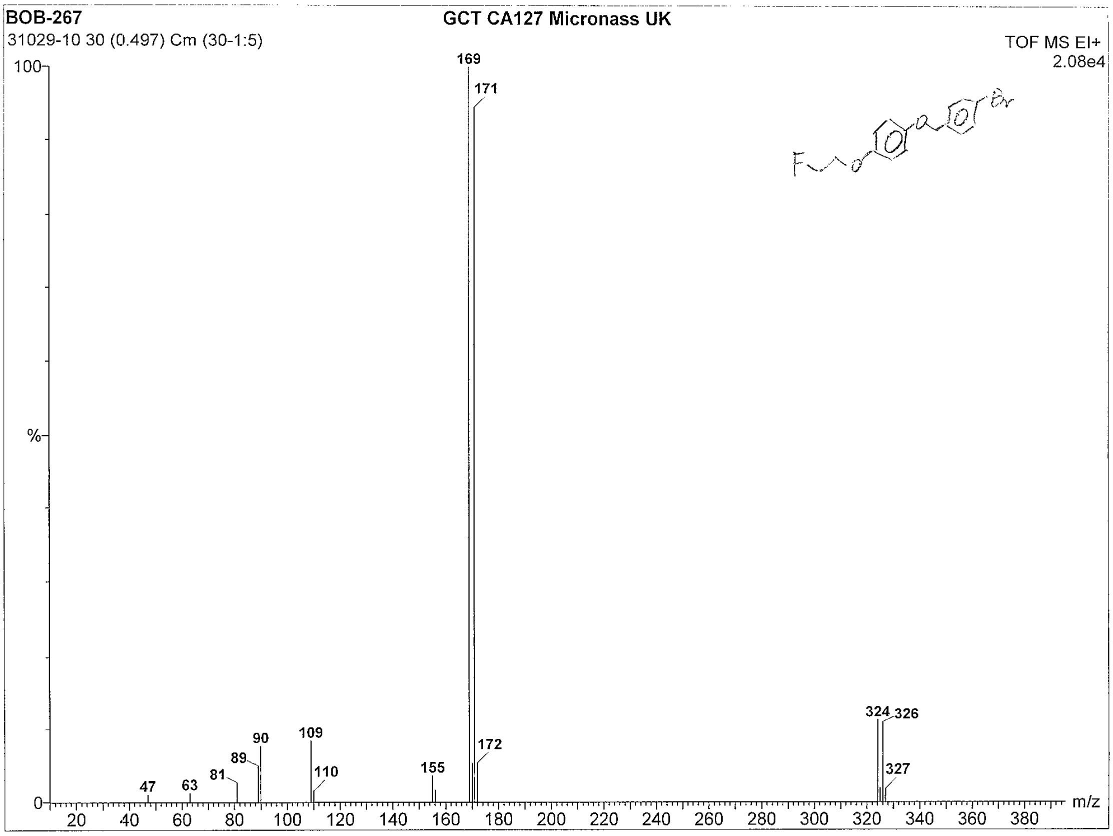

MS spectrum of 1-bromo-4-((4-(2-fluoroethoxy)phenoxy)methyl)benzene (**7c**)

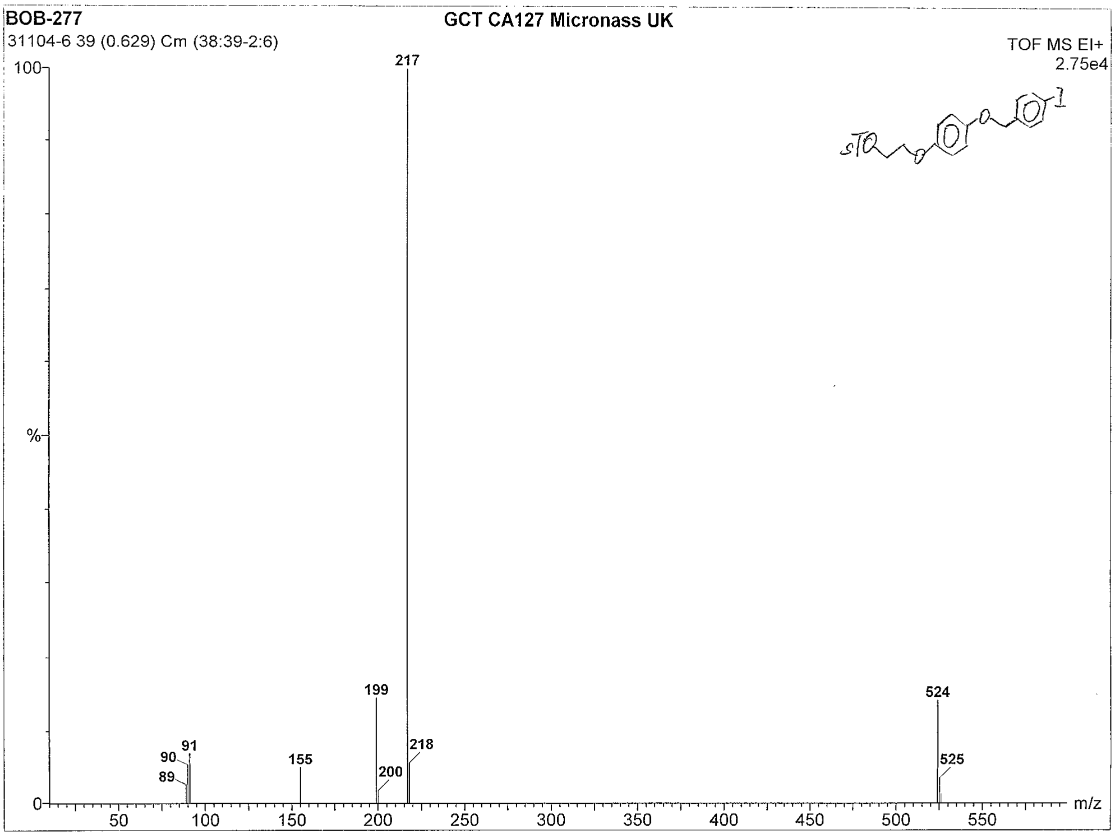

MS spectrum of 2-(4-((4-iodobenzyl)oxy)phenoxy)ethyl 4-methylbenzenesulfonate (**8a**)

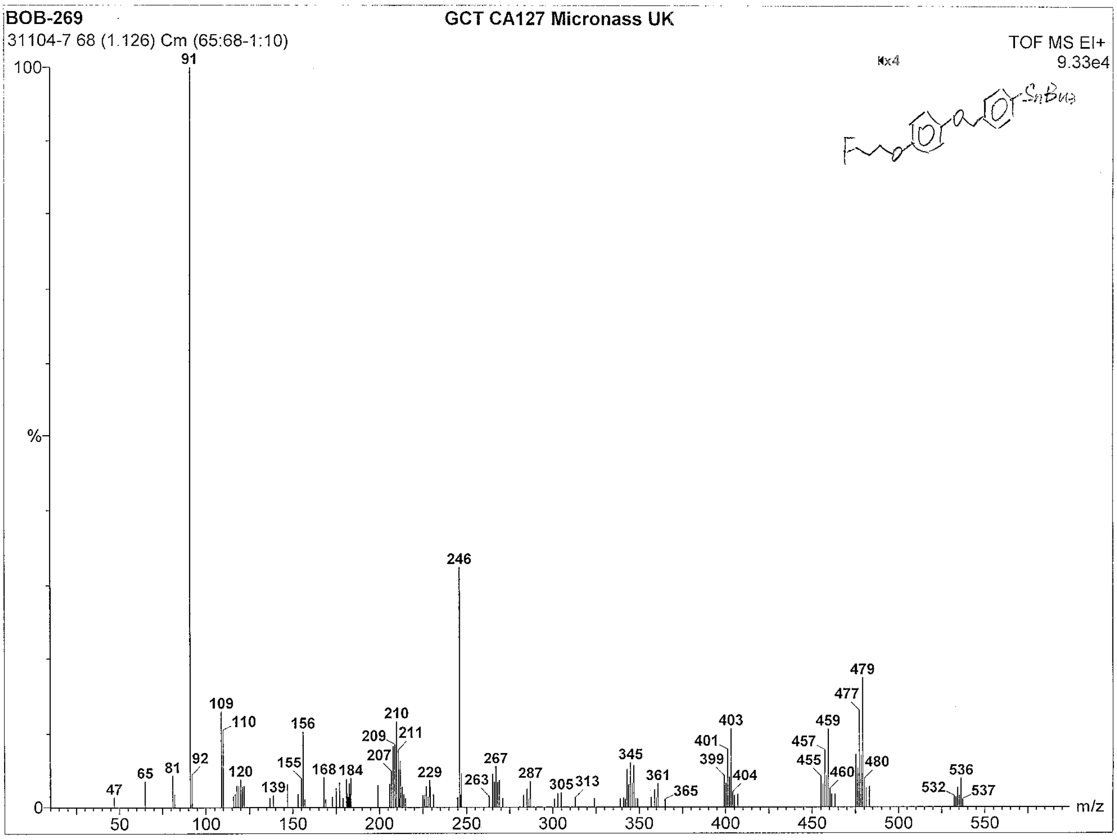

MS spectrum of tributyl(4-((4-(2-fluoroethoxy)phenoxy)methyl)phenyl)stannane (**8b**)

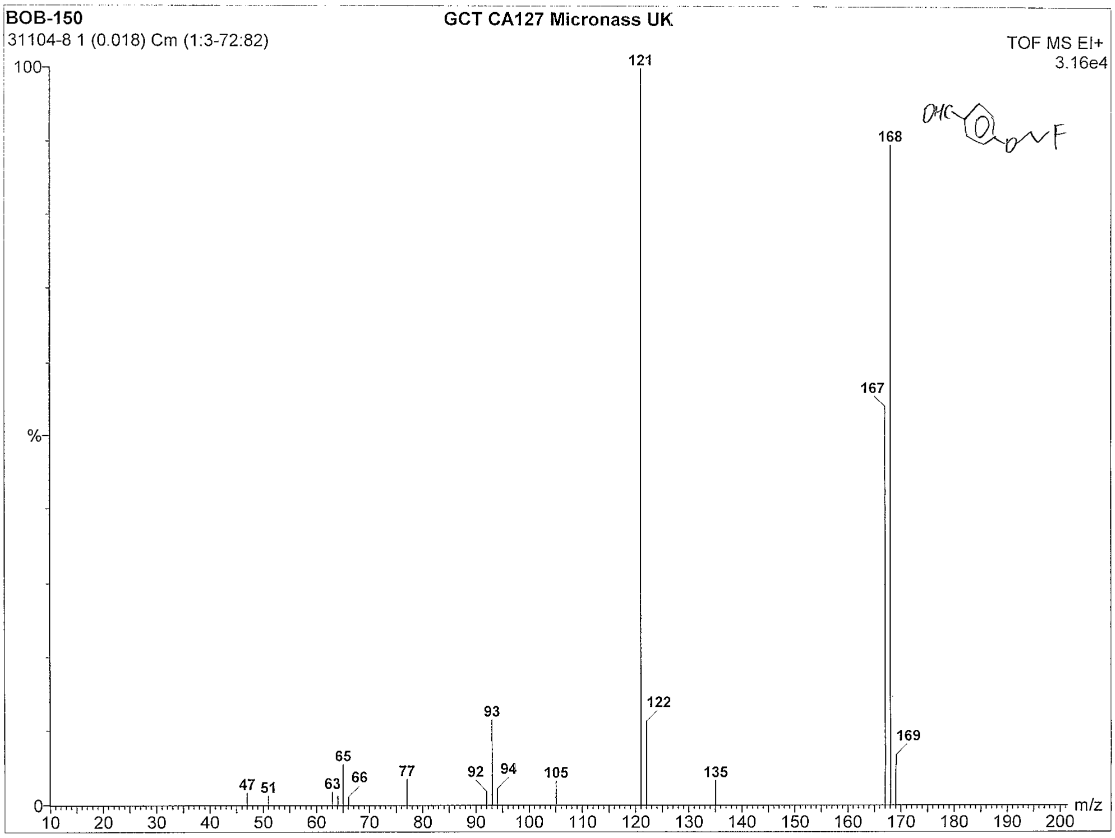

MS spectrum of 4-(2-fluoroethoxy)benzaldehyde (**9a**)

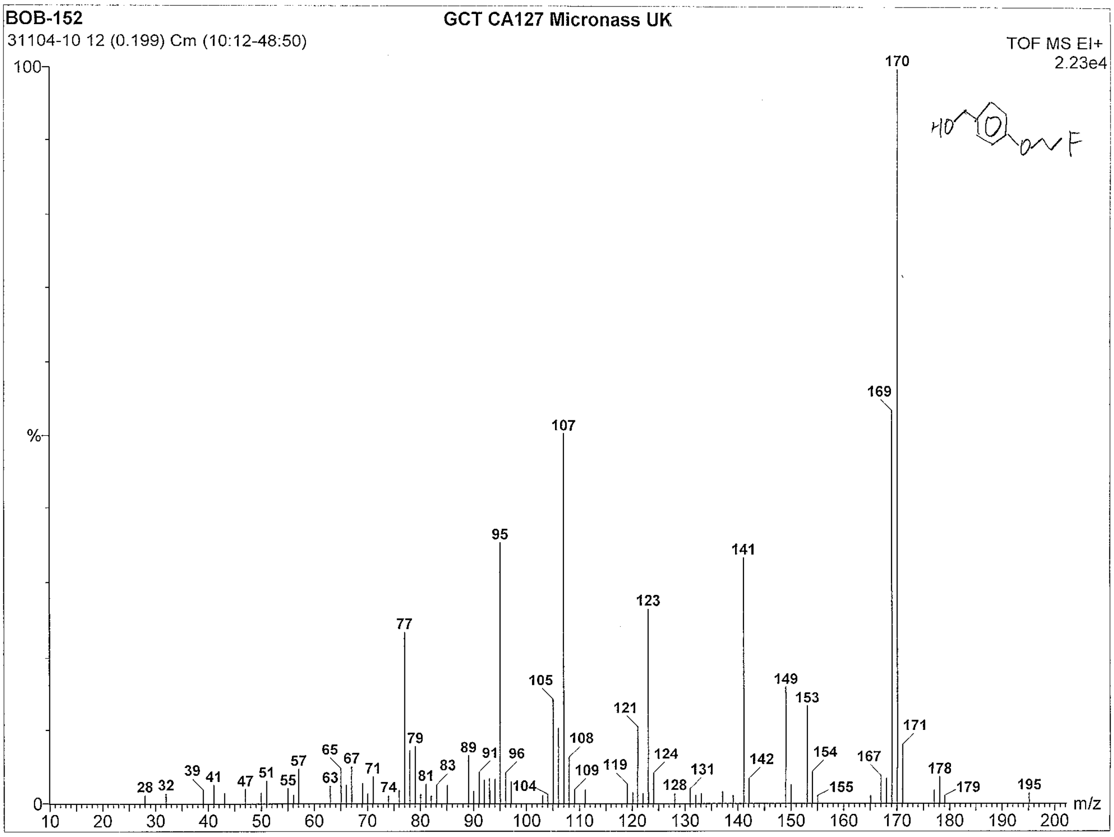

MS spectrum of (4-(2-fluoroethoxy)phenyl)methanol (**10a**)

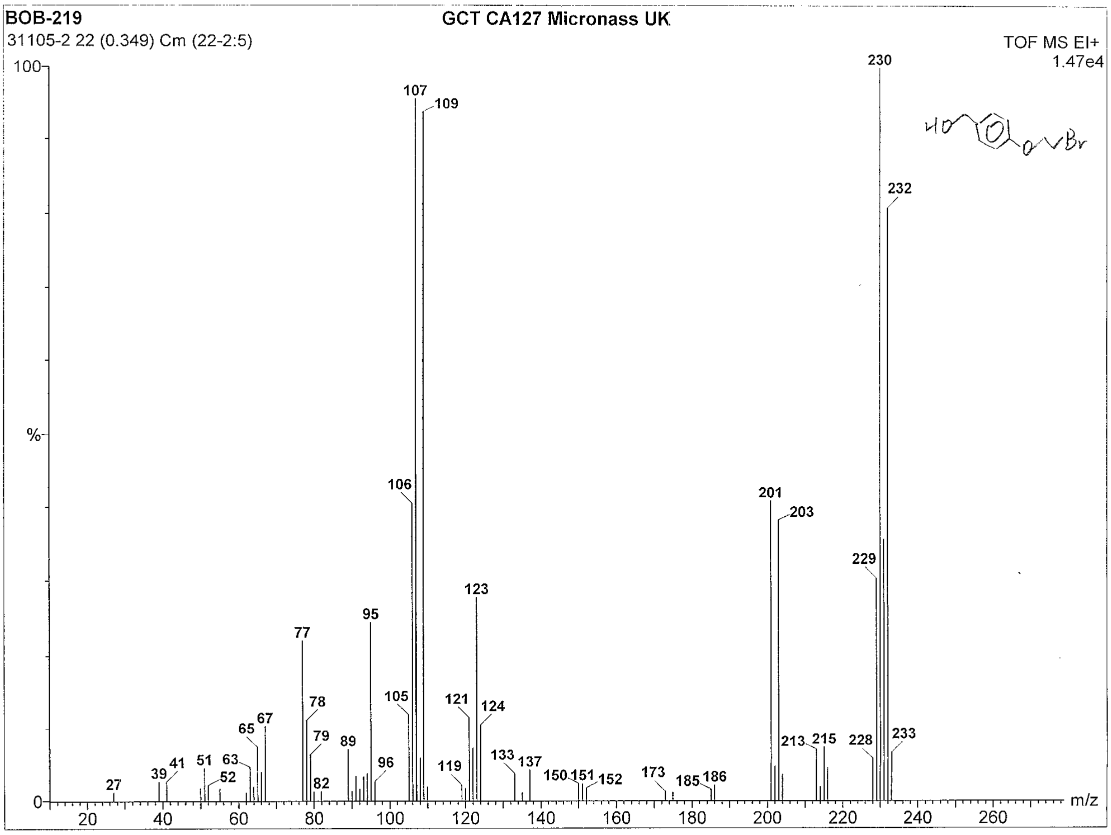

MS spectrum of (4-(2-bromoethoxy)phenyl)methanol (**10b**)

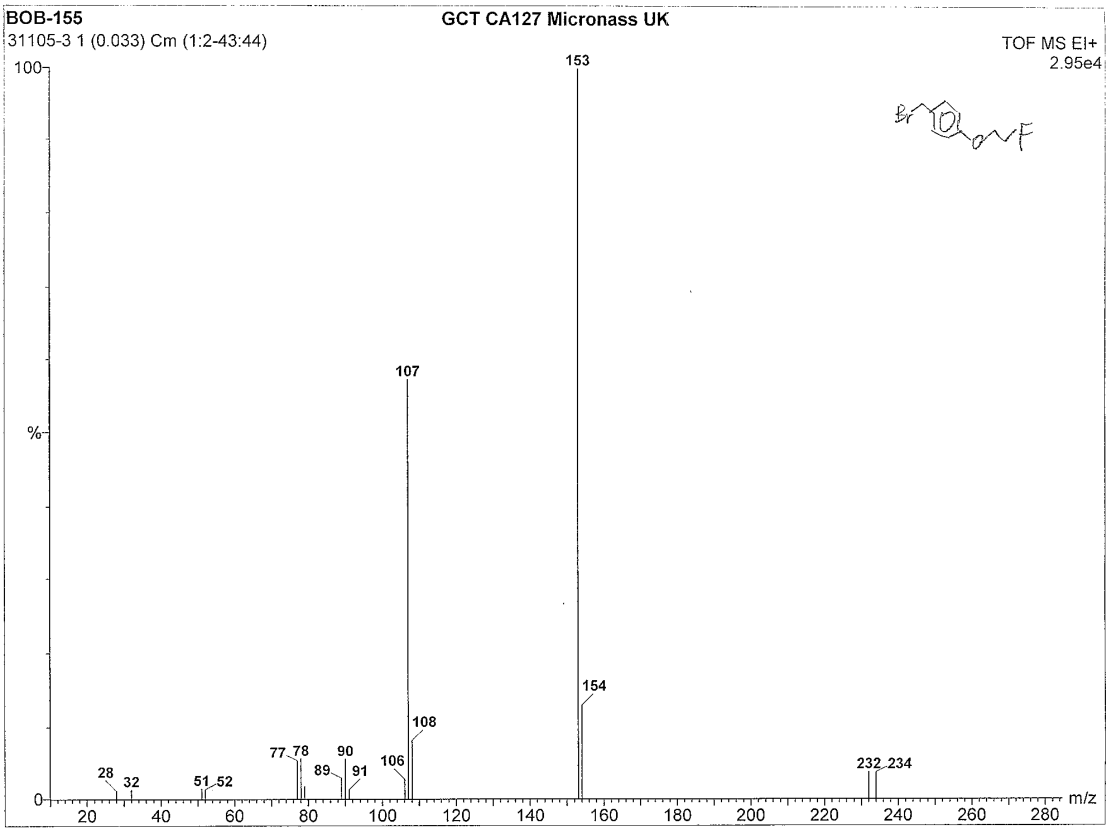

MS spectrum of 1-(bromomethyl)-4-(2-fluoroethoxy)benzene (**11a**)

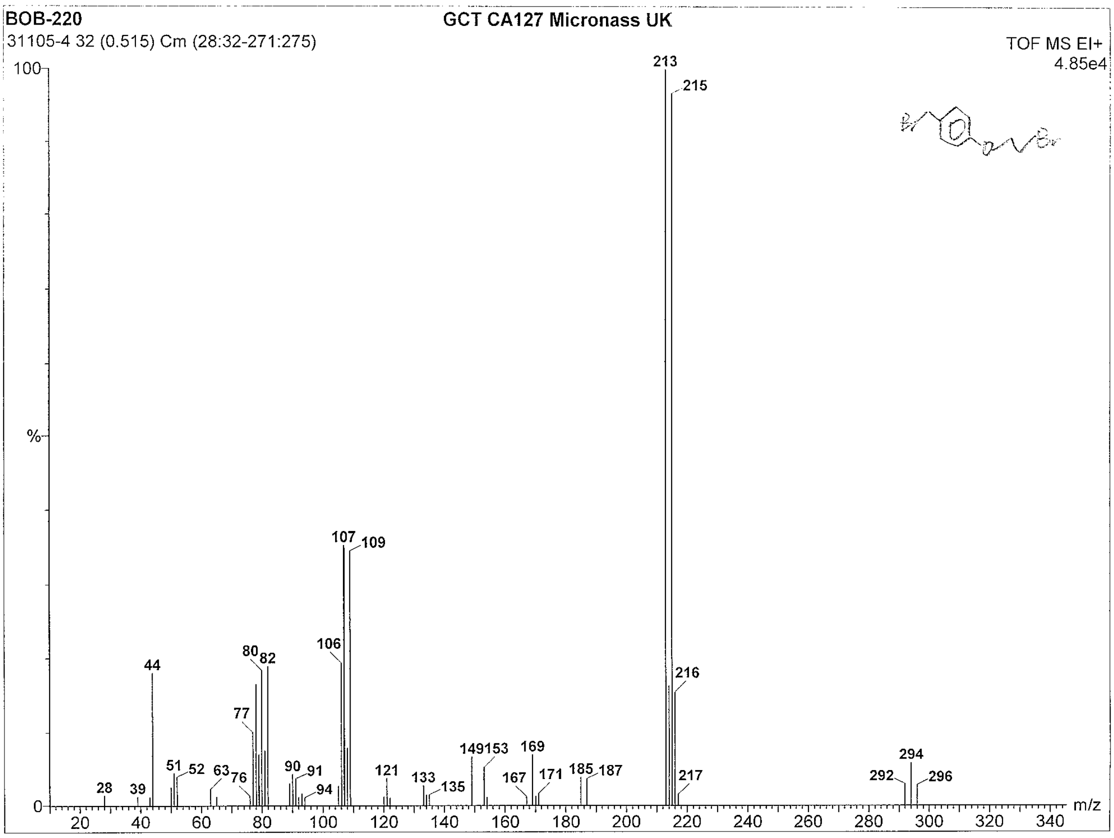

MS spectrum of 1-(2-bromoethoxy)-4-(bromomethyl)benzene (**11b**)

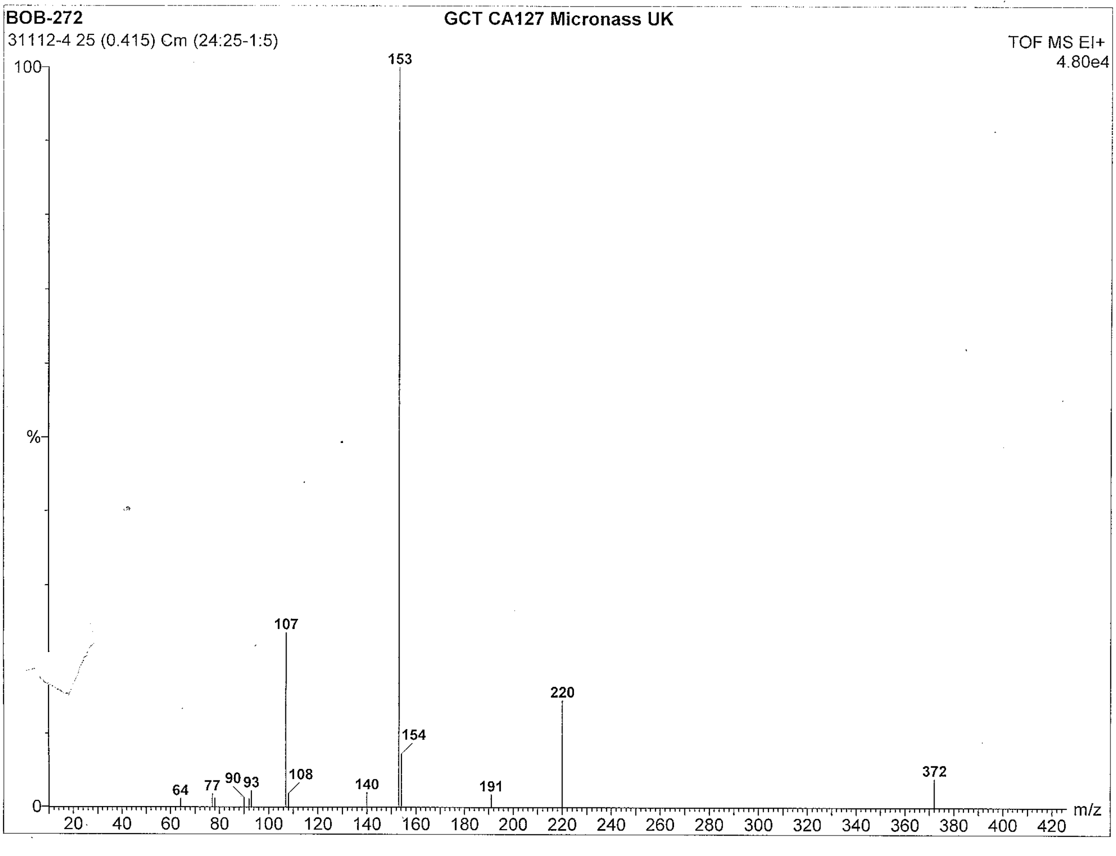

MS spectrum of 1-(2-fluoroethoxy)-4-((4-iodophenoxy)methyl)benzene (**12a**)

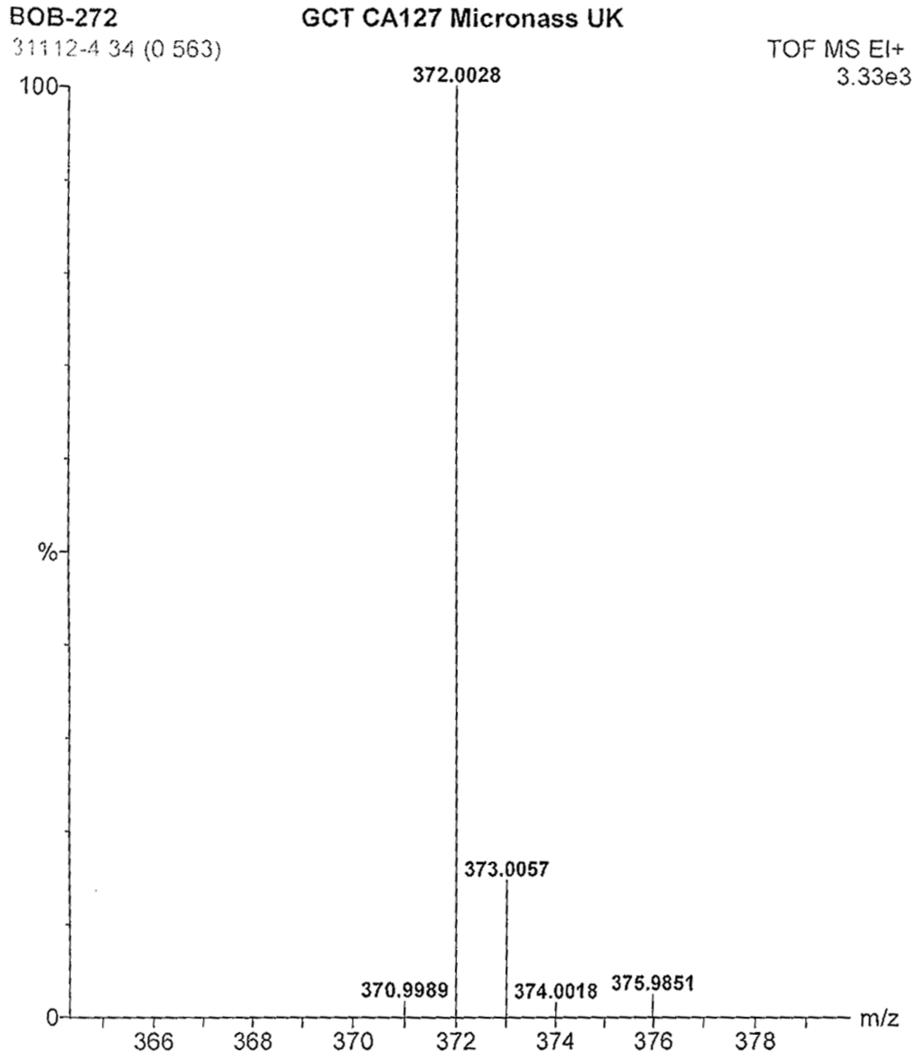

Elemental Composition Report

Tolerance = 10.0 PPM / DBE: min = -1.5, max = 50.0
Isotope cluster parameters: Separation = 1.0 Abundance = 1.0%

Monoisotopic Mass, Odd and Even Electron Ions
82 formula(e) evaluated with 3 results within limits (up to 50 closest results for each mass)

Minimum:	80.00				-1.5		
Maximum:	100.00		200.0	10.0	50.0		
Mass	RA	Calc. Mass	mDa	PPM	DBE	Score	Formula
372.0028	100.00	372.0023	0.5	1.5	8.0	2	C15 H14 O2 F I

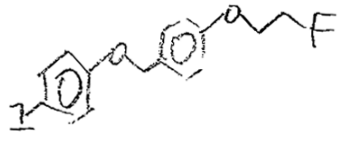

HRMS spectrum of 1-(2-fluoroethoxy)-4-((4-iodophenoxy)methyl)benzene (**12a**)

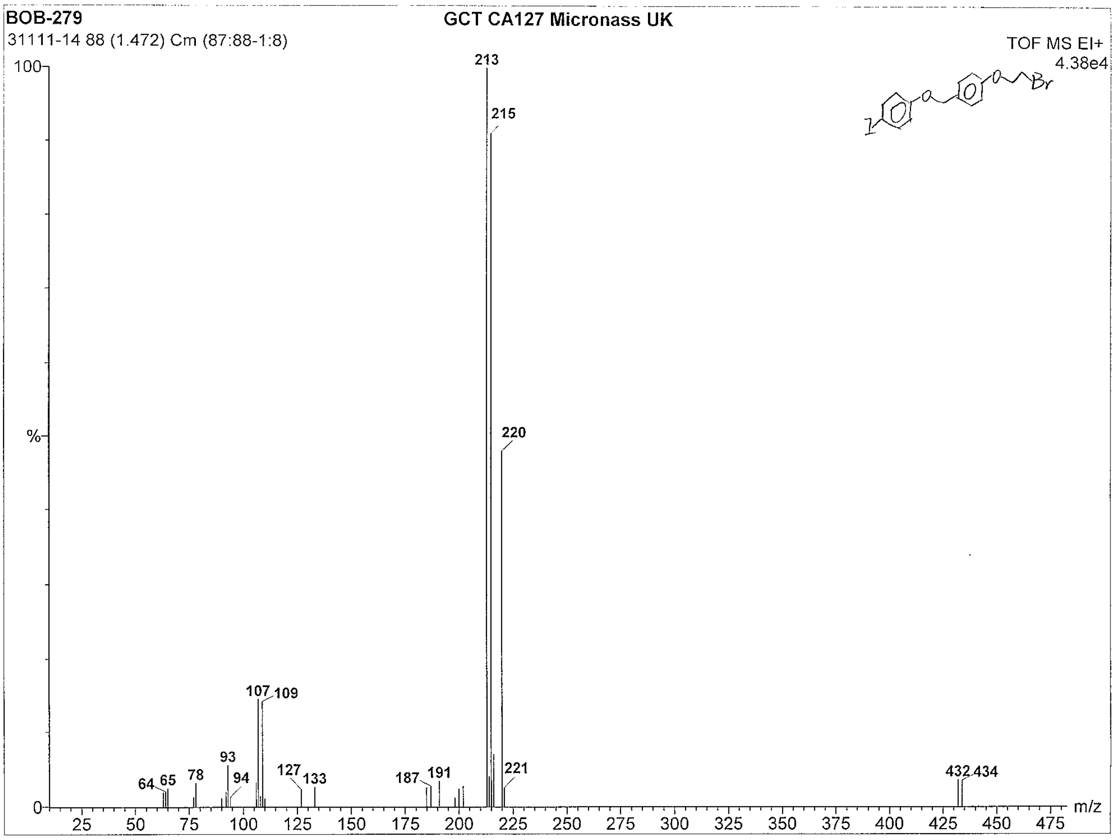

MS spectrum of 1-(2-bromoethoxy)-4-((4-iodophenoxy)methyl)benzene (**12b**)

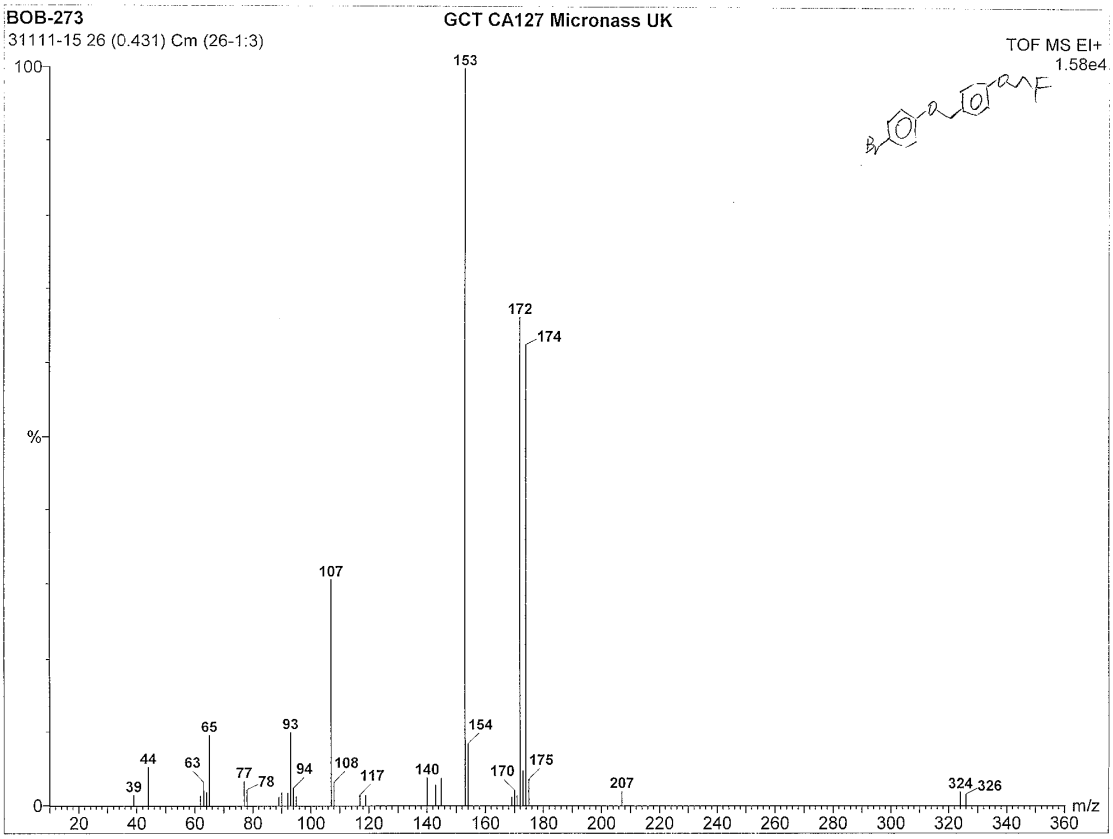

MS spectrum of 1-bromo-4-((4-(2-fluoroethoxy)benzyl)oxy)benzene (**12c**)

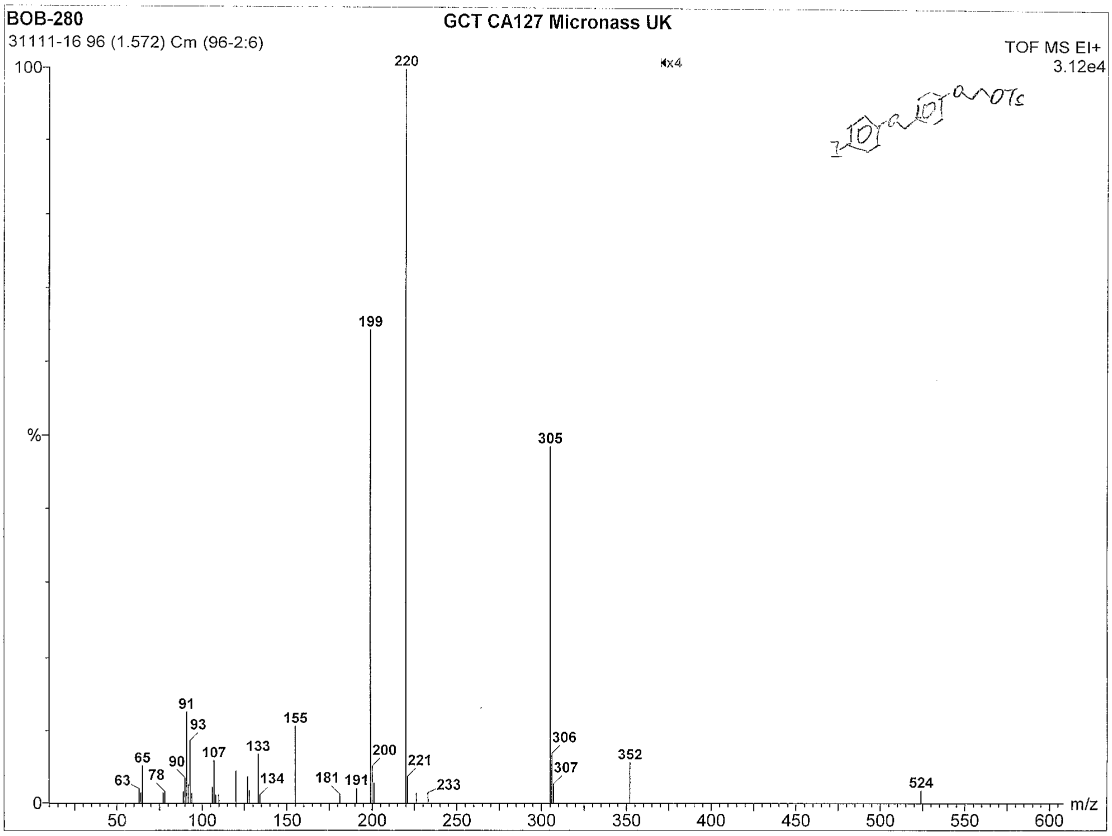

MS spectrum of 2-(4-((4-iodophenoxy)methyl)phenoxy)ethyl 4-methylbenzenesulfonate (**13a**)

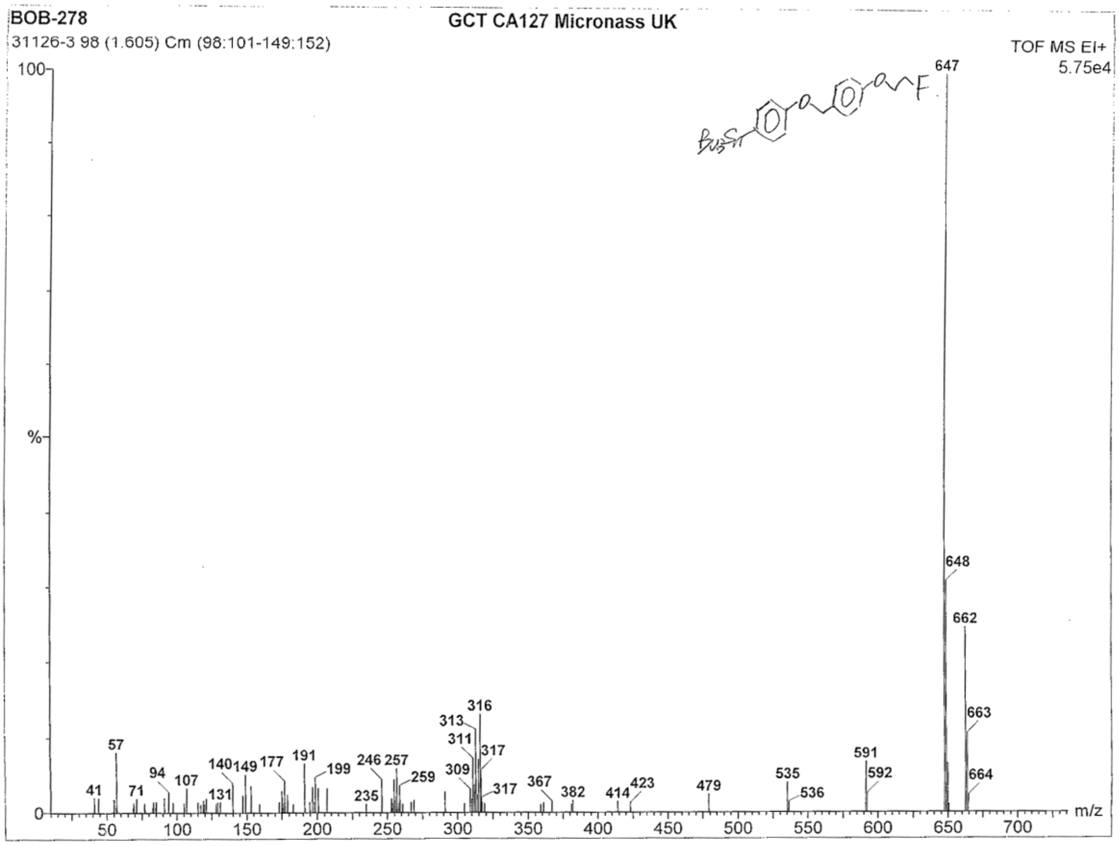

MS spectrum of tributyl(4-((4-(2-fluoroethoxy)benzyl)oxy)phenyl)stannane (**13b**)
